# Tetrazine-*trans*-cyclooctene ligated lanthanide conjugates for biomedical imaging

**DOI:** 10.1039/d5qi01745a

**Published:** 2025-10-14

**Authors:** Hongxuan Chen, William Lim Kee Chang, Grace T. McMullon, Yichao Yu, Benjamin P. Woolley, Gráinne Geoghegan, Ceren Yalcin, Sophie V. Morse, Mark F. Lythgoe, James J. Choi, Nicholas J. Long

**Affiliations:** a Department of Chemistry, Imperial College London, Molecular Sciences Research Hub London W12 0BZ UK n.long@imperial.ac.uk; b Department of Bioengineering, Imperial College London London SW7 2AZ UK; c UCL Centre for Advanced Biomedical Imaging, University College London Paul O'Gorman Building London WC1E 6DD UK; d Department of Brain Sciences, Imperial College London Hammersmith Campus London W12 0NN UK; e UK Dementia Research Institute at Imperial College London UK

## Abstract

Lanthanide chelates and copper-free ‘click’ chemistry have important uses for targeted molecular imaging and therapeutic strategies. Herein, we report the complexation of lanthanides to a tetrazine-functionalised DO3A macrocyclic chelator and the tetrazine-TCO ligation between the lanthanide(DO3A-tetrazine) complexes and a TCO-PEG_4_-functionalised rhodamine as a model agent. The luminescent and magnetic properties of the resultant dual-modal conjugates are described. The tetrazine moiety was found to sensitise terbium luminescence, resulting in a ‘turn-off’ effect upon its transformation to the dihydropyridazine linker, with the rhodamine moiety then dominating the fluorescence emissions. The *T*_1_ relaxivities of Gd(DO3A-tetrazine) and Gd(DO3A-PEG_4_-rhodamine) were found to be similar to [Gd(DOTA)]^−^ (Dotarem®). As a proof-of-concept *in vivo* test, the click conjugates were delivered to mice brains using the combination of focused ultrasound and microbubbles, with neuron uptake of the probes observed.

## Abstract 2

Tetrazine-functionalised lanthanide chelates and their catalyst-free ‘click’ reactions with a *trans*-cyclooctene-rhodamine fluorophore are reported. These probes were delivered across the blood–brain barrier using focused ultrasound and microbubbles.

## Graphical abstract

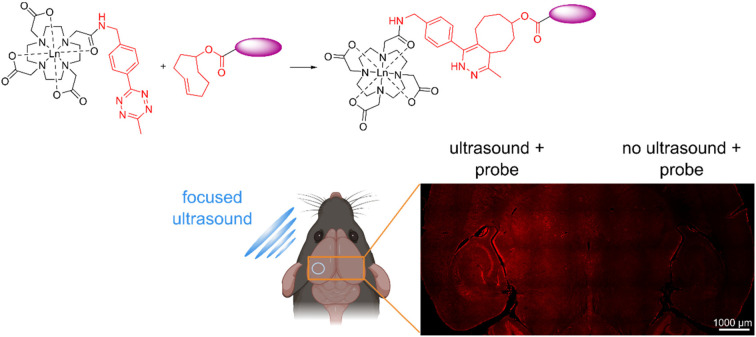

## Introduction

Simple and reliable methods for covalently linking molecular entities are essential in the development of targeted and multi-modal molecular imaging and therapeutic strategies. Covalent linkages are important for the introduction of targeting vectors to maximise signal at the sites of interest and minimise off-target responses and toxicity.^1,2^ Covalent linkages also enable the introduction of additional imaging modalities,^3,4^ therapeutic loads^5,6^ or chemical modifiers to optimise the physical^7^ and biological properties of imaging agents towards their specific applications.^8,9^

The use of catalyst-free ‘click’ chemistry has received much interest for the modular combination of molecular fragments, with fast kinetics under mild conditions and the absence of toxic by-products.^10–14^ The exclusion of non-reactants simplifies purification procedures and is necessary where additional reagents would interact adversely with molecular components or biological systems. Such scenarios include the undesired chelation of catalytic copper in place of a desired radiometal^15^ or changes in pH leading to the disruption of biomolecular structure and activity.^16,17^

The inverse-electron demand Diels–Alder (IEDDA) reaction between *trans*-cyclooctene (TCO) and tetrazine has been demonstrated to be a highly effective and versatile metal-free conjugation method for biomedical applications.^18^ TCO-tetrazine conjugation has demonstrated high specificity and bioorthogonality, as well as fast kinetics (10^3^–10^6^ M^−1^ s^−1^) in biocompatible solvents at dilute concentrations (10 nM).^19–21^ Upon formation of the stable 1,4-dihydropyridazine linker and its subsequent oxidation to pyridazine, N_2_ is released as the sole by-product.^22^ TCO-tetrazine conjugation is highly versatile for biological applications, with recent examples including radiolabelled microbubbles,^23^ radioimmunoconjugates in a pre-targeting approach,^24–26^ and the *in vivo* labelling of biomarkers in cells^27,28^ and in organisms.^29^

The lanthanide series holds several roles in biomedical applications. Several lanthanide isotopes are currently in clinical use or have demonstrated promise as radionuclides for: positron emission tomography (lanthanum-132, lanthanum-133, terbium-152);^30–32^ alpha therapy (terbium-149),^33^ beta therapy (terbium-161, lutetium-177)^34,35^ and Auger therapy (terbium-161).^34^ Gadolinium chelates are widely used as longitudinal (*T*_1_) contrast agents in magnetic resonance imaging (MRI), while dysprosium(iii) holds potential as a high-field transverse (*T*_2_) contrast agent through Curie spin relaxation and as paramagnetically shifted (PARASHIFT) agents for MRI.^36,37^ Europium and terbium have been used as luminescent optical imaging probes,^38^ with targeting moieties in some cases serving a dual purpose as a sensitiser for long-lived luminescence.^39,40^ Luminescence emission from lanthanide centres can be made switchable, as demonstrated by Zheng *et al.* using a terbium-quinolinone-tetrazine probe for luminogenic metabolic labelling.^41^ In this example, the IEDDA reaction of the tetrazine moiety with a TCO-glycan resulted in the loss of quenching by luminescence resonance energy transfer by the tetrazine moiety and resulted in ‘turn-on’ sensitised terbium emission.^41^

Free lanthanide ions have exhibited toxic effects, interfering with signal pathways mediated by the similarly sized Ca^2+^ ion,^42^ and gadolinium deposition in the brain has been reported.^43,44^ Effective macrocyclic chelators that can bind the metal centre without impinging on their functionality^45^ are therefore essential for their use in biological systems.

The dodecane tetraacetic acid (DOTA) macrocycle has been extensively used as a lanthanide chelator, forming stable octadentate complexes through its four amine and four acetate moieties. The [Gd(DOTA)]^−^ complex is currently in widespread clinical use as the MRI contrast agent, gadoteric acid (Dotarem®).^46^ A bifunctional analogue of DOTA, 1,4,7,10-tetraazacyclododecane-1,4,7-triacetic acid (DO3A), including coordination from a carbonyl in the amide linker to maintain octadentate coordination, has also been employed as a chelator for Ln^3+^ ions and the chemically-similar Y^3+^ ion.^47–50^ Notable examples of its clinical use include lutetium-177 vipivotide tetraxetan for the treatment of prostate-specific membrane antigen (PSMA)-positive tumours,^47^ lutetium-177 oxodotreotide for the treatment of neuroendocrine tumours^48^ and yttrium-90, terbium-161 or lutetium-177 edotreotide for the treatment of somatostatin receptor-positive tumours.^49,50^

In 2013, Evans *et al.* reported the development of tetrazine-functionalised DOTA-based chelates for gallium-68, and subsequently demonstrated their use for the radiolabelling of the epidermal growth factor receptor-selective monoclonal antibody, Cetuximab, using both pre-targeting and direct conjugation strategies.^51,52^ Recently, we have reported the synthesis and properties of Gd, Eu and Tb(DO3A-tetrazine) and the IEDDA conjugation of the Gd(DO3A-tetrazine) with a cyclic RGD peptide shuttle.^53^

**Scheme 1:**
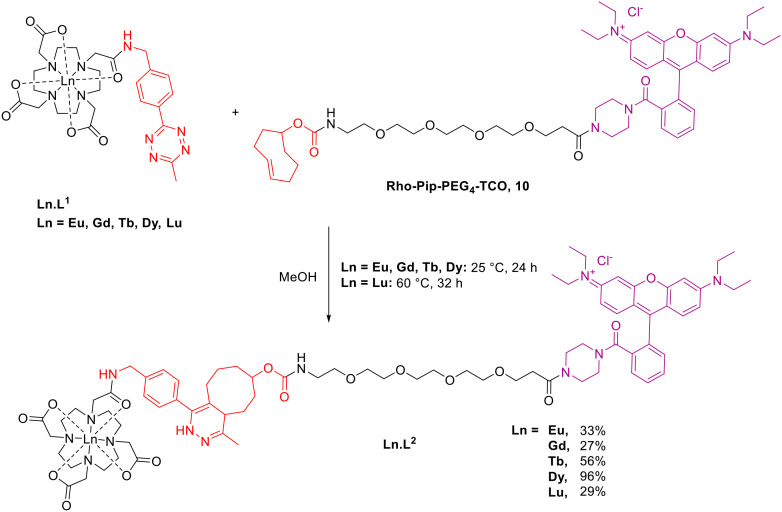
Synthesis of biorthogonal click reaction between Ln-tetrazine complex, Ln.L^1^ and Rho-Pip-PEG_4_-TCO, 10.

In this work, we report the synthesis of Dy and Lu(DO3A-tetrazine), the conjugation of these, and previous, Ln complexes (Ln = Eu, Gd, Tb)^53^ to a TCO-functionalised rhodamine fluorophore as a model agent (Scheme 1), and the luminescent and magnetic properties of the complexes and conjugates. An ‘always-on’ rhodamine-piperazine fluorescent tag was used as the complementary TCO-functionalised fragment. This resulted in analogous structures to the dual-modal MRI/optical Gd(DO3A-rhodamine) and Gd(DO3A-pip-rhodamine) probes we have previously reported for pH-responsivity within tumour cells and for neuron labelling respectively.^54,55^ In place of an ethylene bridge or direct amide coupling, a poly(ethylene glycol) (PEG) linker was incorporated due to its use in conjugates for biological applications as a water-soluble, inert, and biocompatible linker.^56^ This approach illustrates a simple biorthogonal introduction of a fluorescent tag which could be extended to targeting groups or alternative imaging modalities.

**Fig. 1:**
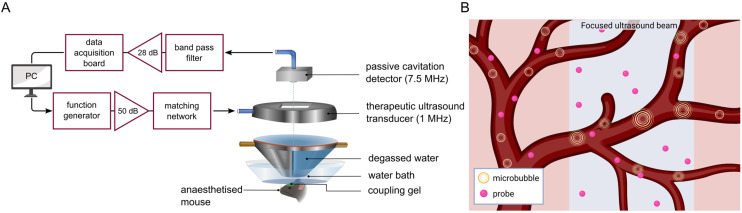
(A) *In vivo* experimental set-up for the focused ultrasound-assisted delivery of Ln.L^2^ to the mouse brain. (B) Radially-oscillating microbubbles in response to ultrasound exert mechanical stress on the cerebral vasculature to disrupt the blood–brain barrier and enable the crossing of probes into the brain parenchyma. Created in Inkscape and https://BioRender.com.

To demonstrate their potential as dual-modal probes for *in vivo* imaging applications, we administered the conjugates to mice and carried out post-mortem assessments of its uptake in the brain and its clearance through the kidneys and liver. Analogous to our previous study,^55^ we have delivered enhanced concentrations of our probes to the brain using the combination of focused ultrasound and microbubbles as most compounds larger than 400–500 Da are otherwise unable to enter the brain parenchyma.^55,57,58^ This non-invasive technique involves the ultrasound-driven oscillations of systemically-administered microbubbles to exert mechanical stress on the cerebral vasculature and transiently enhance blood–brain barrier permeability (Fig. 1).^59^ This proof-of-concept study aims to demonstrate the potential of tetrazine-TCO ligation for the modular and biocompatible syntheses of lanthanide-based conjugates towards biomedical applications.

## Results and discussion

### Synthesis of lanthanide complexes

The octadentate DO3A-tetrazine macrocycle, L^1^, was synthesised as previously reported (Schemes S1 and S2).^51,53,60^ The final addition of the chloroacetyl tetrazine arm to the *t*Bu_3_DO3A·HBr macrocycle was carried out under milder conditions using NaHCO_3_ (7 equiv.) and KI (0.1 equiv.) at 50 °C for 3 days, which resulted in reduced decomposition relative to previous observations.^53^ The macrocycle was deprotected by TFA, followed by lanthanide complexation. The rhodamine-TCO moiety, 10, was synthesised from an ‘always on’ rhodamine-piperazine, 9, and a TCO-PEG_4_-NHS ester.

The rhodamine-piperazine was synthesised as previously reported from rhodamine B and 1-Boc-piperazine by amide coupling using HBTU and triethylamine, followed by a subsequent TFA-catalysed Boc deprotection (Scheme S3).^61,62^ The rhodamine-TCO moiety, 10, was formed by NHS-ester activated amide coupling between the commercially-available TCO-PEG_4_-NHS and rhodamine-piperazine in anhydrous chloroform (Scheme S4).

### Bioorthogonal ‘click’ chemistry

The final complexes were synthesised by IEDDA cycloaddition between Rho-Pip-PEG_4_-TCO, 10, and Ln(DO3A-tetrazine), Ln.L^1^, in methanol at 25 °C (Scheme 1). Despite literature reports of the tetrazine-TCO cycloaddition reaching completion in as little as 15 minutes,^52^ we found the reaction between the two moieties to be slow. The reaction was monitored by LCMS and found to require at least 24 hours before full consumption of the Rho-Pip-PEG4-TCO starting material. These observations were most likely a result of low concentrations of reaction mixtures (5–10 mM) or potential isomerisation of the *trans*-cyclooctene to the less reactive *cis*-isomer prior to use.

### Photophysical spectroscopy

**Fig. 2:**
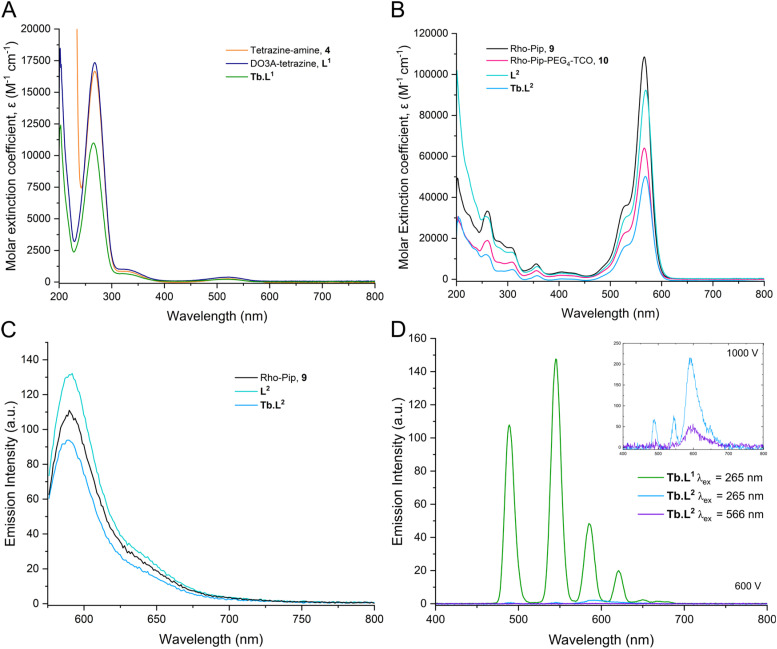
Absorbance spectra of the tetrazine precursors, 4, L^1^ and Tb.L^1^ at 20 μM, *λ*_abs_ = 265 nm (A) and the rhodamine precursors, 9 and 10 and click products, L^2^ and Tb.L^2^ at 5 μM, *λ*_abs_ = 566 nm (B) in PBS buffer (pH = 7.4). (C) Fluorescence spectrum of the rhodamine precursor and click products 5, L^2^ and Tb.L^2^ when *λ*_ex_ = 566 nm at 5 μM in PBS buffer (pH = 7.4). (D) Phosphorescence spectrum of the Tb complexes prior- (Tb.L^1^, *λ*_ex_ = 265 nm) and post-IEDDA cycloaddition (Tb.L^2^) excited at 265 nm and 568 nm at 20 μM in PBS (pH = 7.4) at 600 V and 1000 V.

The absorbance spectra of the tetrazine precursors in PBS (pH = 7.4) all exhibit absorbance at 265–268 nm and a shoulder at 330 nm (Fig. 2A), which each correspond to separate π–π* transitions.^63,64^ The absorbance at 526 nm arises from the n–π* transition and the observed pink colour of the complexes. Following cycloaddition, the absorbance profiles for the click products, M.L^2^, are dominated by the rhodamine absorbance at 566 nm (Fig. 2B) and these compounds have molar extinction coefficients of 44 500–90 800 M^−1^ cm^−1^ in PBS (Table S3). This is in the expected region, given that rhodamine B has a molar extinction coefficient of 106 000 M^−1^ cm^−1^.^65^ Analogously, the fluorescence spectra of the click products exhibited fluorescence solely arising from the rhodamine moiety, *λ*_em_ = 590 nm (Fig. 2C). All click products, M.L^2^, display similar fluorescence quantum yields (*φ*_F_) in the range of 0.17–0.2, determined relative to Rhodamine B, which has a *φ*_F_ = 0.5 in ethanol (Table S3).^66,67^

The long-lived photophysical emissions of the terbium and europium complexes were investigated by recording time-gated luminescence spectra (Fig. 2D and Fig. S2D). When excited at *λ*_ex_ = 265 nm through the tetrazine moiety, the Tb(DO3A-tetrazine) complex, Tb.L^1^, displayed characteristic green terbium emission corresponding to the ^5^D_4_ → ^7^F_*J*_ transitions, indicating that the tetrazine moiety can act as a weak lanthanide sensitiser.^53^ This is expected as the absorption of the tetrazine (19 011 cm^−1^) lies below the energy levels of the excited states for Tb^3+^ (^5^D_4_ 20 500 cm^−1^)^68^ and Eu^3+^ (^5^D_1_ and ^5^D_2_ 19 000–21 000 cm^−1^).^69^ Weak sensitisation can be rationalised by the useful guideline for effective energy transfer, which requires that the lowest energy triplet state of the sensitiser is around 1000–2000 cm^−1^ higher than that of the lanthanide emissive state. This facilitates efficient transfer from the ligand triplet state to the excited lanthanide state whilst limiting competitive back energy transfer.^70^ Moreover, there are several reports of tetrazines acting as fluorescence quenchers for both inorganic and organic dyes.^41,71,72^

Following IEDDA cycloaddition, the tetrazine is converted into a 1,4-dihydropyridazine, which is no longer able to act as a sensitiser for terbium. This is demonstrated by the almost complete loss of terbium emission for Tb.L^2^ (Fig. 2D) following IEDDA when excited at 265 nm. This was also observed for the europium analogues, Eu.L^1^ and Eu.L^2^, where the red europium emission, corresponding to the ^5^D_0_ → ^7^F_*J*_ transition, is lost following cycloaddition (Fig. S2D). Previous work by our group has demonstrated the ability of the rhodamine moiety to act as an efficient sensitiser when excited at 310 nm.^54^ We have also previously demonstrated lanthanide emission sensitisation by Quin C1, an organic formyl peptide receptor 2/lipoxin A4 receptor (FPR2/ALX)-targeting group, and luminescence resonance energy transfer (LRET) from the terbium centre to rhodamine.^39^ The distance-dependency of this mechanism of energy transfer aligns with our observations in this case that the rhodamine is unable to sensitise the terbium centre when excited at *λ*_ex_ = 566 nm. The distance between the two moieties in Tb.L^2^ is greater than in the work previously reported, and LRET is therefore poor.^39^ Nevertheless, the presence of a ‘clickable’ tetrazine moiety in proximity to a lanthanide chelator has been shown to modulate the emission properties of the metal,^41^ and can likely be further exploited in other modular systems.

### Relaxometric properties

The relaxivity (*r*_1_) of the Gd complexes were measured using a 0.25 T Fast Field Cycling NMR relaxometer at frequencies from 0.01 to 10 MHz; while the relaxivity at 400 MHz was recorded on a 9.4 T MRI preclinical scanner in 0.5 mL solutions in PBS (pH = 7.4) in 3D-printed moulds. The effective Gd^3+^ ion concentration of samples was determined from bulk magnetic susceptibility shifts in ^1^H NMR spectra.^73,74^

**Fig. 3:**
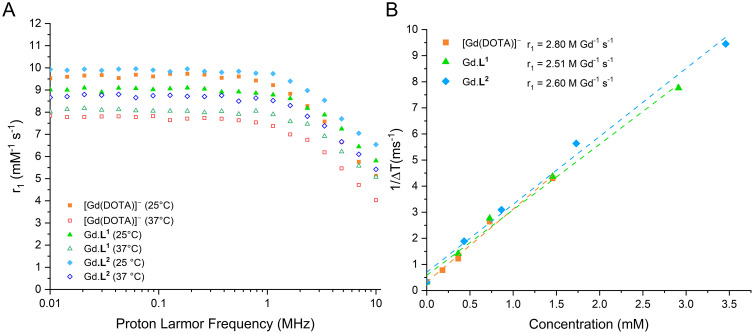
(A) NMRD profiles for Gd complexes before and after IEDDA cycloaddition compared to Gd-DOTA, at 25 °C (solid symbols) and 37 °C (hollow symbols); (B) longitudinal relaxivity of Gd complexes at 9.4 T (400 MHz) for phantoms of 1 mL solution in PBS buffer at different concentrations at 25 °C. Concentrations corrected for Gd concentration using bulk magnetic susceptibility shifts.^73,74^

From the ^1^H NMR spectra obtained for the Eu and Tb complexes of L^1^ (Fig. S20 and S21) and L^2^ (Fig. S30 and S31), we observed that the Ln(DO3A-tetrazine), Ln.L^1^, and Ln(DO3A-PEG_4_-rhodamine), Ln.L^2^, were present as the SAP conformation. This indicates that the less favourable conformation for Gd-based contrast agents for MRI compared with TSAP analogues. TSAP conformers have previously demonstrated approximately 50 times faster water exchange at the Gd centre, allowing relaxation to be transferred more rapidly to the bulk water.^74^ Although previous work recorded lifetimes for Eu.L^1^ and Tb.L^1^ and calculated the hydration number, *q* = 1,^53^ the hydration numbers for Ln.L^2^ could not be determined due to the loss of lanthanide emission upon conjugation. However, given that the lanthanide coordination environment was unchanged in the formation of Ln.L^2^ and remained octadentate, it is assumed that hydration number of Ln.L^2^ conjugates would remain as 1, with one coordination site for H_2_O. Measurements recorded on the fast field cycling relaxometer at 1 MHz at 25 °C show relaxation rates in the order of Gd.L^2^ > Gd-DOTA > Gd.L^1^ (Fig. 3A). The minor differences are likely due to the greater molecular weight of Gd.L^2^ and, therefore, its reduced rotational correlation time. However, these differences become insignificant at a higher magnetic field strength, with minimal differences in the relaxivities between the three Gd complexes when measured with a 9.4 T MRI scanner (400 MHz) at 25 °C (Fig. 3B). This suggests that the relaxometric properties and expected MRI properties of the tetrazine-functionalised and conjugated Gd complexes are comparable to the Gd-DOTA clinical standard (Table 1).

**Table 1:** Longitudinal relaxivity data for Gd complexes at 25 °C and 37 °C

Complex	Temperature (°C)	*r*_1_ (mM^−1^ s^−1^)		
		0.01 MHz	10 MHz	400 MHz
Gd.L^1^	25	9.32	6.14	2.51
	37	8.44	5.28	—
Gd.L^2^	25	10.11	7.44	2.60
	37	9.68	6.80	—
Gd-DOTA	25	9.53	5.13	2.80
	37	7.84	4.03	—

### Ultrasound-assisted *in vivo* delivery to mouse brains

The click conjugates, Gd.L^2^, Tb.L^2^ and Lu.L^2^ were intravenously injected into wild-type mice (*n* = 5) and delivered to the left hemisphere of mouse brains using the combination of focused ultrasound and microbubbles. For the deliveries of Tb.L^2^ and Lu.L^2^, a peak-negative ultrasound pressure of 0.80 MPa was used, accounting for an 11% attenuation by the mouse skull,^75^ and a dose of 15.6 mg kg^−1^ was administered. 30 μm-thick formalin-fixed frozen brain sections were subsequently analysed by fluorescence microscopy. In the case of Gd.L^2^, a lower peak-negative ultrasound pressure of 0.53 MPa was emitted and the dose of Gd.L^2^ was doubled to 31.1 mg kg^−1^. *Ex vivo* MRI scans using T1 Flash 3D and 2D, T1 mapping and T2 TurboRARE 3D sequences were first performed on mice heads prior to fluorescence imaging of brain sections.

**Fig. 4:**
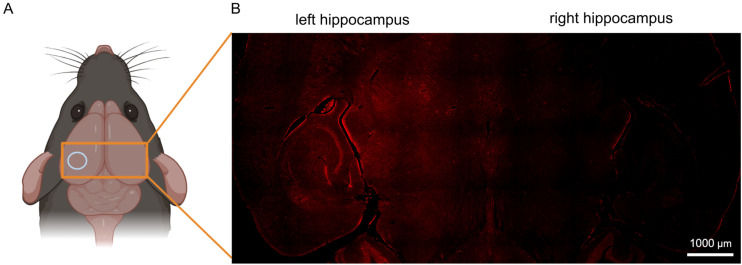
(A) The left hippocampus of mouse brains was targeted with the focused ultrasound beam, highlighted as a light blue circle. (B) Fluorescence image showing both the left targeted and right control hippocampi following intravenous injection with Lu.L^2^ (orange rectangle in part A). Created in Inkscape and https://BioRender.com.

**Fig. 5:**
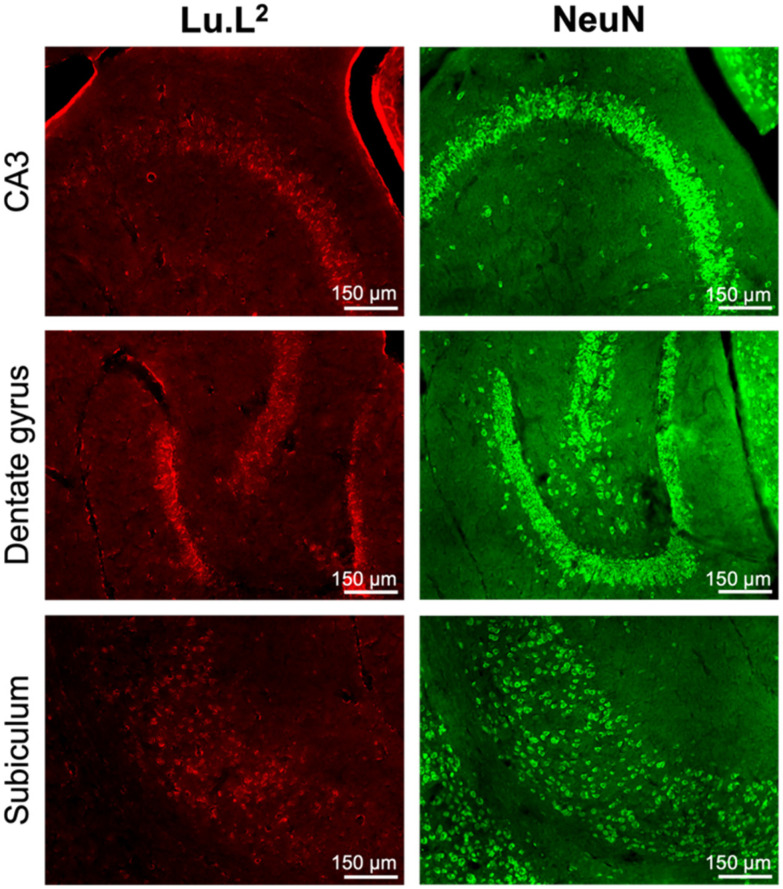
(Left) Fluorescence images, prior to immunofluorescence staining, of horizontal, 30 μm-thick formalin-fixed frozen brain sections from mice injected with Lu.L^2^ and treated with focused ultrasound and microbubbles and (Right) NeuN-positive staining of horizontal 30 μm-thick frozen sections from the same brain.

The successful ultrasound-assisted disruption of the blood–brain barrier and delivery of Tb.L^2^ and Lu.L^2^ to mouse brains was evidenced by the detection of fluorescence signals from the rhodamine tag in the left hippocampus and thalamus (Fig. 4). Fluorescence signals with intensities greater than the background level were not observed in the contralateral right hemisphere control region, which was not exposed to the ultrasound beam and where blood–brain barrier permeability was not enhanced. Neuronal uptake in the ultrasound-treated region was observed and was particularly prominent in the dentate gyrus, layers V and VI of the entorhinal cortex and cerebellar granule cells (Fig. 5 and S35), with the identity of fluorescent cells as neurons confirmed by NeuN staining (Fig. S38–S40). These observations mirror those in our previous study with an amide-coupled Gd(DO3A-pip-rhodamine) analogue, with the cationic and lipophilic nature of the rhodamine moiety enabling permeation of the cell membrane and its uptake by neurons.^55^

However, in the case of Gd.L^2^, gadolinium-enhanced MR contrast was not detected, with no expected hyperintensity discernible in the region of the brain exposed to the ultrasound beam (Fig. S34). Although neuron uptake was observed in the entorhinal cortex and cerebellum in the left hemisphere, the intensity and area of fluorescence at these sites were much-reduced compared to the *in vivo* deliveries of Tb.L^2^ and Lu.L^2^. Rhodamine fluorescence signals were detected in kidney and liver sections from mice injected with Gd.L^2^ and treated with focused ultrasound and microbubbles, but were absent in kidney (Fig. S36) and liver (Fig. S37) sections from a control mouse that was not injected with Gd.L^2^ but was treated with focused ultrasound and microbubbles only. This suggested rapid clearance through renal and hepatic pathways within approximately 10 minutes following intravenous injection.

We speculate that the reduced fluorescence signals from Gd.L^2^ observed in the brain compared with Tb.L^2^ and Lu.L^2^ are due to an increased washout of the unfixed probe from the tissues. Brain samples were immersed in solution for a longer duration (an additional 10 days on average) as part of the MR imaging procedure and underwent an increased number of solution changes. The absence of MR enhancement also suggests that the reduced sensitivity of MRI compared with fluorescence imaging requires increased gadolinium doses for MR enhancement, which may not be attainable using the probe and *ex vivo* MR imaging technique used in this study.

## Conclusions

This proof-of-concept work describes the use of tetrazine-TCO ligation for synthesising dual-modal lanthanide probes in a biocompatible and modular strategy. Although MRI/optical imaging was explored here, this conjugation method can be translated to lanthanide-based radiolabelling or the introduction of targeting moieties. The inclusion of the PEG chain enabled the click products, Ln.L^2^, to be soluble in PBS (pH = 7.4) for *in vivo* administration. To improve the applicability of this IEDDA conjugation, the stability of any TCO-bearing reagents would need to be investigated to ensure the cyclooctene remains as the *trans*-isomer to minimise reaction times, which would be especially pertinent to radiochemistry and pre-targeting applications.

The focused ultrasound-assisted delivery of the click products, Ln.L^2^, to the mouse brain and their neuronal uptake demonstrated their potential for *in vivo* applications. Enhanced doses were delivered to the targeted left hemisphere over the contralateral control hemisphere. The relaxivity of the Gd.L^2^ complex was too low to be observed in mouse brains using *ex vivo* MRI, although rhodamine fluorescence from the Tb.L^2^ and Lu.L^2^ complexes was observed.

Overall, tetrazine-TCO ligation is a useful method of conjugation to produce lanthanide-based molecular imaging and therapeutic agents. Further applications will explore the use of luminescence quenching following ligation as a switchable probe and the use of *in vivo* pre-targeting strategies.

## Experimental

### Synthesis of ‘Click’ conjugates, L^2^ and Ln.L^2^

#### DO3A-PEG_4_-piperazine-rhodamine (L^2^)

L^1^ (7.2 mg, 12.1 μmol, 1.1 eq.) and 10 (9.8 mg, 11.0 μmol, 1 eq.) were dissolved in H_2_O (1.25 mL). The solution was stirred at 60 °C for 7 hours. The solvent was removed under vacuum. The crude product was purified by HPLC using prep HPLC gradient method 2 (Table S2). After lyophilisation, a dark purple solid (2.9 mg, 2.0 μmol, 18%) was obtained; ESI-LRMS (ES+): [C_78_H_104_N_12_O_16_]^2+^, (+2) *m*/*z* 734.9, ESI-HRMS (ES+): anal. For [C_78_H_104_N_12_O_16_]^2+^ [M + 2H]^2+^ calcd: 734.9037, found: 735.3635; UV-Vis (PBS, *λ*_max_/nm): 569; *t*_R_ (prep) = 12.71–12.80 min.

#### Eu(DO3A-PEG_4_-piperazine-rhodamine) (Eu.L^2^)

Eu.L^1^ (10.0 mg, 0.011 mmol, 1.0 eq.) and 10 (12.2 mg, 0.017 mol, 1.5 eq.) were dissolved in MeOH (0.5 mL). The solution was stirred at 25 °C for 24 hours. The solvent was removed under vacuum. The crude product was dissolved in 3 : 1 H_2_O : MeCN and purified by HPLC using prep HPLC gradient method 1 (Table S1). After lyophilisation, a dark purple solid Eu.L^2^ (5.8 mg, 3.6 μmol, 33%) was obtained; ^1^H NMR (400 MHz, CD_3_OD) *δ*_H_ (ppm) 38.9, 36.4, 35.6, 34.4, 16.3, 14.1, −1.4, −1.8, −2.4, −3.6, −5.2, −6.4, −6.5, −9.4, −11.2, −12.7, −13.8, −14.3, −17.2, −18.1, −18.2, −18.7, −18.9 (only peaks outside of 0 to 10 ppm reported); ESI-LRMS (ES+): [C_78_H_104_N_12_O_16_Eu]^+^, (+) *m*/*z* 1617.7, ESI-HRMS (ES+): anal. For [C_78_H_104_N_12_O_16_Eu]^+^ [M + H]^+^ calcd: 1617.6906, found: 1617.6907; UV-Vis (PBS, *λ*_max_/nm): 569; *t*_R_ (prep) = 8.26 min.

#### Gd(DO3A-PEG_4_-piperazine-rhodamine) (Gd.L^2^)

Gd.L^1^ (12.1 mg, 16 μmol, 1.0 eq.) was dissolved in H_2_O : MeOH (1 : 1, 2 mL). 10 (15.0 mg, 16 μmol, 1.0 eq.) was added to the solution and stirred at 25 °C for 24 hours. The solvent was removed under vacuum and combined with the reaction in H_2_O. The solvent was removed under vacuum. The crude product was dissolved in 3 : 1 H_2_O : MeCN and purified by HPLC using prep HPLC gradient method 1 (Table S1). After lyophilisation, Gd.L^2^ was obtained as a dark purple solid (7 mg, 4 μmol, 27%); ESI-LRMS (ES+): [C_78_H_104_N_12_O_16_Gd]^2+^, (+2) *m*/*z* 811.9, ESI-HRMS (ES+): anal. For [C_78_H_104_N_12_O_16_Gd]^2+^ [M + 2H]^2+^ calcd: 811.8501, found: 811.8503; UV-Vis (PBS, *λ*_max_/nm): 569; *t*_R_ (prep) = 8.11–8.21 min.

#### Tb(DO3A-PEG_4_-piperazine-rhodamine) (Tb.L^2^)

Tb.L^1^ (12.2 mg, 16.0 μmol, 1.0 eq.) and 10 (10.0 mg, 11.0 μmol, 1.2 eq.) were dissolved in MeOH (0.5 mL). The solution was stirred at 25 °C for 24 hours. The solvent was removed under vacuum. The crude product was dissolved in 3 : 1 H_2_O : MeCN and purified by HPLC using prep HPLC gradient method 1 (Table S1). After lyophilisation, a dark purple solid Tb.L^2^ (14.0 mg, 9.0 μmol, 56%) was obtained; ^1^H NMR (400 MHz, CD_3_OD) *δ*_H_ (ppm) 277.0, 270.1, 255.8, 236.8, 230.3, 135.3, 128.2, 51.5, 45.8, 34.6, 30.3, 22.2, 15.6, 13.7, 13.6, 13.4, 13.0, 12.6, 12.3, 11.7, 11.1, 10.9, −25.2, −32.3, −40.4, −52.1, −61.4, −70.3, −73.3, −80.8, −104.5, −118.1, −125.5, −130.7, −148.4, −221.7, −226.3, −246.5, −259.7, −384.6, −389.5, −396.2, −428.3 (only peaks outside of −10 to 10 ppm reported); ESI-LRMS (ES+): [C_78_H_104_N_12_O_16_Tb]^2+^, (+2) *m*/*z* 812.4, ESI-HRMS (ES+): anal. For [C_78_H_104_N_12_O_16_Tb]^2+^ [M + 2H]^2+^ calcd: 812.3513, found: 812.3514; UV-Vis (PBS, *λ*_max_/nm): 569; *t*_R_ (prep) = 8.20–8.24 min.

#### Dy(DO3A-PEG_4_-piperazine-rhodamine) (Dy.L^2^)

Dy.L^1^ (13.4 mg, 18.0 μmol, 1.0 eq.) was dissolved in MeOH (0.5 mL). 10 (16.1 mg, 18.0 μmol, 1.0 eq.) was added to the solution and stirred at 25 °C for 24 hours. The solvent was removed under vacuum. The crude product was dissolved in 3 : 1 H_2_O : MeCN and purified by HPLC using prep HPLC gradient method 1 (Table S1). After lyophilisation, a purple solid Dy.L^2^ (28.4 mg, 17.4 μmol, 96%) was obtained; ^1^H NMR (500 MHz, CD_3_OD) *δ*_H_ (ppm) 367.9, 347.1, 325.8, 238.0, 226.7, 25.5, −13.9, −14.3, −29.2, −38.1, −43.9, −51.8, −53.4, −60.7, −80.8, −97.3, −100.0, −117.0, −133.8, −159.3, −167.3, −193.4, −221.0 (only peaks outside of −10 to 10 ppm reported); ESI-LRMS (ES+): [C_78_H_104_N_12_O_16_Dy]^+^, (+) *m*/*z* 1628.7, ESI-HRMS (ES+): anal. For [C_78_H_104_N_12_O_16_Dy]^+^ [M + H]^+^ calcd: 1628.6985, found: 1628.6960; UV-Vis (PBS, *λ*_max_/nm): 569; *t*_R_ (prep) = 8.17–8.30 min.

#### Lu(DO3A-PEG_4_-piperazine-rhodamine) (Lu.L^2^)

Lu.L^1^ (7.2 mg, 9.0 μmol, 1.0 eq.) was dissolved in MeOH (5 mL). 10 (8.0 mg, 9.0 μmol, 1.0 eq.) was added to the solution and stirred at 60 °C for 32 hours. The solvent was removed under vacuum. The crude product was purified by HPLC using prep HPLC gradient method 2 (Table S2). After lyophilisation, a purple solid Lu.L^2^ (4.0 mg, 2.4 μmol, 29%) was obtained; ^1^H NMR (500 MHz, D_2_O) *δ*_H_ (ppm) 8.63 (s, 1H), 8.37 (d, *J* = 8.0 Hz, 2H), 7.80 (d, *J* = 7.7 Hz, 2H), 7.63 (t, *J* = 8.4 Hz, 3H), 7.45 (s, 4H), 7.41 (s, 1H), 7.33 (d, *J* = 7.6 Hz, 1H), 7.18 (s, 2H), 6.94 (d, *J* = 9.6 Hz, 2H), 4.63 (t, *J* = 16.3 Hz, 3H), 4.42 (d, *J* = 15.1 Hz, 1H), 4.35 (d, *J* = 14.5 Hz, 2H), 3.93–3.79 (m, 3H), 3.73–3.11 (m, 57H), 2.99–2.36 (m, 42H), 2.30 (d, *J* = 13.3 Hz, 1H), 2.24–2.18 (m, 1H), 2.17–1.92 (m, 4H), 1.92–1.40 (m, 7H), 1.19 (q, *J* = 6.8 Hz, 13H); ESI-LRMS (ES+): [C_78_H_104_N_12_O_16_Lu]^2+^, (+2) *m*/*z* 820.4, ESI-HRMS (ES+): anal. For [C_78_H_104_N_12_O_16_Lu]^+^ [M + H]^+^ calcd: 1639.7101, found: 1639.7040; UV-Vis (PBS, *λ*_max_/nm): 569; *t*_R_ (prep) = 12.70–12.80 min.

## Author contributions

H. C., W. L. K. C. and B. P. W. synthesised the compounds, G. T. M. purified the complexes. H. C., W. L. K. C. and G. T. M. performed the optical spectroscopy and relaxivity measurements. W. L. K. C., G. G. and S. V. M. performed the *in vivo* experiments and W. L. K. C. and C. Y. performed the histology. Y. Y. performed the MRI experiments and Y. Y. and G. T. M. processed the MRI data. N. J. L. supervised the work. All the authors contributed to the preparation and writing of the manuscript.

## Conflicts of interest

There are no conflicts to declare.

## Supplementary Material

QI-013-D5QI01745A-s001

## Data Availability

All the relevant research data is contained within the manuscript and supplementary information (SI). No databases have been used and no references to such databases are contained in the manuscript or SI. Supplementary information is available. See DOI: https://doi.org/10.1039/d5qi01745a.
