# Supplementary material for: Tetrazine-trans-cyclooctene ligated lanthanide conjugates for biomedical imaging
Source: Inorg Chem Front. 2025 Oct 14;13(1):63–73. doi: 10.1039/d5qi01745a (PMC12542886; doi:10.1039/d5qi01745a)
Supplement: QI-013-D5QI01745A-s001 [file QI-013-D5QI01745A-s001.pdf]

Supplementary Information (SI) for Inorganic Chemistry Frontiers.
This journal is © the Partner Organisations 2025

Supplementary Information

Tetrazine-*trans*-cyclooctene ligated lanthanide conjugates for biomedical imaging

Hongxuan Chen,‡[a] William Lim Kee Chang,‡[ab] Grace T. McMullon,‡[a] Yichao Yu,[c] Benjamin P. Woolley,[a] Gráinne Geoghegan,[b,d] Ceren Yalcin,[b] Sophie V. Morse,[b,e] Mark F. Lythgoe,[c] James J. Choi,[b] and Nicholas J. Long*[a]

*Corresponding Author

‡ These authors contributed equally.

[a] Department of Chemistry, Imperial College London, Molecular Sciences Research Hub, White City Campus, Wood Lane, London, W12 0BZ, United Kingdom.

E-mail: n.long@imperial.ac.uk

[b] Department of Bioengineering, Imperial College London, South Kensington Campus, London, SW7 2BP, United Kingdom.

[c] Department of Imaging, Centre for Advanced Biomedical Imaging, University College London, 72 Huntley Street, London, WC1E 6DD, United Kingdom.

[d] Department of Brain Sciences, Imperial College London, Hammersmith Campus, London W12 0NN, United Kingdom.

[e] UK Dementia Research Institute at Imperial College London, United Kingdom.

Table of Contents

1. General Procedures 2
   1.1. Absorption Spectroscopy 2
   1.2. Emission Spectroscopy 2
   1.3. Relaxivity Measurements 3
   1.4. MRI Phantom Scans 3
2. Synthetic Procedures 4
3. Photophysical Properties 18
4. NMR Characterisation 19
5. Ultrasound Set-up 35
6. *In Vivo* Sonication Procedures 35
   6.1. Procedures with $Tb.L^2$ and $Lu.L^2$ 36
   6.2. Procedures with $Gd.L^2$ 36
7. *Ex Vivo* MR Imaging 37
8. Fluorescence Microscopy 37
9. Histology 40
10. References 44

1. General Procedures

All commercially available reagents were used as received from suppliers without further purification. TCO-PEG_4-NHS ester (BP-22418, BroadPharm, San Diego, CA, USA) was stored at -20 °C prior to use. Solvents used were of laboratory grade, and anhydrous solvents were obtained from departmental solvent towers and stored over 3 Å molecular sieves. Moisture-sensitive reactions were carried out under an inert atmosphere of nitrogen using Schlenk line techniques.

Reactions were monitored by thin-layer chromatography performed on silica (TLC aluminum sheets, Silica gel 60 F_{254}, Merck Supelco) and visualised under UV irradiation (254 and 366 nm), and by a liquid chromatography-mass spectrometry (LCMS) instrument (1260 Infinity II series, Agilent Technologies, Santa Clara, CA, USA) equipped with a column (InfinityLab Poroshell HPH-C18, 3.0 × 50 mm, 2.7 µm, Agilent Technologies, Santa Clara, CA, USA) and mass detector (InfinityLab LC/MSD X, Agilent Technologies, Santa Clara, CA, USA).

Automated column chromatography was performed using a Biotage® Isolera Four Flash Chromatography System and using mass dependent Biotage® Sfär Silica Duo cartridges (normal phase) and Biotage® Sfär C18 Duo 300 Å 20 µm cartridges (reversed-phase). HPLC purification was performed using a semi-preparative column (Pursuit XRs C18, 10.0 × 250 mm, 100 Å, 5.0 µm, Agilent Technologies, Santa Clara, CA, USA). Purification was performed in the Imperial College London Agilent Measurement Suite on the Agilent 1260 Infinity II Preparative LC system.

^{1}H and ^{13}C nuclear magnetic resonance (NMR) spectra were recorded on an AV-400 spectrometer at 298 K (Bruker, Billerica, MA, USA). Chemical shifts are reported in parts per million (ppm) and coupling constants in Hertz (Hz). Peak multiplicities are abbreviated as: s = singlet, d = doublet, t = triplet, m = multiplet and br = broad. Electrospray ionisation (ESI) mass spectrometry analyses were conducted by the Mass Spectrometry Service, Imperial College London.

1.1. Absorption Spectroscopy

UV-visible absorption spectra were measured using a Cary 60 Spectrophotometer (Agilent Technologies, Santa Clara, CA, USA) operating with WinUV software. The sample was held in a quartz cuvette with a path length of 1 cm. Absorption spectra were recorded against a baseline of pure solvent in an optically matched cuvette with a scan rate of 300 nm min^{-1} and a data interval of 0.5 nm. Extinction coefficients were calculated from the Beer Lambert Law (A=εcl) where A = the absorbance at a particular wavelength, ε is the extinction coefficient, c is the concentration, and l is the path length (width of the quartz cuvette, 1 cm).

1.2. Emission Spectroscopy

Fluorescence emission spectra were acquired on a Cary Eclipse Fluorescence Spectrophotometer (Agilent Technologies, Santa Clara, CA, USA), using quartz cuvettes with a path length of 1 cm. Fluorescence spectra were collected using 5 µM or 20 µM solutions in PBS buffer (pH 7.4) with the excitation and emissions slits set at 10 nm and with a scan rate of 600 nm min^{-1}.

Phosphorescence emission spectra were recorded on an Cary Eclipse Fluorescence Spectrophotometer (Agilent Technologies, Santa Clara, CA, USA) in phosphorescence mode, using quartz cuvettes with a path length of 1 cm. Phosphorescence spectra were collected using

20 µM solutions in PBS buffer (pH 7.4) with the excitation and emission slits set at 10 nm, a gate time of 0.1 ms and delay time of 0.1 ms with a data interval of 1 nm.

1.3. Relaxivity Measurements

The NMRD (Nuclear Magnetic Resonance Dispersion) profiles were measured at ^{1}H Larmor frequencies from 0.01 to 10 MHz using a 0.25 T Fast Field Cycling NMR relaxometer (SMARtracer™, Stelar, Mede, Italy), equipped with a VTC90 temperature control unit. Each point was measured 3 times and the average value was taken, with all values having percentage errors of less than 1%. The R_1 values were acquired at 25 °C and 37 °C and converted into r_1 values using the below equation, where R_1 is the observed relaxation rate, R_{1d} is the diamagnetic constant of the solvent and [CA] is the concentration of Gd^{3+} (determined using the Evans' Method).[1,2]

$$R_1 = r_1[CA]+R_{1d}$$

1.4. MRI Phantom Scans

MRI phantom scans were conducted on a preclinical 9.4 T Biospec 94/20 MRI scanner (Bruker, Billerica, MA, USA). To measure T1 relaxivity, a 2D RAREVTR (rapid acquisition with relaxation enhancement at variable TR) sequence was used: TR = 5500, 3000, 1500, 1000, 500, 250 ms, TE = 7.5 ms, RARE factor = 4, flip angle = 180, number of averages = 3, FOV = 32 mm × 32 mm × 6 mm, resolution = 0.5 mm × 0.5 mm × 1 mm. Gadolinium complexes were prepared from a stock solution in phosphate-buffered saline (PBS) to concentrations of 0 to 4 mM and a volume of 500 µL in a 3D printed mould was used for scanning.

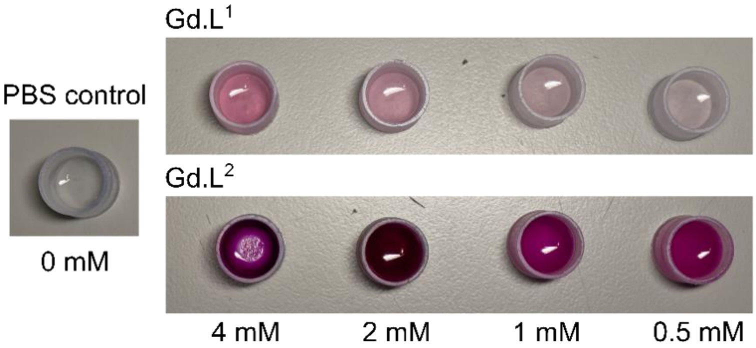

*Figure S1. 3D-printed cylindrical moulds containing solutions of the Gd complexes, **Gd.L¹** and **Gd.L²**, at different concentrations in PBS and compared with a PBS control.*

2. Synthetic Procedures

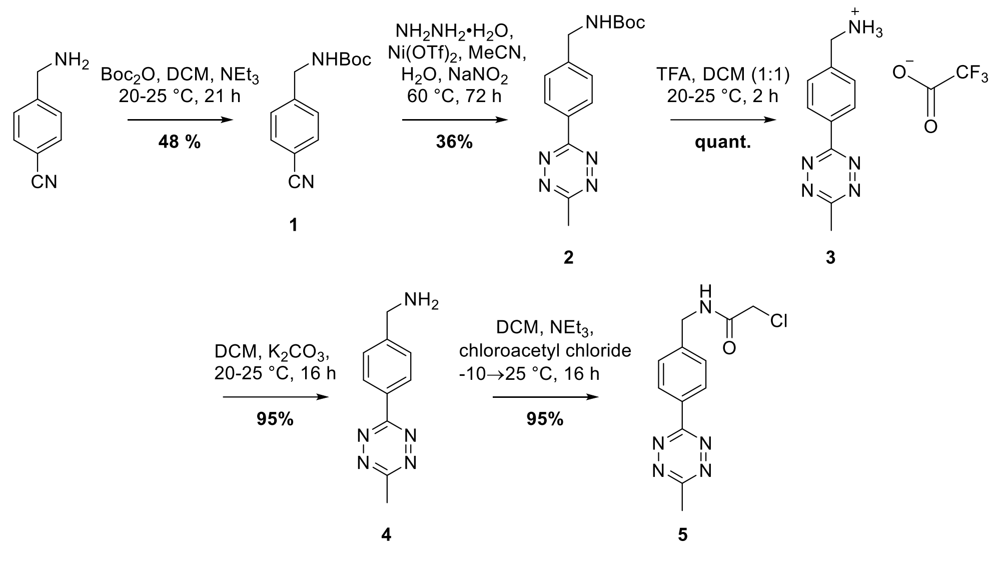

Scheme S1. Synthesis of tetrazine acyl chloride arm.

2.1. *Tert*-butyl N-(4-cyanobenzyl) carbamate (1)[3]

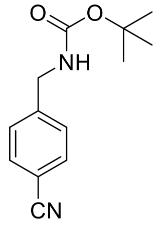

4-(Aminomethyl)benzonitrile hydrochloride (5.00 g, 30 mmol, 1.0 eq) was dissolved in CH_2Cl_2 (5 mL) and triethylamine (11 mL, 75 mmol, 2.5 eq). Di-*tert*-butyl dicarbonate (7.10 g, 33 mmol, 1.1 eq) was added and the resultant mixture was stirred at 25 °C for 21 hours. The solvent was removed under vacuum, HCl (0.5 M, 150 mL) was added and the organic phase was extracted with CH_2Cl_2 (3 × 75 mL). The combined organic extracts were washed with HCl (0.5 M, 200 mL), washed with $NaHCO_3$ solution (saturated, 200 mL), dried over $MgSO_4$ and filtered. After the removal of solvents under vacuum, **1** was obtained as a white powder (3.30 g, 14.2 mmol, 48%) and carried forward to the next step without further purification. R_f (CH_2Cl_2/MeOH, 20:1) = 0.32.

^{1}H NMR (400 MHz, $CDCl_3$) δ_H (ppm): 1.46 (s, 9H, COOC(CH_3)$_3$), 4.36 (d, $^3J_{H\text{-}H}$ = 6.1 Hz, 2H, CH_2NH), 4.98 (br. s, 1H, N*H*), 7.38 (d, $^3J_{H\text{-}H}$ = 8.1 Hz, 2H, CH_{aryl}), 7.62 (d, $^3J_{H\text{-}H}$ = 8.1 Hz, 2H, CH_{aryl}). ^{13}C NMR (101 MHz, $CDCl_3$) δ_c (ppm): 28.5 (3C, (CH_3)$_3$), 44.3 (1C), 80.2 (1C), 111.2 (1C), 118.9 (1C), 127.9 (2C), 132.5 (2C), 144.8 (1C), 156.0 (1C, *C*=O).

ESI-LRMS (ES+): $[C_{13}H_{17}N_2O_2]^+$, (+) m/z 233.1, ESI-HRMS (ES+): Anal. For $[C_{13}H_{17}N_2O_2]^+$ $[M+H]^+$ Calcd.: 233.1290, Found: 233.1277.

2.2. *Tert*-butyl (4-(6-methyl-1,2,4,5-tetrazin-3-yl)benzyl)carbamate (2)[4]

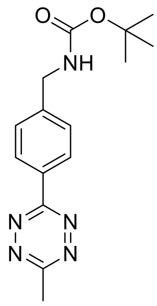

1 (0.62 g, 2.67 mmol, 0.1 eq), acetonitrile (1.4 mL, 26.8 mmol, 1.0 eq), $Ni(OTf)_2$ (0.46 g, 1.29 mmol, 0.05 eq), and hydrazine monohydrate (6.3 mL, 129 mmol, 5.0 eq) were added into a sealed high-pressure tube and stirred at 60 °C for 72 hours. After the reaction was cooled to 25 °C, H_2O (10 mL) and $NaNO_2$ (3.60 g, 52.2 mmol, 1.9 eq) were added to the mixture. HCl (1 M) was added dropwise into the brown mixture until the pH of the mixture was equal to 1. The pink solution was extracted with EtOAc (3 × 100 mL), and the combined organic layers were washed with H_2O (3 × 100 mL). The combined organic layers were dried over $MgSO_4$ and filtered. The solvent was removed under vacuum to obtain a dark pink crude product. The product was then purified by silica flash column chromatography (hexane:EtOAc, 2:1) to obtain **2** as a light pink powder (0.29 g, 0.85 mmol, 36%). R_f (Hexane/EtOAc, 2:1) = 0.47.

^{1}H NMR (400 MHz, $CDCl_3$) δ_H (ppm): 1.48 (s, 9H, COOC(C*H*$_3$)$_3$), 3.09 (s, 3H, CH_3), 4.44 (d, $^3J_{H-H}$ = 5.8 Hz, 2H, C*H*$_2$NH), 4.99 (br.s, 1H, NH), 7.50 (d, $^3J_{H-H}$ = 8.4 Hz, 2H, C*H*$_{aryl}$), 8.55 (d, $^3J_{H-H}$ = 8.4 Hz, 2H, C*H*$_{aryl}$). ^{13}C NMR (101 MHz, $CDCl_3$) δ_C (ppm): 21.3 (1C), 28.5 (3C, (*C*H$_3$)$_3$), 44.5 (1C), 80.0 (1C), 128.2 (2C), 128.3 (2C), 130.9 (1C), 144.1 (1C), 156.1 (1C, C=O), 164.1 (1C, C=N), 167.37 (1C, C=N).

ESI-LRMS (ES+): $[C_{15}H_{20}N_5O_2]^+$, (+) m/z 302.2, ESI-HRMS (ES+): Anal. For $[C_{15}H_{20}N_5O_2]^+$ $[M+H]^+$ Calcd.: 302.1617, Found: 302.1622.

2.3. (4-(6-Methyl-1,2,4,5-tetrazin-3-yl)phenyl)methanaminium-2,2,2-trifluoroacetate (3)[5]

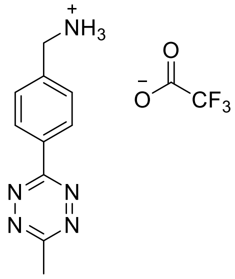

2 (0.71 g, 2.37 mmol, 1.0 eq) was dissolved in CH_2Cl_2 (11 mL) and trifluoroacetic acid (11 mL). The mixture was stirred at 25 °C for 2 hours. The mixture was reduced to dryness by co-evaporation with MeOH (3 × 11 mL) under vacuum to obtain **3** as a pink solid (0.70 g, 2.25 mmol, 95%). R_f (CH_2Cl_2/MeOH, 20:1) = 0.12.

^{1}H NMR (400 MHz, CD_3OD) δ_H (ppm): 3.06 (s, 3H, CH_3), 4.26 (s, 2H, $CH_2NH_3^+$), 7.72 (d, $^3J_{H-H}$ = 8.4 Hz, 2H, H_{aryl}), 8.64 (d, $^3J_{H-H}$ = 8.4 Hz, 2H, H_{aryl}). ^{13}C NMR (101 MHz, $CDCl_3$) δ_c (ppm): 21.1 (1C), 43.9 (1C), 129.5 (2C), 130.8 (2C), 134.3 (1C), 138.8 (1C), 164.9 (1C, C=N), 169.1 (1C, C=N). ^{19}F NMR (377 MHz, CD_3OD) δ_F (ppm): -76.90 (3F).

2.4. (4-(6-Methyl-1,2,4,5-tetrazine-3-yl)phenyl) methanamine (4)[5]

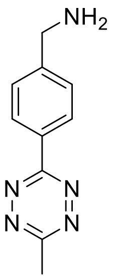

3 (0.71 g, 2.37 mmol, 1 eq) was dissolved in CH_2Cl_2 (25 mL). A solution of K_2CO_3 (saturated, 25 mL) was added and the mixture was stirred vigorously at 25 °C for 16 hours. The mixture was then extracted with CH_2Cl_2 (3 × 40 mL). The combined organic layers were dried over $MgSO_4$ and filtered, and the solvent was removed under vacuum to obtain **4** as a dark pink solid (0.45 g, 2.25 mmol, 95%). R_f (CH_2Cl_2/MeOH, 10:1) = 0.14.

^{1}H NMR (400 MHz, $CDCl_3$) δ_H (ppm): 3.09 (s, 3H, CH_3), 4.00 (s, 2H, CH_2NH_2), 7.55 (d, $^3J_{H\text{-}H}$ = 8.2 Hz, 2H, CH_{aryl}), 8.56 (d, $^3J_{H\text{-}H}$ = 8.2 Hz, 2H, CH_{aryl}). ^{13}C NMR (101 MHz, $CDCl_3$) δ_C (ppm): 21.3 (1C), 46.4 (1C), 128.0 (2C), 128.3 (2C), 130.5 (1C), 148.3 (1C), 164.2 (1C, C=N), 167.3 (1C, C=N).

ESI-LRMS (ES+): $[C_{10}H_{12}N_5]^+$, (+) *m/z* 202.1, ESI-HRMS (ES+): Anal. For $[C_{10}H_{12}N_5]^+$ $[M+H]^+$ Calcd.: 202.1093, Found: 202.1076.

UV-Vis (PBS, λ_{max}/nm): 268.

2.5. 2-Chloro-N-(4-(6-methyl-1,2,4,5-tetrazin-3-yl)benzyl)acetamide (5)[5]

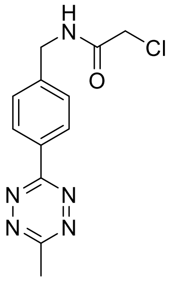

4 (0.53 g, 2.63 mmol, 1.0 eq) was dissolved in CH_2Cl_2 (75 mL). Triethylamine (0.93 mL, 6.08 mmol, 2.5 eq) was added to the solution under a nitrogen atmosphere and cooled to -10 °C. Once cooled, chloroacetyl chloride (0.25 mL, 3.16 mmol, 1.2 eq) was added into the solution. After addition was complete, the reaction mixture was allowed to warm to 25 °C and then stirred for 22 hours at 25 °C. The solvent was removed before extraction with CH_2Cl_2 (3 × 50 mL). The combined organic layers were washed with H_2O (2 × 50 mL). The organic layer was then dried over $MgSO_4$, filtered, and the solvent removed under vacuum to obtain **5** as a dark pink powder (0.69 g, 2.49 mmol, 95%). R_f (CH_2Cl_2/MeOH, 20:1) = 0.48.

^{1}H NMR (400 MHz, $CDCl_3$) δ_H (ppm): 3.11 (s, 3H, CH_3), 4.16 (s, 2H, CH_2Cl), 4.63 (d, $^3J_{H\text{-}H}$ = 6.1 Hz, 2H, CH_2NH), 7.00 (br.s, 1H, NH), 7.52 (d, $^3J_{H\text{-}H}$ = 8.4 Hz, 2H, CH_{aryl}), 8.59 (d, $^3J_{H\text{-}H}$ = 8.4 Hz, 2H, CH_{aryl}). ^{13}C NMR (101 MHz, $CDCl_3$) δ_C (ppm): 21.3 (1C), 42.8 (1C), 43.6 (1C), 128.5 (2C), 128.6 (2C), 131.4 (1C), 142.2 (1C), 163.9 (1C, C=N), 166.2 (1C, C=N), 167.5 (1C, C=O).

ESI-LRMS (ES+): $[C_{12}H_{13}N_5OCl]^+$, (+) *m/z* 278.1, ESI-HRMS (ES+): Anal. For $[C_{12}H_{13}N_5OCl]^+$ $[M+H]^+$ Calcd.: 278.0809, Found: 278.0790.

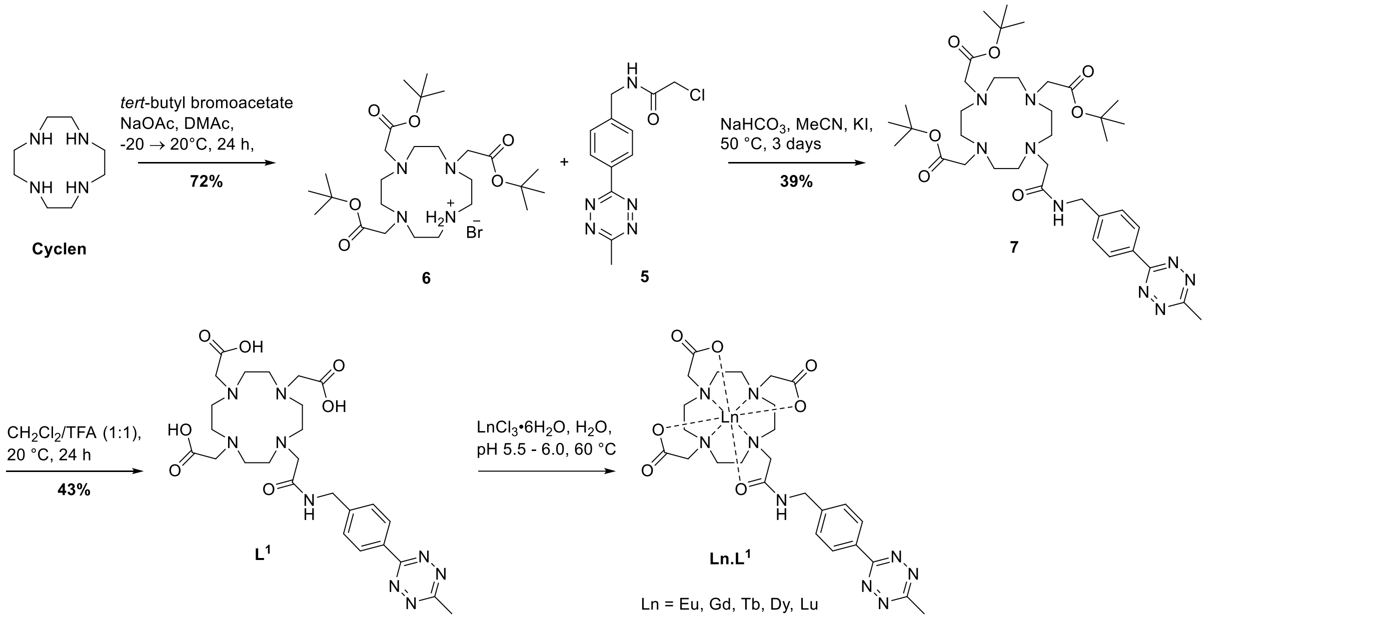

Scheme S2. Synthesis of Ln(DO3A-tetrazine), ***Ln.L¹***.

2.6. *Tert*-butyl-DO3A.HBr (6)[6]

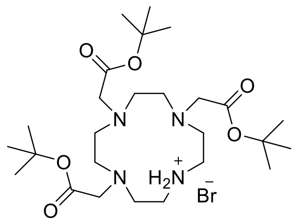

1,4,7,10-Tetraazacyclododecane (Cyclen) (5.06 g, 29.4 mmol, 1.0 eq) and sodium acetate (8.02 g, 97.8 mmol, 3.3 eq) were dissolved in *N,N*-dimethylacetamide (60 mL). The mixture was cooled to -20 °C, and a solution of *tert*-butyl bromoacetate (14.5 mL, 97.9 mmol, 3.4 eq) in *N,N*-dimethylacetamide (20 mL) was added dropwise into the mixture over 30 minutes. The mixture was allowed to warm to 25 °C and stirred for 24 hours at 25 °C. The mixture was poured into H_2O (300 mL), and $KHCO_3$ (15.2 g, 152.8 mmol, 5.2 eq) was added portion-wise (3 × 100 mL). The white precipitation formed was isolated by filtration. The white solid was redissolved in $CHCl_3$ (190 mL), washed with H_2O (100 mL) and dried over $MgSO_4$. The solution was concentrated under vacuum, and diethyl ether (250 mL) was added to form a white precipitate. After filtration, the product **6** (12.6 g, 21.1 mmol, 72%) was obtained as a white powder.

^1H NMR (400 MHz, $CDCl_3$) δ_H (ppm): 1.45 (s, 9H, COOC(CH_3)$_3$), 1.46 (s, 18H, COOC(CH_3)$_3$), 2.92 (m, 12H, CH_2), 3.10 (m, 4H, CH_2), 3.28 (s, 2H, CH_2), 3.37 (s, 2H, CH_2), 10.04 (br.s, 2H, NH_2). ^{13}C NMR (101 MHz, $CDCl_3$) δ_C (ppm): 28.3 (6C, (CH_3)$_3$), 28.4 (3C, (CH_3)$_3$), 47.7 (2C), 48.8 (1C), 49.3 (2C), 51.3 (1C), 51.5 (2C), 58.4 (3C) , 81.8 (2C, C-O), 82.0 (1C, C-O), 169.8 (1C, C=O), 170.7 (2C, C=O).

ESI-LRMS (ES+): $[C_{26}H_{51}N_4O_6]^+$, (+) *m/z* 515.4, ESI-HRMS (ES+): Anal. For $[C_{26}H_{51}N_4O_6]^+$ $[M+H]^+$ Calcd.: 515.3809, Found: 515.3801.

2.7. *Tert*-butyl-DO3A-tetrazine (7)[5]

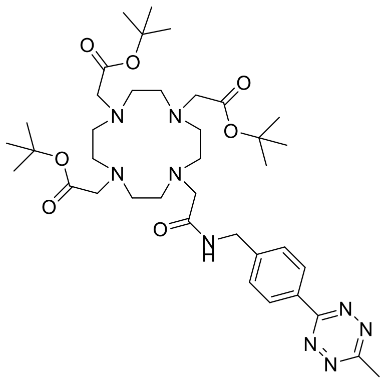

5 (0.69 g, 2.49 mmol, 1.05 eq), **6** (1.41 g, 2.37 mmol, 1 eq), $NaHCO_3$ (1.39 g, 16.6 mmol, 7 eq), and potassium iodide (39 mg, 0.24 mmol, 0.1 eq) were dissolved in acetonitrile (30 mL) and stirred at 50 °C for 3 days. The mixture was cooled and the inorganic salts were filtered. The solvents were removed under vacuum to obtain a pink solid. The crude product was purified by silica flash column chromatography (CH_2Cl_2/MeOH: 0% to 20%) to obtain **7** as a dark pink solid (0.70 g, 0.93 mmol, 39%). R_f (CH_2Cl_2/MeOH, 2:1) = 0.33.

^1H NMR (400 MHz, $CDCl_3$) δ_H (ppm): 1.39 (s, 18H, COOC(CH_3)$_3$), 1.44 (s, 9H, COOC(CH_3)$_3$), 1.83-2.96 (br, 16H, CH_2), 3.07 (s, 3H, CH_3), 3.48 (s, 6H, CH_2), 3.55 (s, 2H, CH_2), 4.52 (s, 2H, CH_2NH), 7.60 (d, $^3J_{H-H}$ = 8.4 Hz, 2H, CH_{aryl}), 7.60 (d, $^3J_{H-H}$ = 8.45 Hz, 2H, CH_{aryl}).^{13}C NMR (101 MHz, $CDCl_3$) δ_C (ppm): 21.2 (1C), 27.9 (3C, (CH_3)$_3$), 28.0 (3C, (CH_3)$_3$), 28.3 (3C, (CH_3)$_3$), 42.6 (1C), 47.6-53.9 (8C), 55.7 (1C), 55.8 (2C), 56.4 (1C), 81.7 (1C), 82.0 (2C), 127.9 (2C), 128.8 (2C), 129.9 (1C), 145.2 (1C), 164.2 (1C, C=N), 167.0 (1C, C=N), 170.6 (1C, C=O), 172.2 (2C, C=O), 172.4 (1C, C=O).

ESI-LRMS (ES+): $[C_{38}H_{62}N_9O_7]^+$, (+) *m/z* 756.5, ESI-HRMS (ES+): Anal. For $[C_{38}H_{62}N_9O_7]^+$ $[M+H]^+$ Calcd.: 756.4772, Found: 756.4749.

2.8. DO3A-tetrazine (L^1)[5]

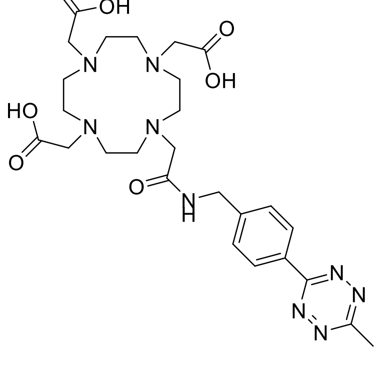

7 (0.63 g, 0.84 mmol, 1.0 eq) was dissolved in CH_2Cl_2 (26 mL) and trifluoroacetic acid (13.6 mL, excess) and the reaction mixture was stirred at 25 °C for 44 hours. The mixture was reduced to dryness by co-evaporation with MeOH (5 × 30 mL) to obtain a red oil. The red oil was triturated in diethyl ether (45 mL) and then centrifuged to obtain the crude product as a pink solid. Following purification by reverse phase column chromatography (H_2O + 0.1% trifluoroacetic acid/acetonitrile + 0.1% trifluoroacetic acid: 0% to 95%) and lyophilisation, **L¹** was obtained as a light pink solid (0.21 g, 0.36 mmol, 43%).

^{1}H NMR (400 MHz, D_2O) δ_H (ppm): 3.01 (s, 3H, CH_3), 3.08-3.53 (br.m, 16H, CH_2), 3.57-4.30 (br.m, 8H, CH_2), 4.49 (s, 2H, CH_2NH), 7.52 (d, $^3J_{H\text{-}H}$ = 8.1 Hz, 2H, CH_{aryl}), 8.28 (d, $^3J_{H\text{-}H}$ = 8.1 Hz, 2H, CH_{aryl}). ^{13}C NMR (101 MHz, D_2O) δ_C (ppm): 20.0 (1C), 42.9 (1C), 50.1 (8C), 53.7 (1C), 55.0 (3C), 111.9 (1C), 114.8 (2C), 117.7 (2C), 120.6 (1C), 128.1 (2C, C=O), 128.2 (1C, C=O), 130.2 (1C, C=O), 163.8 (1C, C=N), 167.2 (1C, C=N).

ESI-LRMS (ES+): $[C_{26}H_{38}N_9O_7]^+$, (+) *m/z* 588.3, ESI-HRMS (ES+): Anal. For $[C_{26}H_{38}N_9O_7]^+$ $[M+H]^+$ Calcd.: 588.2894, Found: 588.2899.

2.9. General Procedures for complexation of Ln.L¹

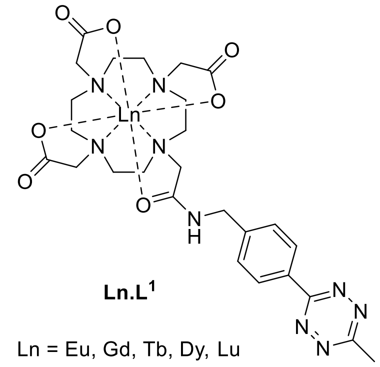

Ln.L¹

Ln = Eu, Gd, Tb, Dy, Lu

L¹ (1.0 eq) and $LnCl_3{\cdot}6H_2O$ (1.1-1.6 eq) were dissolved in water (2-5 mL). The pH of the mixture was adjusted to 5.5-5.9 by the dropwise addition of 0.1 M NaOH. The mixture was then stirred at 60 °C for 7 hours. The reaction was cooled to 25 °C and the solvent was removed under vacuum to obtain a pink crude solid. Following purification by reverse phase column chromatography (H_2O/acetonitrile: 0% to 95%, neutral) and lyophilisation, **Ln.L¹** was obtained as a pink solid (60%-quantitative yield).

2.9.1. Eu(DO3A-tetrazine) (Eu.L¹)[5]

Eu.L¹ was prepared as previously described.[6] **L¹** (30.5 mg, 0.05 mmol, 1.0 eq) and $EuCl_3{\cdot}6H_2O$ (22.0 mg, 0.06 mmol, 1.2 eq) were dissolved in water (5 mL). **Eu.L¹** was obtained as a light pink solid (22.1 mg, 0.03 mmol, 60%).

^{1}H NMR (400 MHz, D_2O) δ_H (ppm) 33.3, 32.4, 32.0, 31.8, 12.0, -0.1, -0.4, -2.3, -2.9, -3.0, -3.9, -5.1, -6.2, -6.9, -7.0, -7.3, -7.8, -7.9, -10.8, -11.0, -11.3, -12.5, -12.8, -14.9, -15.2, -15.5, -15.9, -16.1, -16.9, -17.2 (only peaks outside of 0 to 10 ppm reported).

ESI-LRMS (ES+): $[C_{26}H_{35}N_9O_7Eu]^+$ (+) *m/z* 736.2, ESI-HRMS (ES+): Anal. For $[C_{26}H_{36}N_9O_7Eu]^+$ $[M+H]^+$ Calcd.: 736.1858, Found: 736.1840.

UV-Vis (PBS, λ_{max}/nm): 264.

2.9.2. Gd(DO3A-tetrazine) (Gd.L^1)[5]

L^1 (19.8 mg, 0.03 mmol, 1.0 eq) and $GdCl_3{\cdot}6H_2O$ (18.9 mg, 0.05 mmol, 1.6 eq) were dissolved in water (2 mL). **Gd.L^1** was obtained as a light pink solid (0.021 g, 0.03 mmol, quantitative yield).

ESI-LRMS (ES+): $[C_{26}H_{35}N_9O_7Gd]^+$ (+) *m/z* 743.2, ESI-HRMS (ES+): Anal. For $[C_{26}H_{35}N_9O_7Gd]^+$ $[M+H]^+$ Calcd.: 743.1901, Found: 743.1937.

UV-Vis (PBS, λ_{max}/nm): 265.

2.9.3. Tb(DO3A-tetrazine) (Tb.L^1)[5]

L^1 (30.7 mg, 0.05 mmol, 1.0 eq) and $TbCl_3{\cdot}6H_2O$ (18.0 mg, 0.06 mmol, 1.2 eq) were dissolved in water (3 mL). **Tb.L^1** was obtained as a light pink solid (43.0 mg, 0.06 mmol, quantitative yield).

^{1}H NMR (400 MHz, D_2O) δ_H (ppm) 257.1, 247.1, 242.4, 202.1, 192.5, 108.8, 74.9, 65.2, 62.6, 47.9, 28.0, 20.6, 18.3, 10.6, -39.8, -44.8, -58.8, -63.7, -71.3, -76.4, -77.8, -79.0, -93.7, -105.2, -111.1, -114.0, -122.2, -202.8, -372.2, -378.3, -382.2, -394.7 (only peaks outside of -10 to 10 ppm reported).

ESI-LRMS (ES+): $[C_{26}H_{34}N_9O_7Tb]^+$ (+) *m/z* 744.2, ESI-HRMS (ES+): Anal. For $[C_{26}H_{35}N_9O_7Tb]^+$ $[M+H]^+$ Calcd.: 744.1913, Found: 744.1937.

UV-Vis (PBS, λ_{max}/nm): 265.

2.9.4. Dy(DO3A-tetrazine) (Dy.L^1)

L^1 (50.0 mg, 0.09 mmol, 1.0 eq) and $DyCl_3{\cdot}6H_2O$ (37.4 mg, 0.10 mmol, 1.1 eq) were dissolved in water (5 mL). **Dy.L^1** was obtained as a light pink solid (44.9 mg, 0.06 mmol, 67%).

^{1}H NMR (500 MHz, D_2O) δ_H (ppm) 315.7, 290.2, 282.3, 181.1, 176.5, 51.2, 44.0, 32.5, 28.7, 15.1, 13.5, -24.3, -35.4, -50.2, -58.5, -65.9, -80.7, -107.8, -132.8, -136.5, -168.8, -432.1, -441.3, -453.0, -495.1 (only peaks outside of -10 to10 ppm reported).

ESI-LRMS (ES+): $[C_{26}H_{35}N_9O_7Dy]^+$ (+) *m/z* 749.2, ESI-HRMS (ES+): Anal. For $[C_{26}H_{35}N_9O_7Dy]^+$ $[M+H]^+$ Calcd.: 749.1951, Found: 749.1973.

UV-Vis (PBS, λ_{max}/nm): 265.

2.9.5. Lu(DO3A-tetrazine) (Lu.L[1])

L[1] (30.6 mg, 0.05 mmol, 1.0 eq) and $LuCl_3 \cdot 6H_2O$ (22.0 mg, 0.06 mmol, 1.1 eq) were dissolved in water (3 mL). **Lu.L[1]** was obtained as a light pink solid (24.6 mg, 0.03 mmol, 65%).

^{1}H NMR (400 MHz, D_2O) δ_H (ppm):1.96-2.99 (br. m, 16H, CH_2), 3.10 (s, 3H, CH_3), 3.16-3.98 (br.m, 8H, CH_2), 4.47 (d, $^3J_{H-H}$ = 15.2 Hz, 1H, CH_2NH), 4.69(d, $^3J_{H-H}$ = 15.2 Hz, 1H, CH_2NH), 7.67 (d, $^3J_{H-H}$ = 8.1 Hz, 2H, CH_{aryl}), 8.39 (d, $^3J_{H-H}$ = 8.1 Hz, 2H, CH_{aryl}). ^{13}C NMR (101 MHz, D_2O) δ_C (ppm): 20.1 (1C), 43.8 (1C), 54.5 (2C), 55.2 (4C), 55.7 (2C), 63.2 (1C), 64.7 (2C), 65.6 (1C), 128.5 (2C), 128.8 (2C), 130.8 (1C), 142.7 (1C), 163.9 (1C, C=N), 167.3 (1C, C=N), 176.3 (1C, C=O), 180.1 (1C, C=O), 180.8 (2C, C=O).

ESI-LRMS (ES+): $[C_{26}H_{35}N_9O_7Lu]^+$ (+) *m/z* 760.2, ESI-HRMS (ES+): Anal. For $[C_{26}H_{35}N_9O_7Lu]^+$ $[M+H]^+$ Calcd.: 760.2050, Found: 760.2067.

UV-Vis (PBS, λ_{max}/nm): 265.

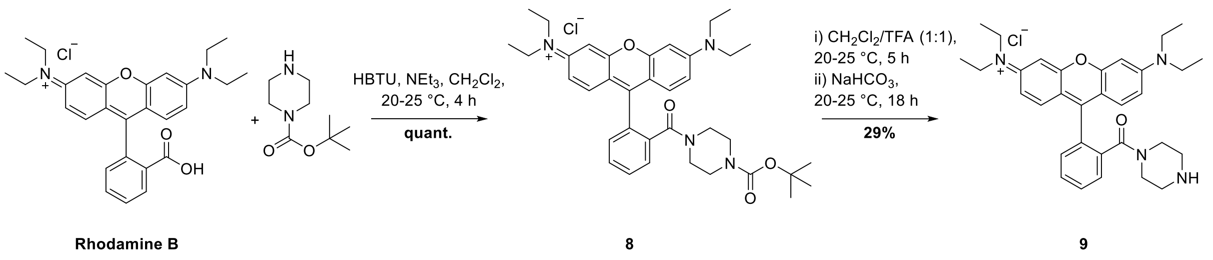

*Scheme S3. Synthesis of rhodamine-piperazine, **9**.*

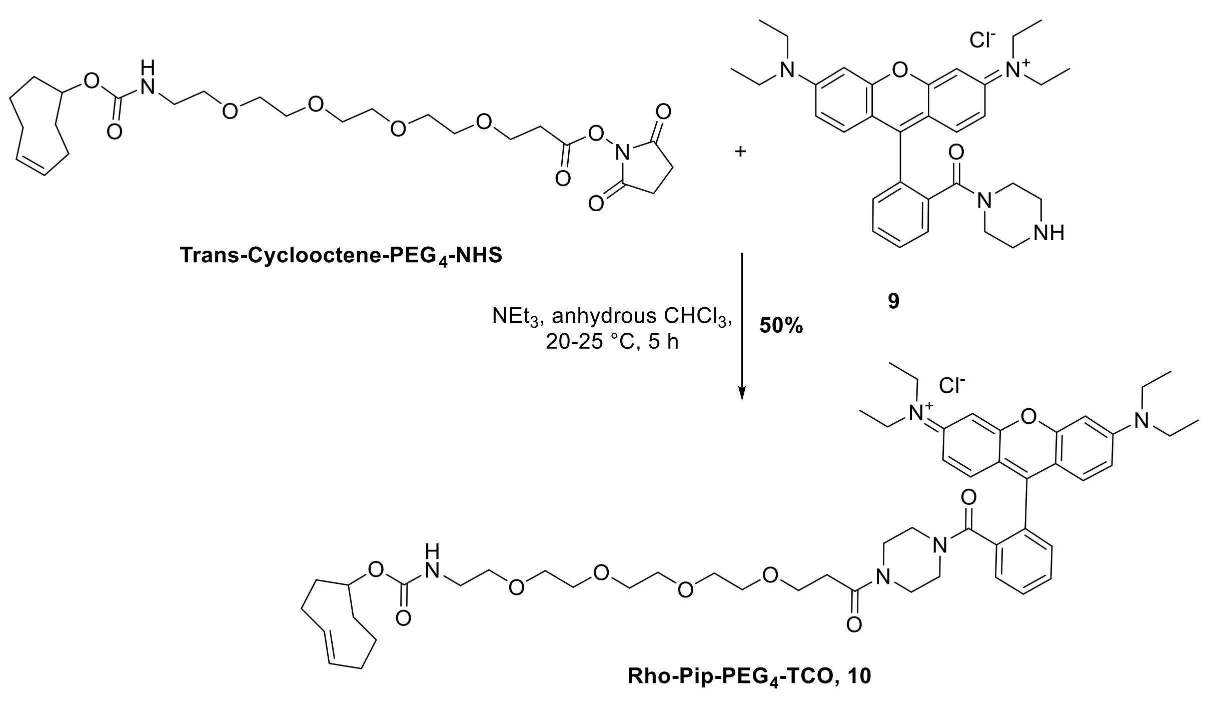

*Scheme S4. Synthesis of Rho-Pip-PEG$_4$-TCO, **10**.*

2.10. Boc-rhodamine-piperazine (8)[7]

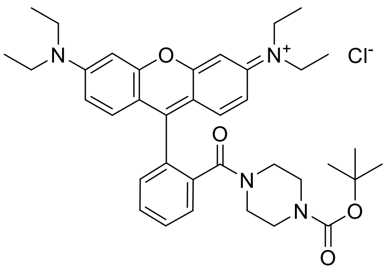

Rhodamine B (1.02 g, 2.09 mmol, 1.0 eq), 1-boc-piperazine (0.40 g, 2.09 mmol, 1.0 eq) and triethylamine (0.8 mL, 5.22 mmol, 2.5 eq) were dissolved in CH_2Cl_2 (30 mL). HBTU was added, and the mixture was stirred at 25 °C for 4 hours. The solution was then diluted in CH_2Cl_2 (30 mL). The organic layer was washed with brine (2 × 30 mL), dried over Na_2SO_4, filtered and the solvents were removed under vacuum. The crude product **8** (1.88 g, 2.89 mmol, quantitative yield) was obtained as a purple solid and carried forward to the next step without further purification.

^{1}H NMR (400 MHz, $CDCl_3$) δ_H (ppm): 1.32 (t, $^3J_{H-H}$ = 7.2 Hz, 12H, CH_3), 1.43 (s, 9H, COOC(CH_3)$_3$), 3.24-3.31 (m, 4H, CH_2), 3.34-3.42 (m, 4H, CH_2), 3.61 (sept, $^3J_{H-H}$ = 7.2 Hz, 8H, CH_2), 6.71-6.84 (m, 2H, CH_{aryl}), 6.92-6.99 (m, 4H, CH_{aryl}), 7.33-7.38 (m, 1H, CH_{aryl}), 7.49-7.54 (m, 1H, CH_{aryl}), 7.65-7.69 (m, 2H, CH_{aryl}). ^{13}C NMR (101 MHz, $CDCl_3$) δ_C (ppm): 12.7 (4C), 28.4 (3C, (CH_3)$_3$), 38.7 (4C), 46.2 (4C, C-N), 80.7 (1C, C-O), 96.4 (2C), 113.9 (1C), 114.2 (1C), 127.6 (2C), 130.2 (2C), 130.5 (2C), 130.9 (1C), 132.2 (2C), 135.2 (1C), 147.8 (1C), 155.8 (2C, C=N, C-N), 157.9 (2C, C-O), 165.9 (1C, C=O), 167.8 (1C, C=O).

ESI-LRMS (ES+): $[C_{37}H_{47}N_4O_4]^+$, (+) *m/z* 611.4, ESI-HRMS (ES+): Anal. For $[C_{37}H_{47}N_4O_4]^+$ $[M]^+$ Calcd.: 611.3597, Found: 611.3600.

2.11. Rhodamine-piperazine (9)[7]

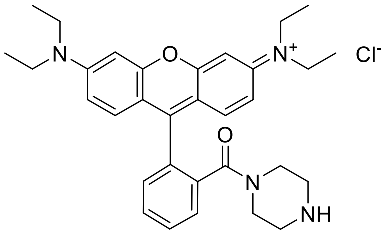

8 (1.75 g, 2.7 mmol, 1.0 eq) was dissolved in CH_2Cl_2 (70 mL) and cooled to 0 °C. Trifluoroacetic acid (30 mL, excess) was added and the mixture was stirred at 25 °C for 5 hours. The reaction was reduced to dryness by co-evaporation with CH_2Cl_2 (5 × 30 mL) and the resulting purple solid was redissolved in CH_2Cl_2 (70 mL). A solution of $NaHCO_3$ (saturated, 70 mL) was added and the reaction mixture was stirred vigorously at 25 °C overnight. The organic layer was separated and the remaining aqueous layer was extracted with CH_2Cl_2 (2 × 30 mL). The combined organic layers were washed with brine (2 × 90 mL), dried over $MgSO_4$, filtered and the solvent was removed under reduced pressure. The crude product was purified by silica flash column chromatography (CH_2Cl_2/MeOH: 5% to 20%) to obtain **9** as a brown solid (0.40 g, 0.79 mmol, 29%).

^{1}H NMR (400 MHz, $CDCl_3$) δ_H (ppm): 1.32 (t, $^{3}J_{H-H}$ = 7.6 Hz, 12H, CH_3), 2.74-3.06 (m, 4H, CH_2), 3.59 (septet, $^{3}J_{HH}$ = 7.6 Hz, 8H, CH_2), 3.69-3.80 (m, 4H, CH_2), 6.60-6.76 (m, 2H, CH_{aryl}), 6.84-7.02 (m, 2H, CH_{aryl}), 7.15-7.21 (m, 2H, CH_{aryl}), 7.31-7.39 (m, 1H, CH_{aryl}), 7.49-7.58 (m, 1H, CH_{aryl}), 7.62-7.70 (m, 2H, CH_{aryl}). ^{13}C NMR (101 MHz, $CDCl_3$) δ_C (ppm): 12.8 (4C), 40.1 (1C, C-N), 44.0 (1C, C-N), 44.5 (1C, C-N), 45.9 (1C, C-N), 46.4 (4C), 96.4 (2C), 113.7 (1C), 114.4 (2C), 127.8 (2C), 130.1 (1C), 130.3 (2C), 130.5 (1C), 131.0 (1C), 131.9 (2C), 135.11 (1C), 155.8 (2C, C-N, C=N), 157.8 (2C, C-O), 167.3 (1C, C=O).

ESI-LRMS (ES+): $[C_{32}H_{39}N_4O_2]^+$ (+) *m/z* 511.3, ESI-HRMS (ES+): Anal. For $[C_{32}H_{39}N_4O_2]^+$ $[M]^+$ Calcd.: 511.3073, Found: 511.3075.

UV-Vis (PBS, λ_{max}/nm): 566.

2.12. Rho-Pip-PEG$_4$-TCO (10)

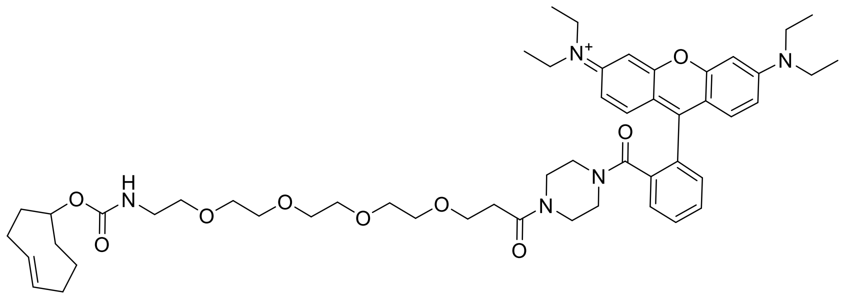

9 (40.7 mg, 0.078 mmol, 1.0 eq) was dissolved in $CHCl_3$ (0.2 mL) and triethylamine (0.22 mL, 1.5 mmol, excess) under anhydrous conditions. A solution of TCO-PEG$_4$-NHS (42.2 mg, 0.082 mmol, 1.05 eq) in anhydrous $CHCl_3$ (2 mL) was added and the mixture was stirred at 25 °C for 5 hours under a nitrogen atmosphere, with the reaction monitored to completion by ^{1}H NMR. The solvent was removed under vacuum. The crude product was purified by column chromatography (CH_2Cl_2 /MeOH: 0% to 20%) to obtain **10** as a purple solid (51.0 mg, 0.056 mmol, 72%).

^{1}H NMR (400 MHz, D_2O) δ_H (ppm): 1.12-1.21 (m, 4H, CH_2), 1.26 (t, $^{3}J_{H-H}$ = 7.3 Hz, 12H, CH_3), 1.40-1.58 (m, 2H, CH_2), 1.68-1.84 (m, 2H, CH_2), 2.02-2.18 (m, 2H, CH_2), 2.50-2.65 (m, 2H, CH_2), 2.68-2.76 (m, 2H, CH_2), 3.17 (q, $^{3}J_{H-H}$ = 7.3 Hz, 8H, CH_2), 3.24-3.76 (m, 24H, CH_2), 4.03-4.19 (m, 1H, CH), 5.20-5.52 (m, 2H, CH_{alkene}), 6.48-6.68 (m, 2H, CH_{aryl}), 6.90 (d, $^{3}J_{H-H}$ = 9.4 Hz, 2H, CH_{aryl}), 7.16 (d, $^{3}J_{H-H}$ = 9.4 Hz, 2H, CH_{aryl}), 7.34-7.47 (m, 1H, CH_{aryl}), 7.59-7.70 (m, 2H, CH_{aryl}), 7.71-7.84 (m, 2H, CH_{aryl}).

ESI-LRMS (ES+): $[C_{52}H_{72}N_5O_9]^+$, (+) *m/z* 910.5, ESI-HRMS (ES+): Anal. For $[C_{52}H_{72}N_5O_9]^+$ $[M]^+$ Calcd.: 910.5330, Found: 910.5366.

UV-Vis (PBS, λ_{max}/nm): 566.

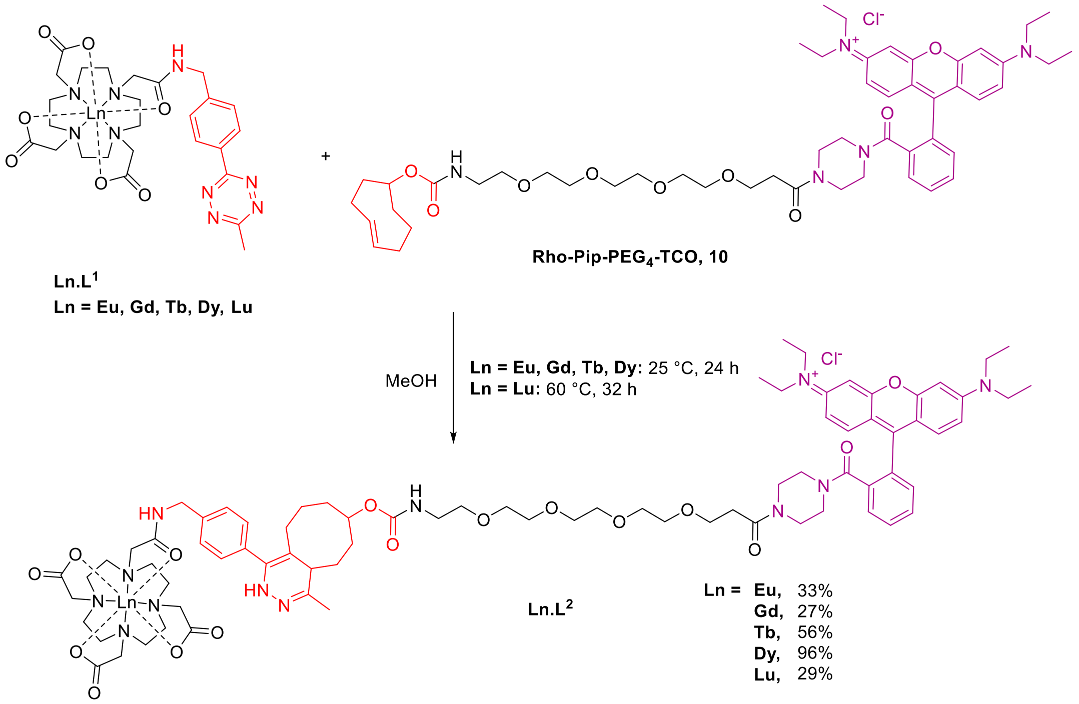

*Scheme S5. IEDDA 'click' reaction between Ln(DO3A-tetrazine), **Ln.L¹**, and Rho-Pip-PEG₄-TCO, **10**, to synthesise the click conjugates, **Ln.L²**.*

2.13. General Procedures for L² and Ln.L²

L¹ or **Ln.L¹** (1.0 eq) and **10** (1.0-1.5 eq) were dissolved in H_2O, MeOH or a mixture of H_2O and MeOH. The solution was stirred at 25 °C for 24-32 hours. The solvent was removed under vacuum. The crude product was dissolved in 3:1 H_2O:MeCN and purified by HPLC using prep HPLC gradient methods 1 or 2 (**Error! Reference source not found.**-S2). After lyophilisation, **L²** or **Ln.L²** was obtained as a dark purple solid (25%-96%).

Table S1. ***Prep HPLC gradient method 1****, A: H_2O + 0.1% formic acid; B: MeCN + 0.1% formic acid, collect from λ = 254 nm.*

Time (min)	A%	B/%	Flow rate (mL/min)
0.00	95.0	5.0	10.0
0.10	95.0	5.0	10.0
17.50	5.0	95.0	10.0
18.50	5.0	95.0	10.0
19.00	95.0	5.0	10.0
20.00	95.0	5.0	10.0

Table S2. ***Prep HPLC gradient method 2****, A: H_2O + 0.1% formic acid; B: MeCN + 0.1% formic acid, collect from λ = 254 nm.*

Time (min)	A/%	B/%	Flow rate (mL/min)
0.00	95.0	5.0	4.0
0.10	95.0	5.0	4.0
20.00	30.0	70.0	4.0
20.50	5.0	95.0	4.0
21.50	5.0	95.0	4.0
22.00	95.0	5.0	4.0
24.00	95.0	5.0	4.0

2.13.1. DO3A-PEG$_4$-piperazine-rhodamine (L^2)

L^1 (7.2 mg, 12.1 μmol, 1.1 eq) and **10** (9.8 mg, 11.0 μmol, 1 eq) were dissolved in H_2O (1.25 mL). The solution was stirred at 60 °C for 7 hours. The solvent was removed under vacuum. The crude product was purified by HPLC using prep HPLC gradient method 2 (Table S2). After lyophilisation, **L^1** was obtained as a dark purple solid (2.9 mg, 2.0 μmol, 18%).

ESI-LRMS (ES+): $[C_{78}H_{104}N_{12}O_{16}]^{2+}$, (2+) *m/z* 734.9, ESI-HRMS (ES+): Anal. For $[C_{78}H_{104}N_{12}O_{16}]^{2+}$ $[M+2H]^{2+}$ Calcd.: 734.9037, Found: 735.3635.

UV-Vis (PBS, λ_{max}/nm): 569.

t_R (prep) = 12.71-12.80 min.

2.13.2. Eu(DO3A-PEG$_4$-piperazine-rhodamine) (Eu.L^2)

Eu.L^1 (10.0 mg, 11.0 µmol, 1.0 eq) and **10** (12.2 mg, 17.0 µmol, 1.5 eq) were dissolved in MeOH (0.5 mL). The crude product was dissolved in 3:1 H_2O:MeCN and purified by HPLC using prep HPLC gradient method 1 (Table S1) to obtain **Eu.L^2** as a dark pink solid (5.8 mg, 3.6 µmol, 33%).

^{1}H NMR (400 MHz, CD_3OD) δ_H (ppm) 38.9, 36.4, 35.6, 34.4, 16.3, 14.1, -1.4, -1.8, -2.4, -3.6, -5.2, -6.4, -6.5, -9.4, -11.2, -12.7, -13.8, -14.3, -17.2, -18.1, -18.2, -18.7, -18.9 (only peaks outside of 0 to 10 ppm reported).

ESI-LRMS (ES+): $[C_{78}H_{104}N_{12}O_{16}Eu]^+$, (+) *m/z* 1617.7, ESI-HRMS (ES+): Anal. For $[C_{78}H_{104}N_{12}O_{16}Eu]^+$ $[M+H]^+$ Calcd.: 1617.6906, Found: 1617.6907.

UV-Vis (PBS, λ_{max}/nm): 569.

t_R (prep) = 8.26 min.

2.13.3. Gd(DO3A-PEG$_4$-piperazine-rhodamine) (Gd.L^2)

Gd.L^1 (12.1 mg, 16.0 µmol, 1.0 eq) and **10** (15.0 mg, 16 µmol, 1.0 eq) were dissolved in H_2O:MeOH (2 mL). The crude product was dissolved in 3:1 H_2O:MeCN and purified by HPLC using prep HPLC gradient method 1 (Table S1) to obtain **Gd.L^2** as a dark pink solid (7 mg, 4.0 µmol, 25%).

ESI-LRMS (ES+): $[C_{78}H_{104}N_{12}O_{16}Gd]^{2+}$, (2+) *m/z* 811.9, ESI-HRMS (ES+): Anal. For $[C_{78}H_{104}N_{12}O_{16}Gd]^{2+}$ $[M+2H]^{2+}$ Calcd.: 811.8501, Found: 811.8503.

UV-Vis (PBS, λ_{max}/nm): 569.

t_R (prep) = 8.11-8.21 min.

2.13.4. Tb(DO3A-PEG$_4$-piperazine-rhodamine) (Tb.L^2)

Tb.L^1 (12.2 mg, 16.0 µmol, 1.0 eq) and **10** (10.0 mg, 11.0 µmol, 1.2 eq) were dissolved in MeOH (0.5 mL). The crude product was dissolved in 3:1 H_2O:MeCN and purified by HPLC using prep HPLC gradient method 1 (Table S1) to obtain **Tb.L^2** as a dark pink solid (14.0 mg, 9.0 µmol, 56%).

^{1}H NMR (400 MHz, CD_3OD) δ_H (ppm) 277.0, 270.1, 255.8, 236.8, 230.3, 135.3, 128.2, 51.5, 45.8, 34.6, 30.3, 22.2, 15.6, 13.7, 13.6, 13.4, 13.0, 12.6, 12.3, 11.7, 11.1, 10.9, -25.2, -32.3, -40.4, -52.1, -61.4, -70.3, -73.3, -80.8, -104.5, -118.1, -125.5, -130.7, -148.4, -221.7, -226.3, -246.5, -259.7, -384.6, -389.5, -396.2, -428.3 (only peaks outside of -10 to 10 ppm reported).

ESI-LRMS (ES+): $[C_{78}H_{104}N_{12}O_{16}Tb]^{2+}$, (2+) *m/z* 812.4, ESI-HRMS (ES+): Anal. For $[C_{78}H_{104}N_{12}O_{16}Tb]^{2+}$ $[M+2H]^{2+}$ Calcd.: 812.3513, Found: 812.3514.

UV-Vis (PBS, λ_{max}/nm): 569.

t_R (prep) = 8.20-8.24 min.

2.13.5. Dy(DO3A-PEG$_4$-piperazine-rhodamine) (Dy.L^2)

Dy.L^1 (13.4 mg, 18.0 μmol, 1.0 eq) and **10** (16.1 mg, 18.0 μmol, 1.0 eq) were dissolved in MeOH (0.5 mL). The crude product was dissolved in 3:1 H_2O:MeCN and purified by HPLC using prep HPLC gradient method 1 (Table S1) to obtain **Dy.L^2** as a dark pink solid (28.4 mg, 17.4 μmol, 96%).

^{1}H NMR (500 MHz, CD_3OD) δ_H (ppm) 367.9, 347.1, 325.8, 238.0, 226.7, 25.5, -13.9, -14.3, -29.2, -38.1, -43.9, -51.8, -53.4, -60.7, -80.8, -97.3, -100.0, -117.0, -133.8, -159.3, -167.3, -193.4, -221.0 (only peaks outside of -10 to 10 ppm reported).

ESI-LRMS (ES+): $[C_{78}H_{104}N_{12}O_{16}Dy]^+$, (+) *m/z* 1628.7, ESI-HRMS (ES+): Anal. For $[C_{78}H_{104}N_{12}O_{16}Dy]^+$ $[M+H]^+$ Calcd.: 1628.6985, Found: 1628.6960.

UV-Vis (PBS, λ_{max}/nm): 569.

t_R (prep) = 8.17-8.30 min.

2.13.6. Lu(DO3A-PEG$_4$-piperazine-rhodamine) (Lu.L^2)

Lu.L^1 (7.2 mg, 9.0 μmol, 1.0 eq) and **10** (8.0 mg, 9.0 μmol, 1.0 eq) were dissolved in MeOH (5 mL). The crude product was dissolved in 3:1 H_2O:MeCN and purified by HPLC using prep HPLC gradient method 2 (Table S2) to obtain **Lu.L^2** as a dark pink solid (4.0 mg, 2.4 μmol, 29%).

ESI-LRMS (ES+): $[C_{78}H_{104}N_{12}O_{16}Lu]^{2+}$, (2+) *m/z* 820.4, ESI-HRMS (ES+): Anal. For $[C_{78}H_{104}N_{12}O_{16}Lu]^+$ $[M+H]^+$ Calcd.: 1639.7101, Found: 1639.7040.

UV-Vis (PBS, λ_{max}/nm): 569.

t_R (prep) = 12.70-12.80 min.

3. Photophysical Properties

Table S3. UV-vis absorbance, molar extinction coefficients and quantum yields for the compounds.

Compound	λ_{abs} (nm)	ε (M^{-1} cm^{-1})	ϕ
**DO3A-tetrazine, L[1]**	265	19 000	<0.01
**Eu.L[1]**	265	19 000	<0.01
**Gd.L[1]**	268	22 000	<0.01
**Tb.L[1]**	268	11 000	<0.01
**Dy.L[1]**	268	18 000	<0.01
**Lu.L[1]**	268	23 000	<0.01
**Rho-Pip-PEG$_4$-TCO, 10**	567	115 000	17.15
**L[2]**	568	90 800	19.70
**Eu.L[2]**	568	64 800	19.31
**Gd.L[2]**	568	65 600	17.41
**Tb.L[2]**	568	48 200	20.49
**Dy.L[2]**	568	81 800	19.73
**Lu.L[2]**	568	44 500	19.20

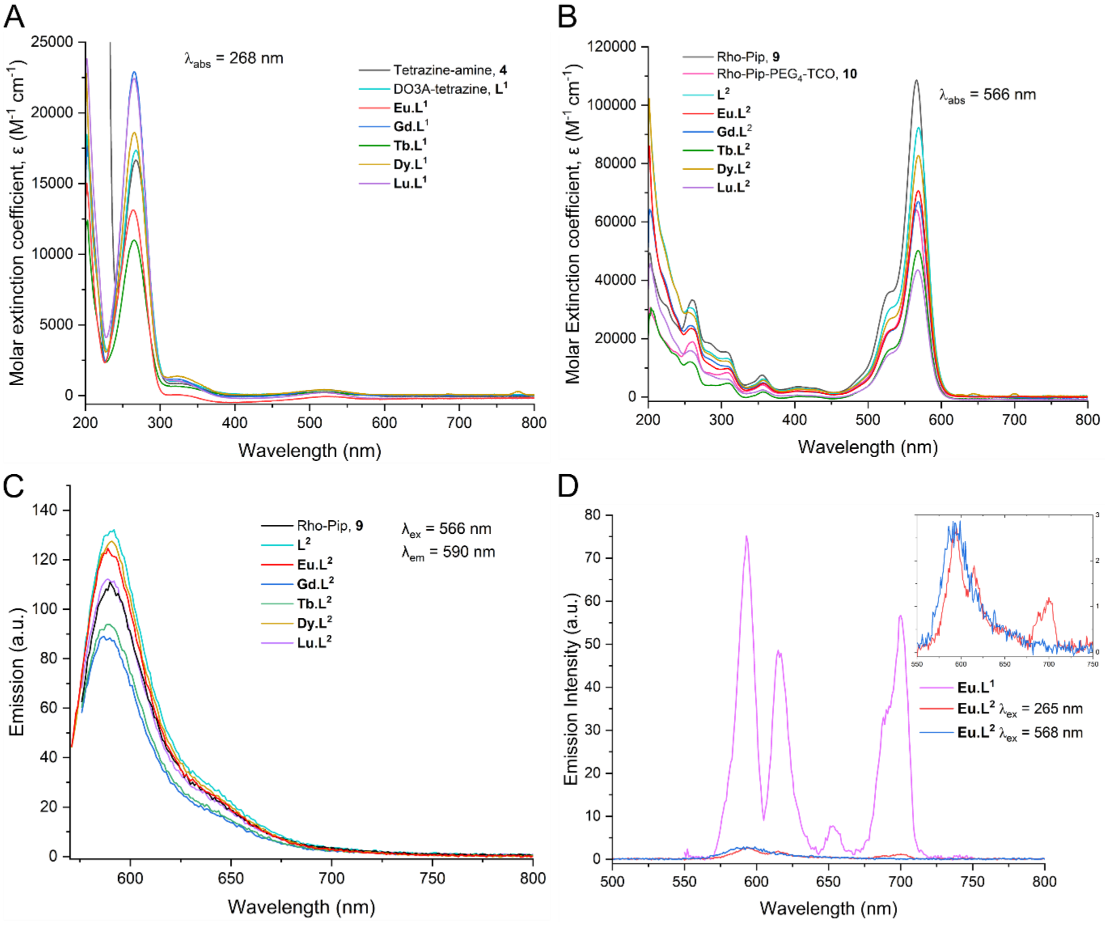

*Figure S2. Absorbance spectra of the tetrazine precursors, **4, L[1]** and **Ln.L[1]**, at 20 µM, λ_{abs} = 265 nm (A) and the rhodamine precursors, **9** and **10**, and click products, **L[2]** and **Ln.L[2]**, at 5 µM, λ_{abs} = 566 nm (B) in PBS buffer (pH = 7.4). C) Fluorescence spectrum of the rhodamine precursor and click products, **9**, **L[2]** and **Tb.L[2]**, when λ_{ex} = 566 nm at 5 µM in PBS buffer (pH = 7.4). D) Phosphorescence spectrum of the Eu complexes prior- (**Eu.L[1]**, λ_{ex} = 265 nm) and post-IEDDA cycloaddition (**Eu.L[2]**) excited at 265 and 568 nm at 20 µM in PBS (pH = 7.4).*

4. NMR Characterisation

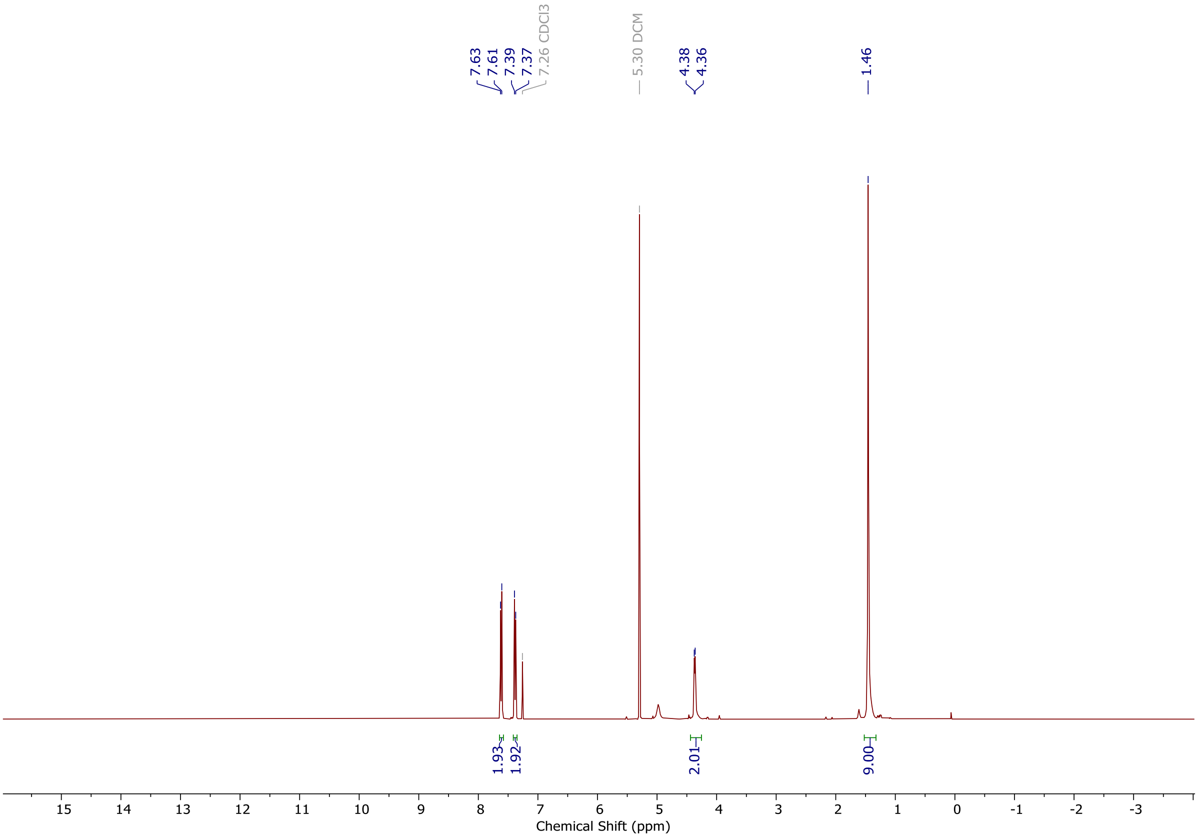

*Figure S3. ^1H NMR (400 MHz, $CDCl_3$) spectrum of tert-butyl N-(4-cyanobenzyl)carbamate, **1**.*

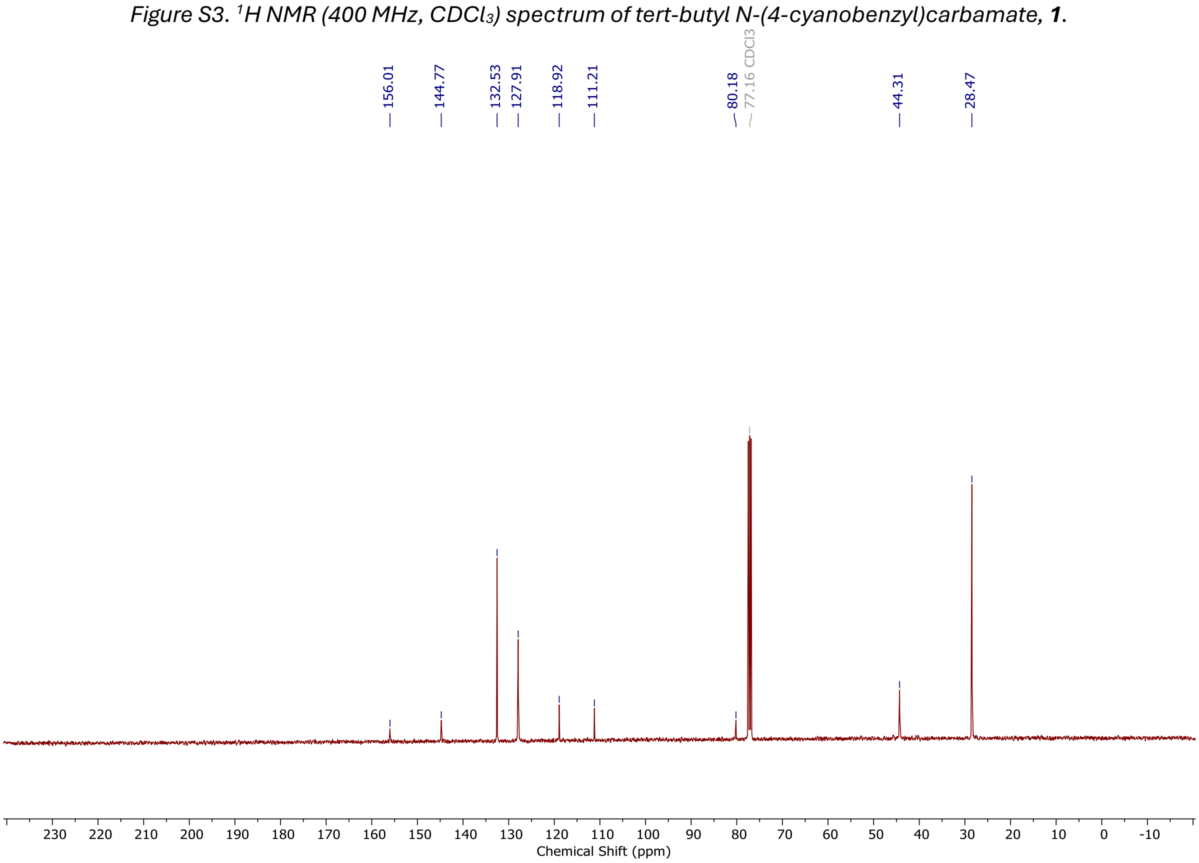

*Figure S4. ^{13}C NMR (101 MHz, $CDCl_3$) spectrum of tert-butyl N-(4-cyanobenzyl)carbamate, **1**.*

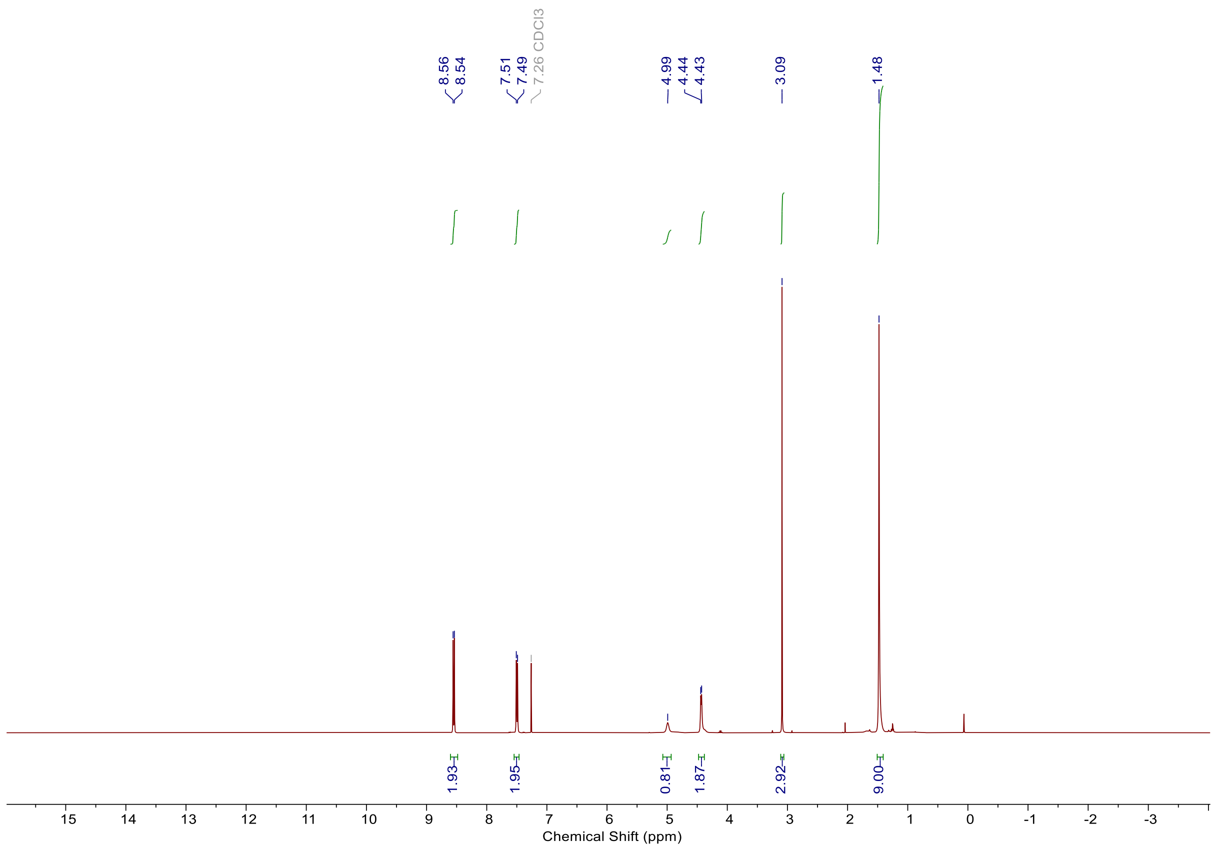

Figure S5. ^{1}H NMR (400 MHz, $CDCl_3$) spectrum of tert-butyl (4-(6-methyl-1,2,4,5-tetrazin-3-yl)benzyl)carbamate, ***2****.*

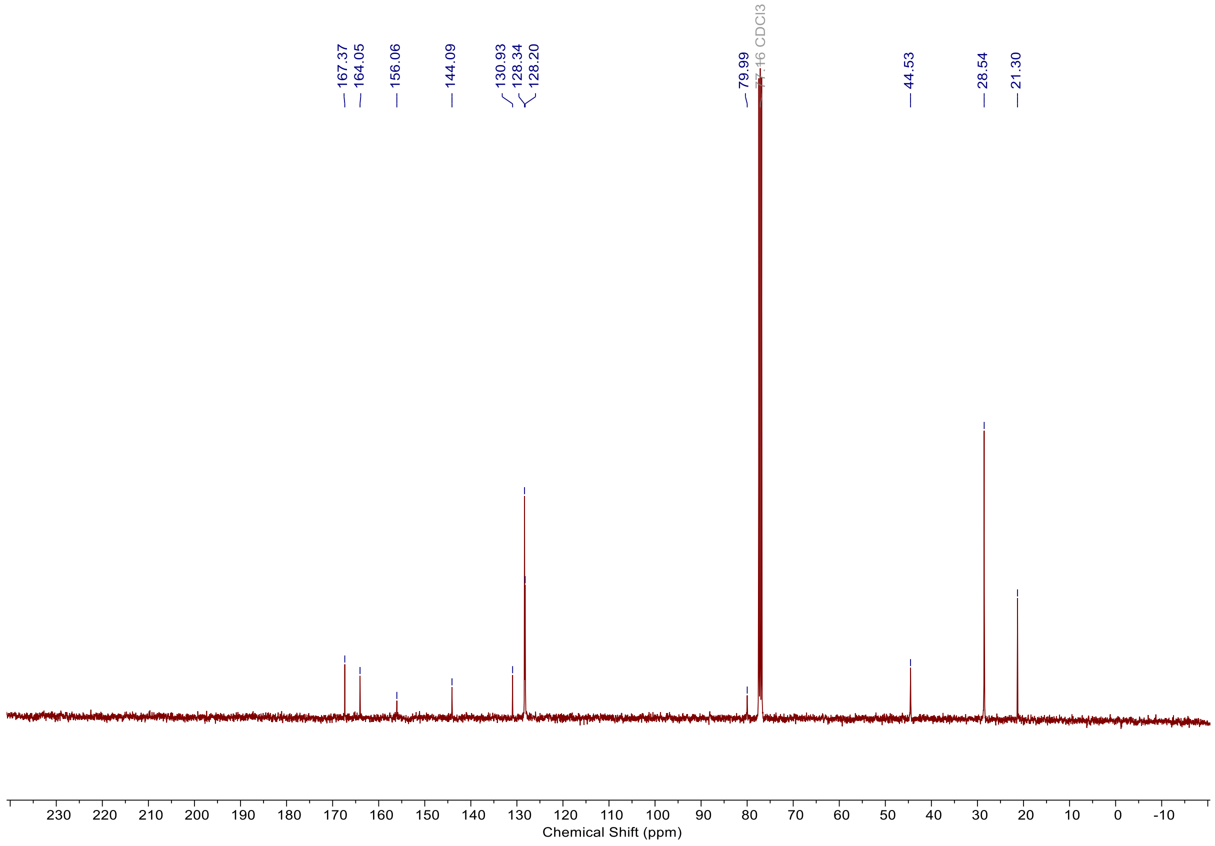

Figure S6. ^{13}C NMR (101 MHz, $CDCl_3$) spectrum of tert-butyl (4-(6-methyl-1,2,4,5-tetrazin-3-yl)benzyl)carbamate, ***2****.*

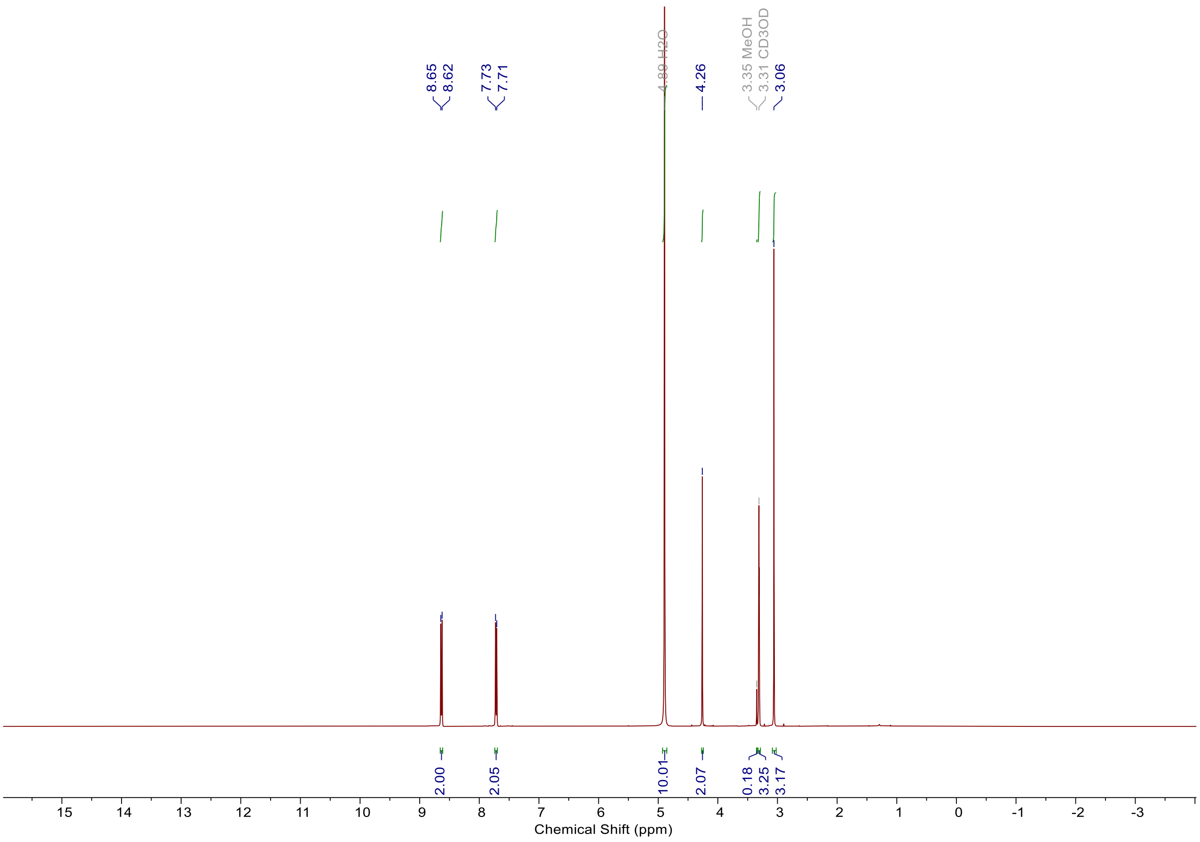

*Figure S7. ^{1}H NMR (400 MHz, CD_3OD) spectrum of (4-(6-methyl-1,2,4,5-tetrazin-3-yl)phenyl)methanaminium-2,2,2-trifluoroacetate, **3**.*

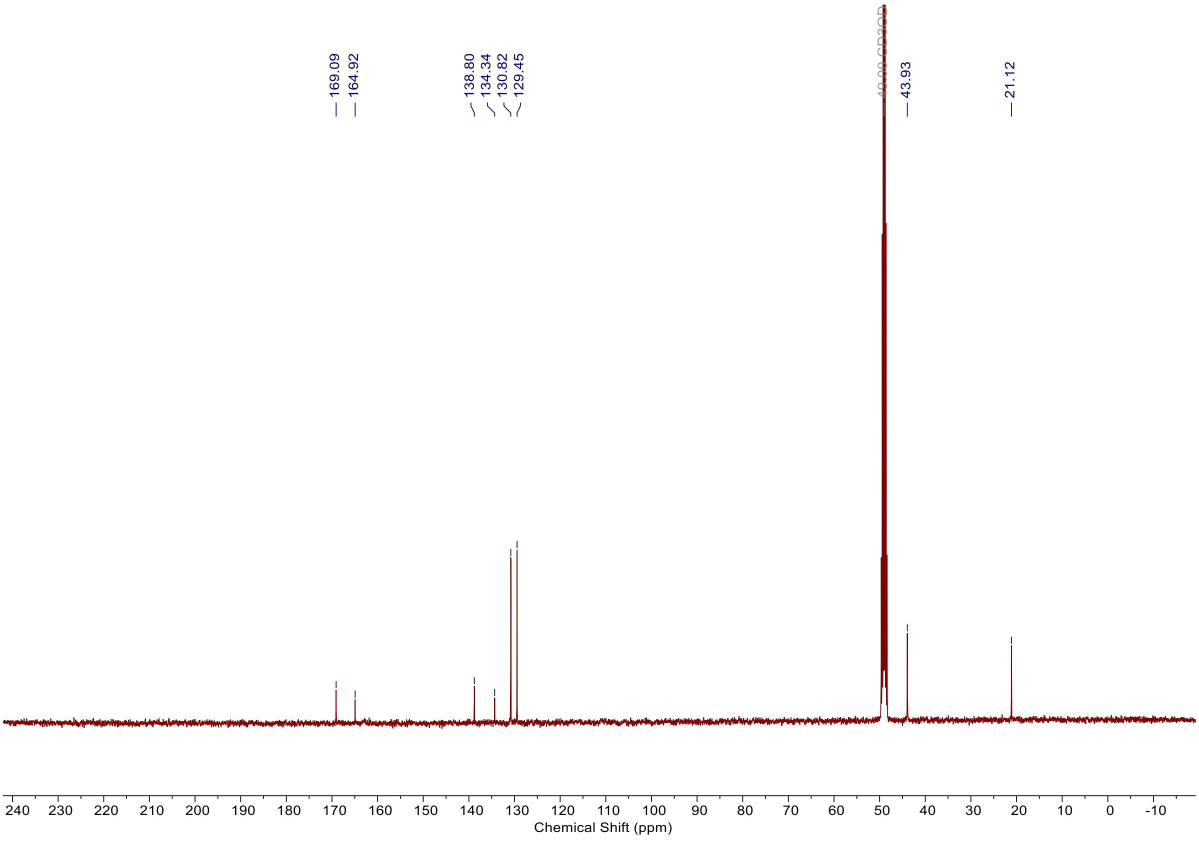

*Figure S8. ^{13}C NMR (101 MHz, CD_3OD) spectrum of (4-(6-methyl-1,2,4,5-tetrazin-3-yl)phenyl)methanaminium-2,2,2-trifluoroacetate, **3**.*

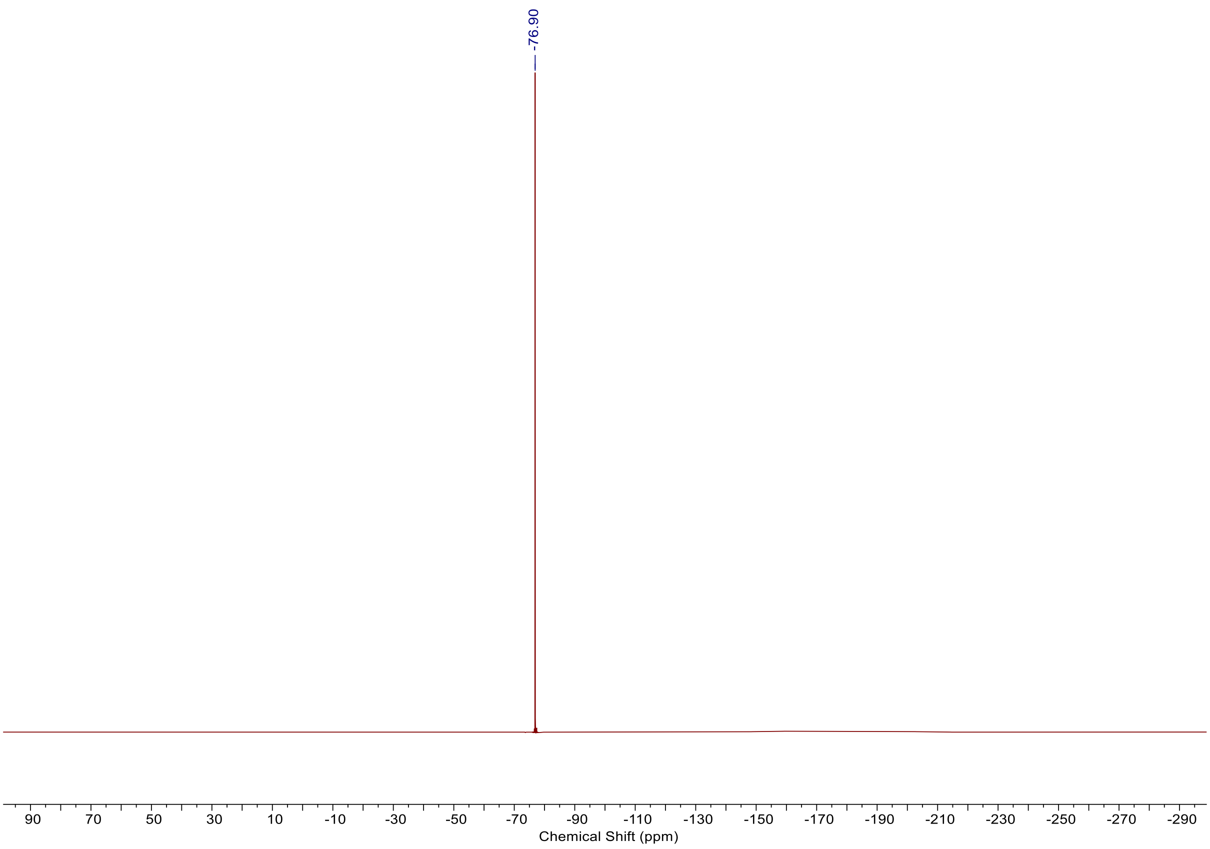

*Figure S9. ^{19}F NMR (377 MHz, CD_3OD) spectrum of (4-(6-methyl-1,2,4,5-tetrazin-3-yl)phenyl)methanaminium-2,2,2-trifluoroacetate, **3**.*

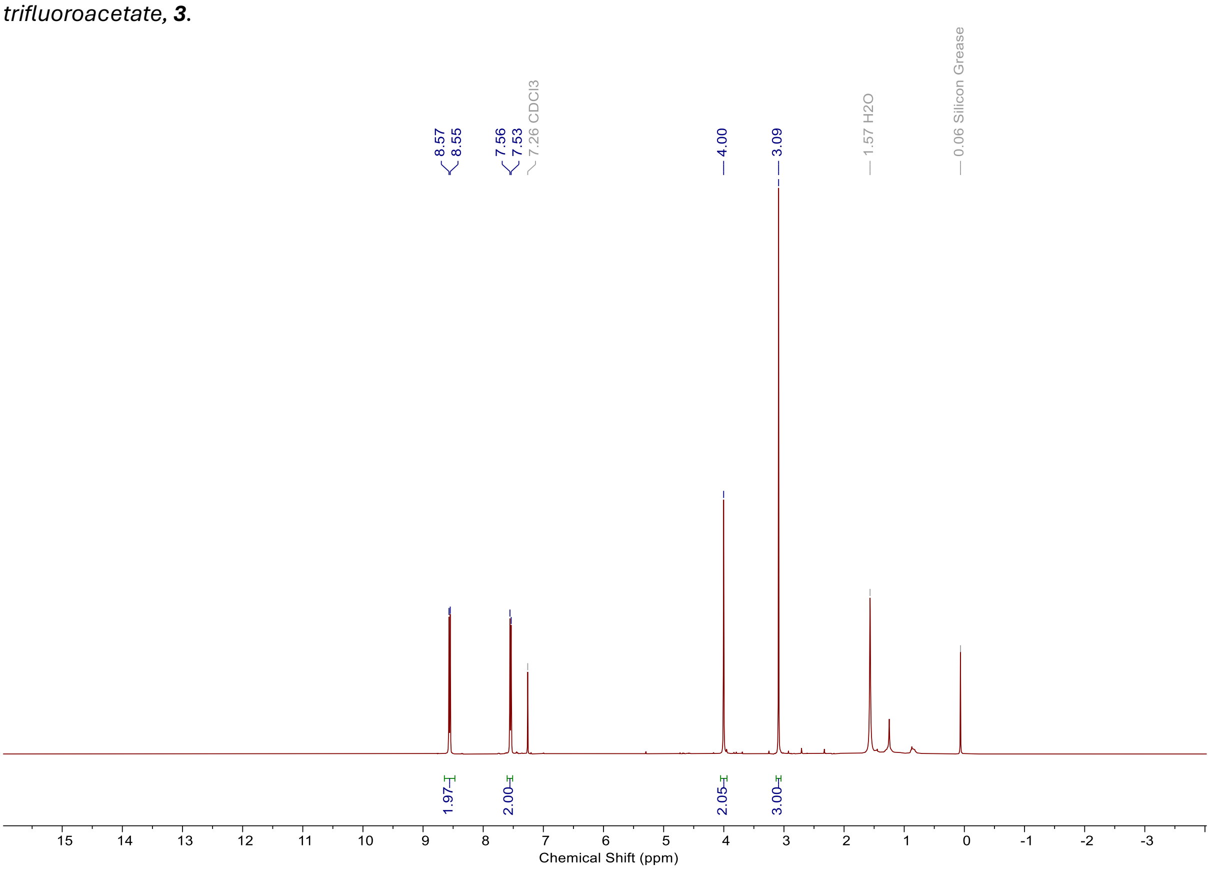

*Figure S10. ^{1}H NMR (400 MHz, $CDCl_3$) spectrum of (4-(6-methyl-1,2,4,5-tetrazine-3-yl)phenyl) methanamine, **4**.*

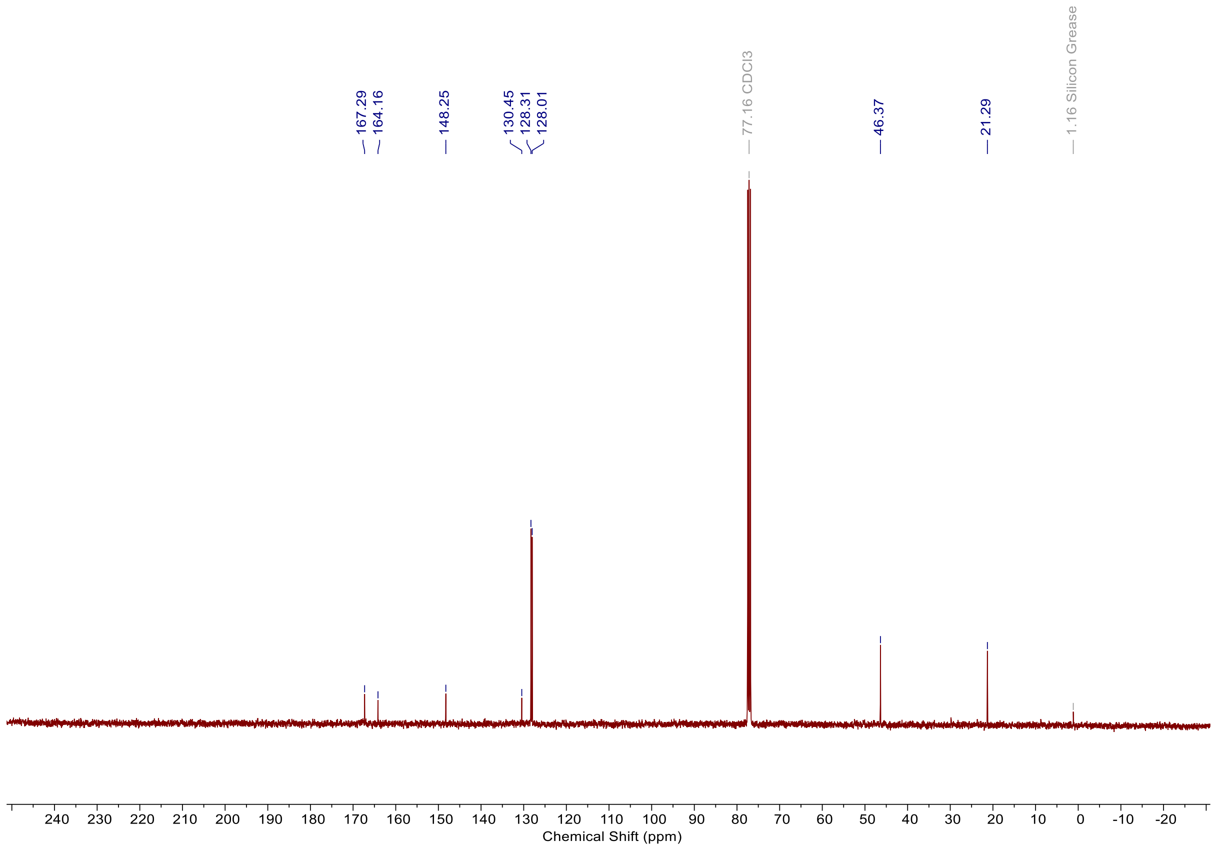

*Figure S11. ^{13}C NMR (101 MHz, $CDCl_3$) spectrum of (4-(6-methyl-1,2,4,5-tetrazine-3-yl)phenyl) methanamine, **4**.*

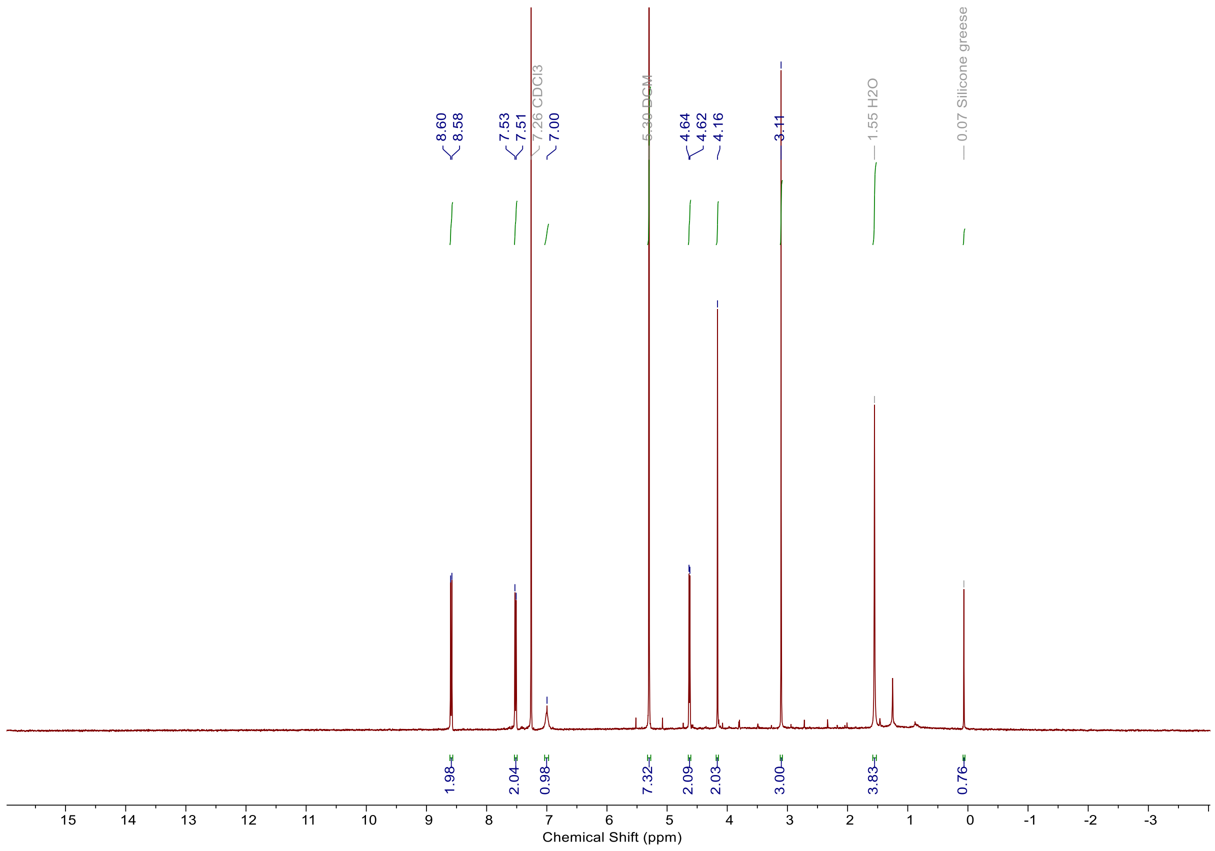

*Figure S12. ^{1}H NMR (400 MHz, $CDCl_3$) spectrum of 2-chloro-N-(4-(6-methyl-1,2,4,5-tetrazin-3-yl)benzyl)acetamide, **5**.*

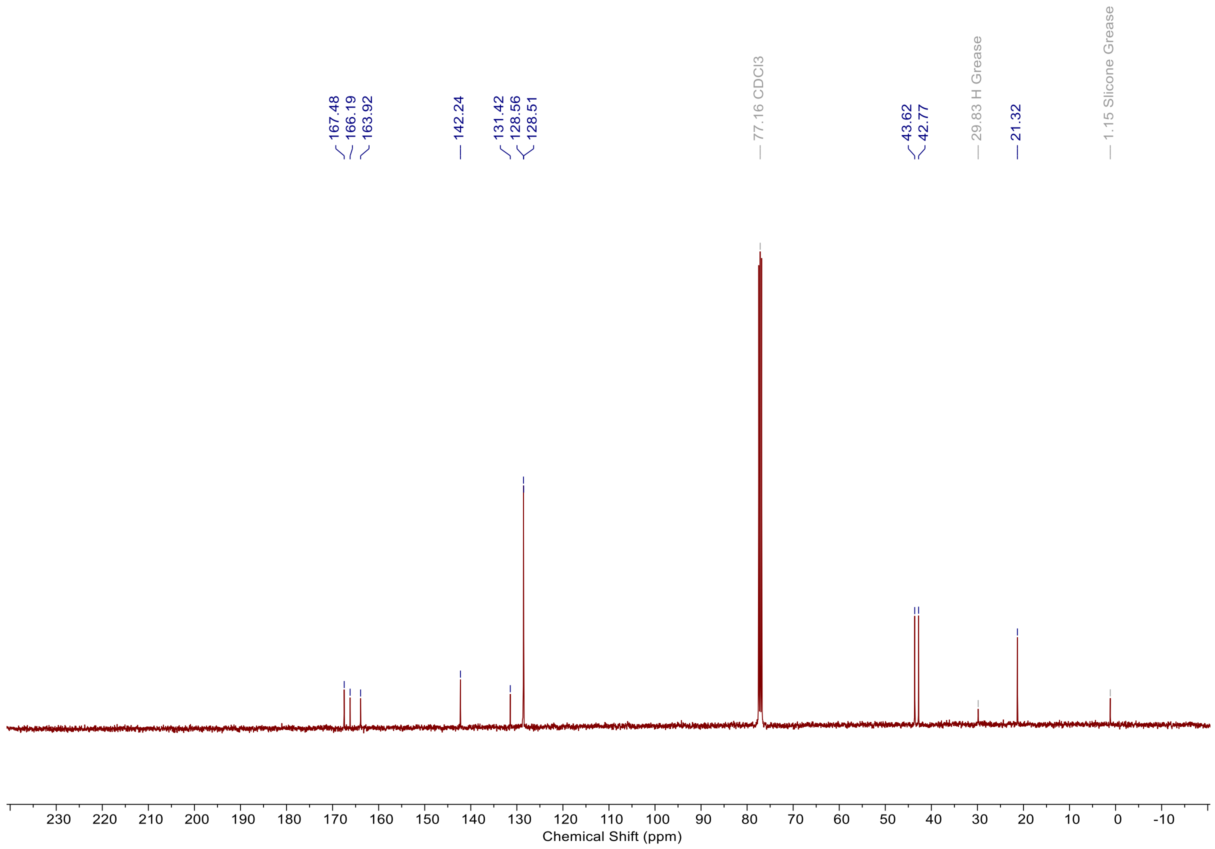

Figure S13. ^{13}C NMR (101 MHz, $CDCl_3$) spectrum of 2-chloro-*N*-(4-(6-methyl-1,2,4,5-tetrazin-3-yl)benzyl)acetamide, **5**.

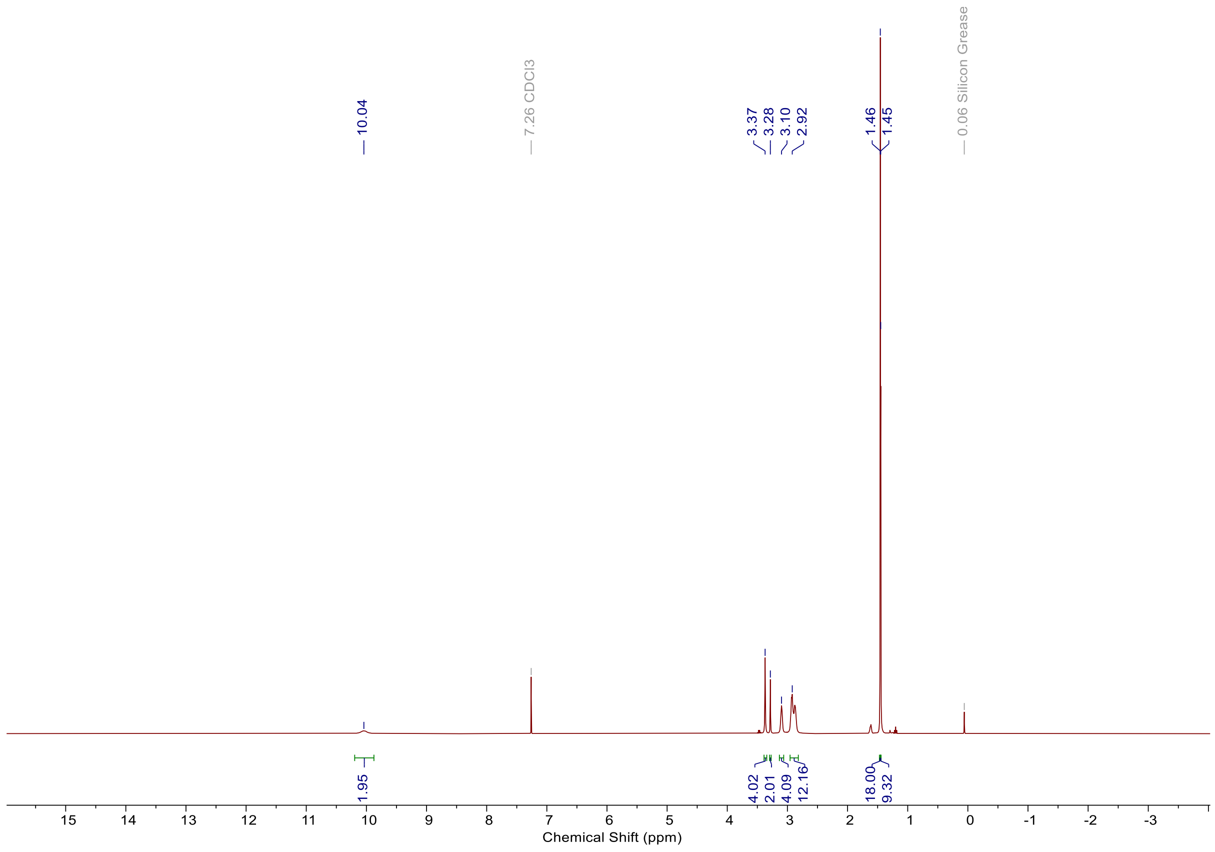

Figure S14. ^{1}H NMR (400 MHz, $CDCl_3$) spectrum of tert-butyl-DO3A.HBr, ***6****.*

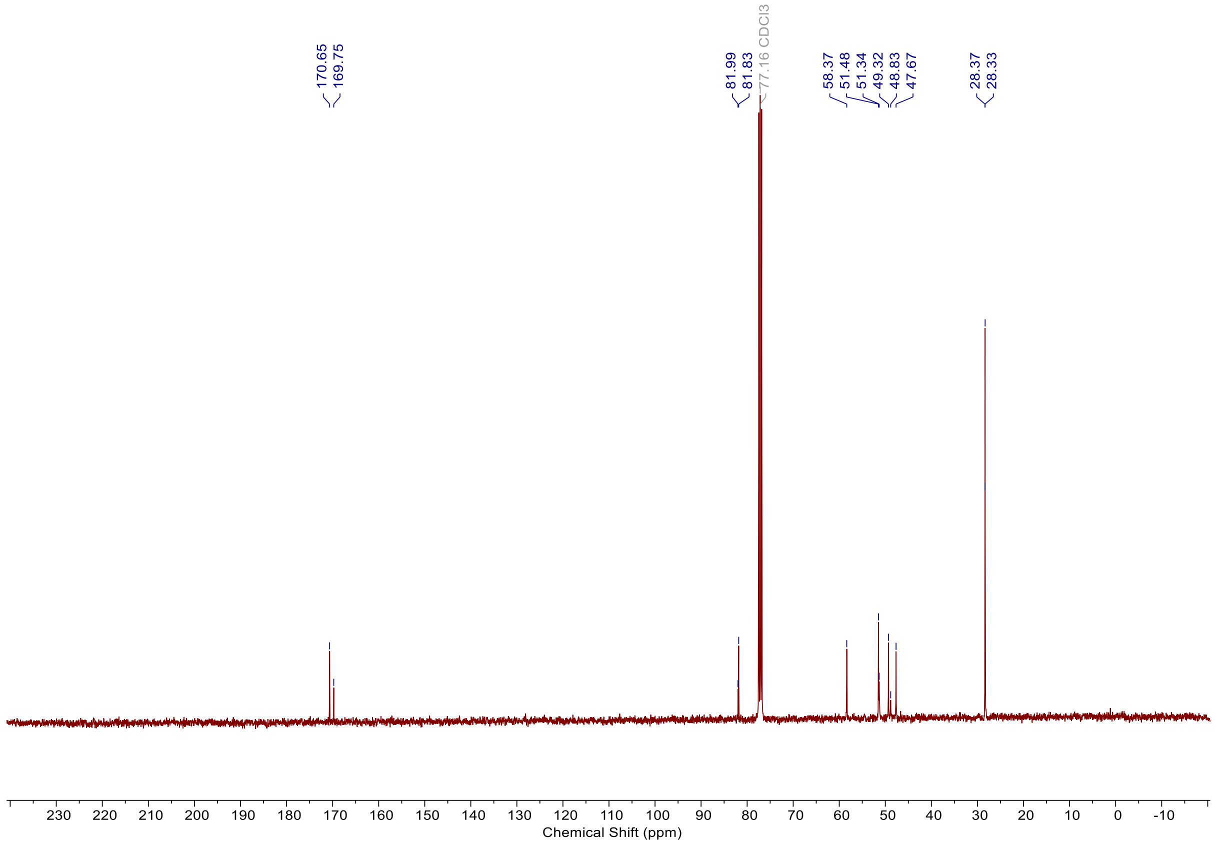

*Figure S15. ^{13}C NMR (101 MHz, $CDCl_3$) spectrum of tert-butyl-DO3A.HBr, **6**.*

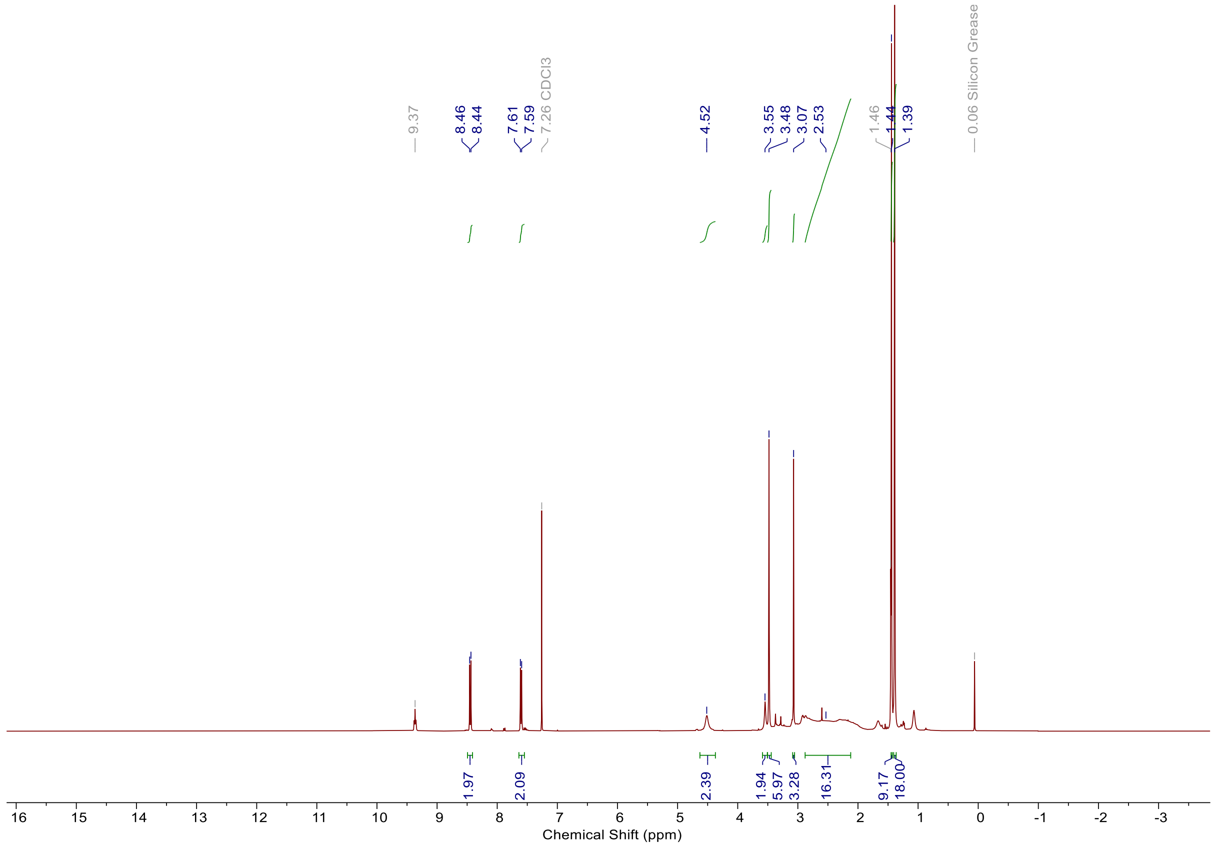

*Figure S16. ^{1}H NMR (400 MHz, $CDCl_3$) spectrum of tert-butyl-DO3A-tetrazine, **7**.*

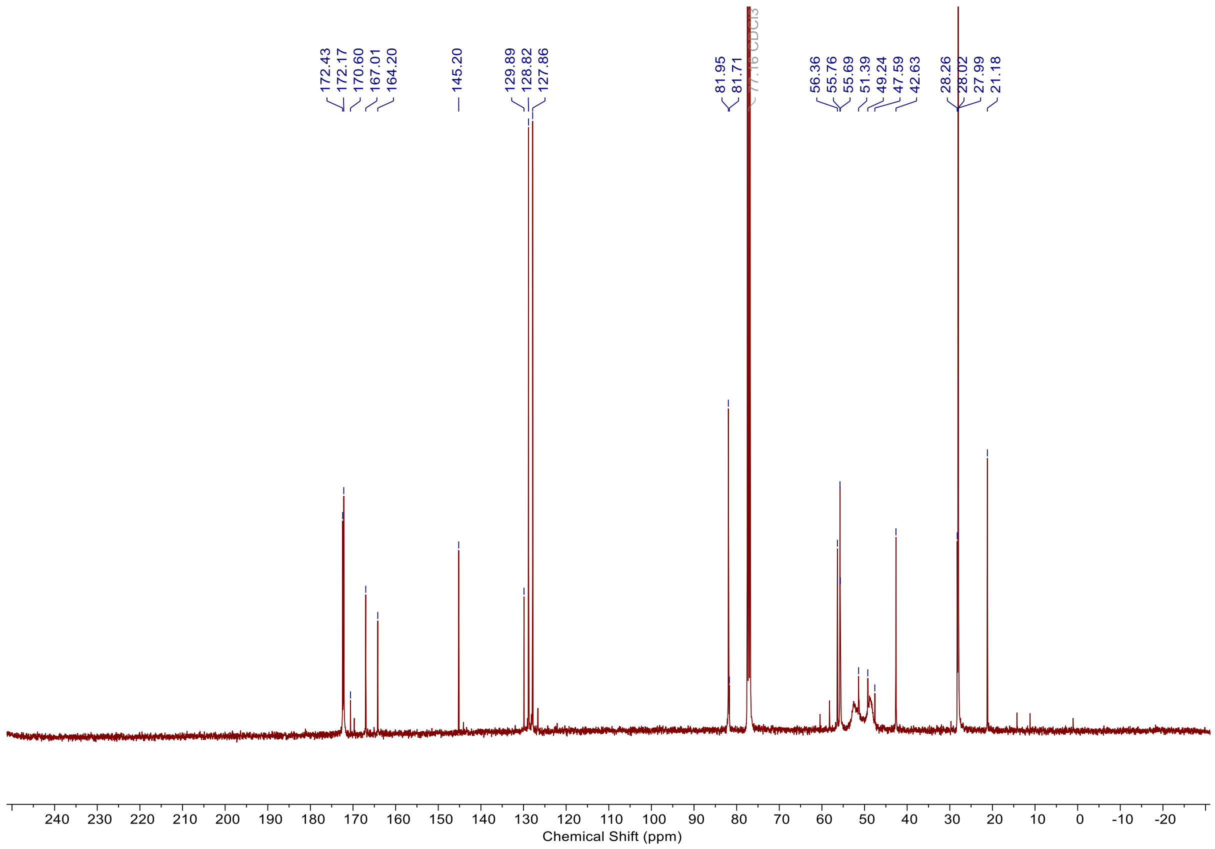

*Figure S17. ^{13}C NMR (101 MHz, $CDCl_3$) spectrum of tert-butyl-DO3A-tetrazine, **7**.*

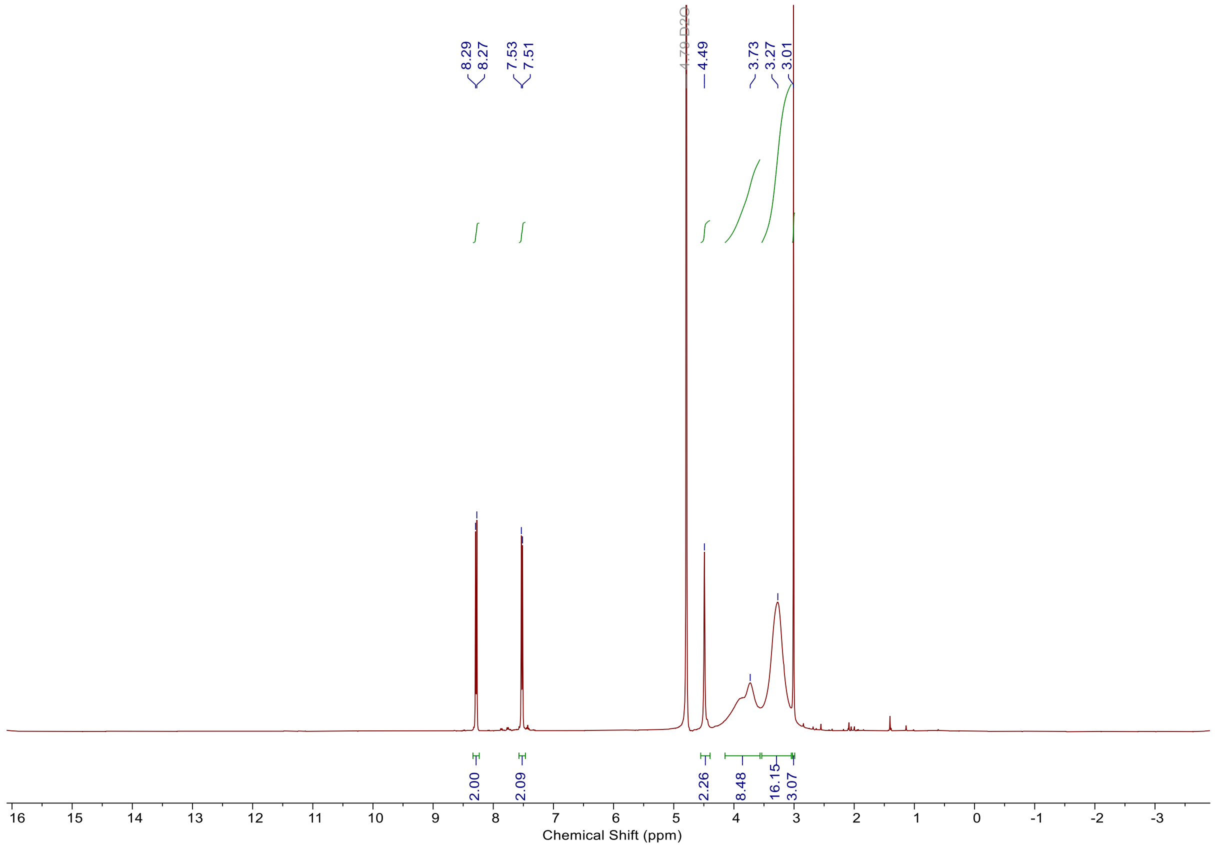

*Figure S18. ^{1}H NMR (400 MHz, D_2O) spectrum of DO3A-tetrazine, **L^1**.*

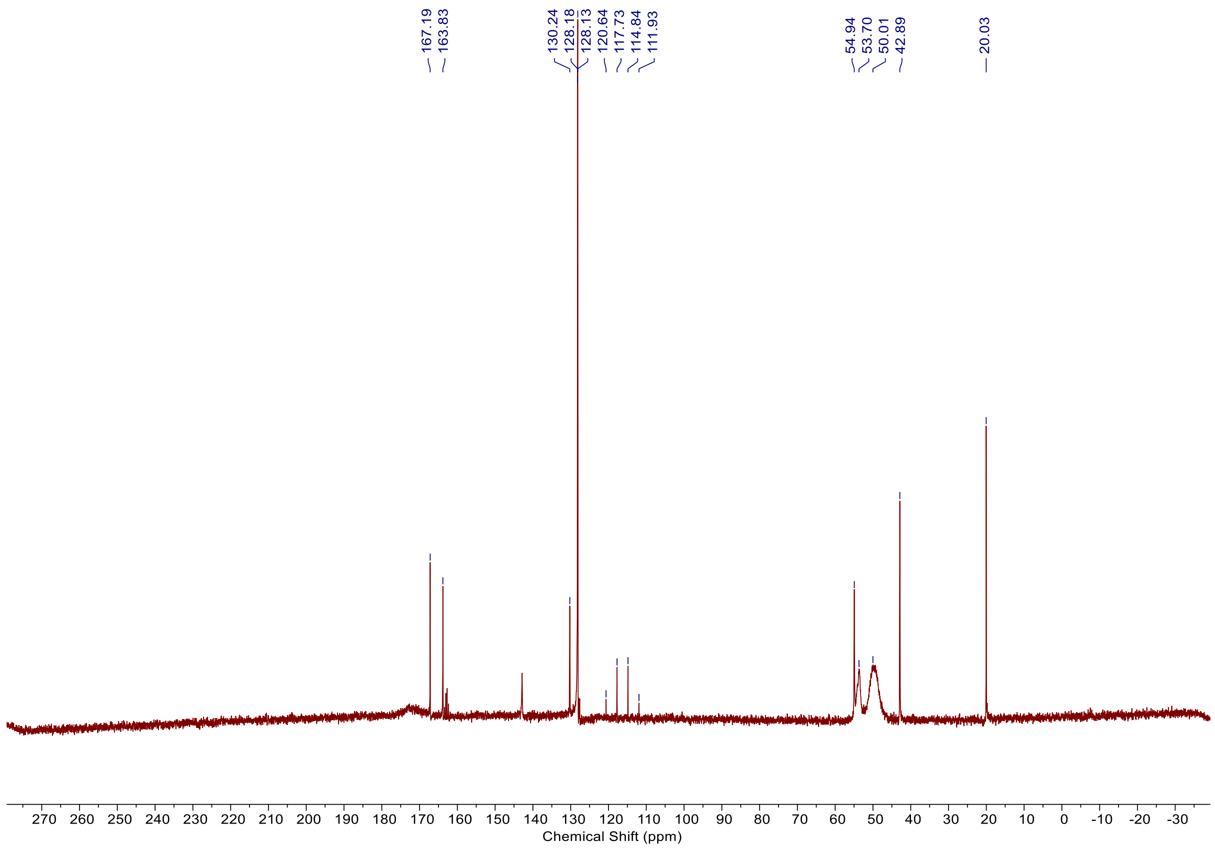

*Figure S19. ^{13}C NMR (101 MHz, D_2O) spectrum of DO3A-tetrazine, **L[1]**.*

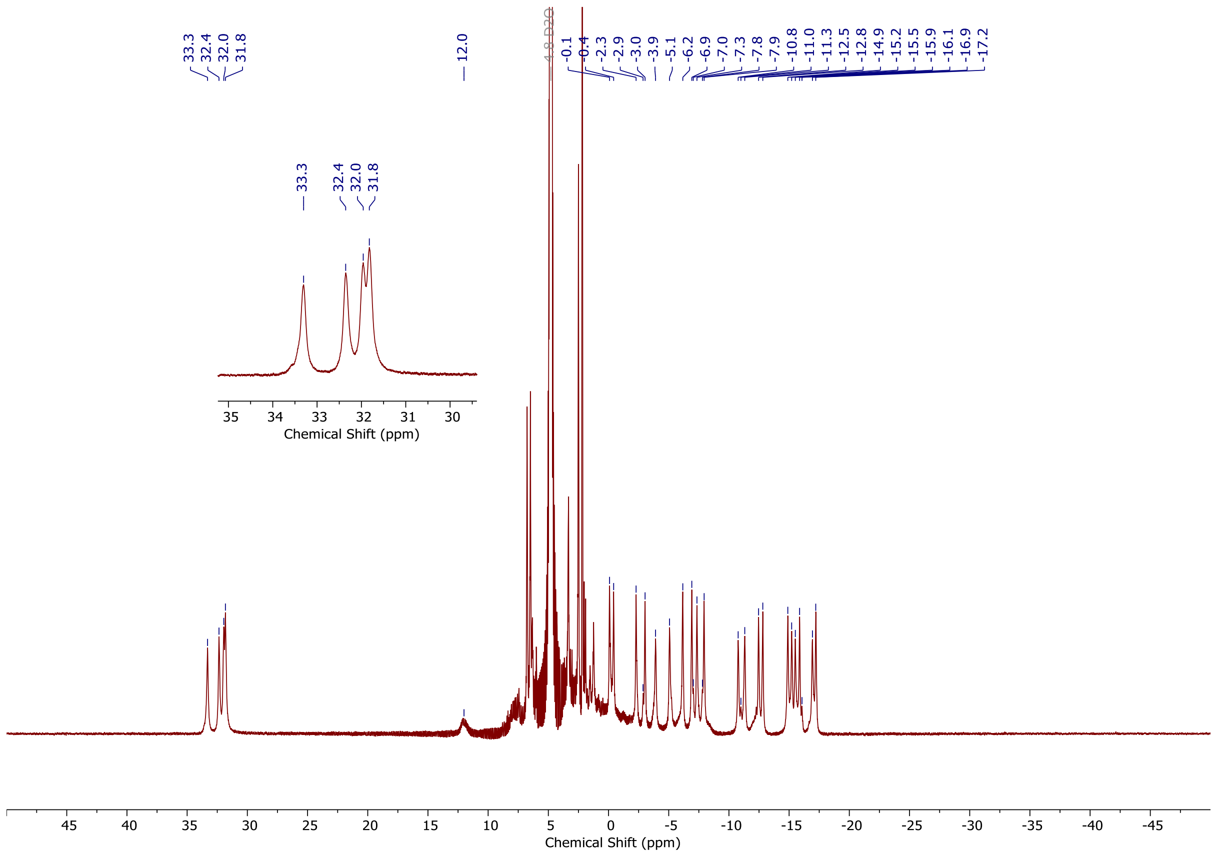

*Figure S20. ^{1}H-NMR (400 MHz, D_2O) spectrum of Eu(DO3A-tetrazine), **Eu.L[1]**.*

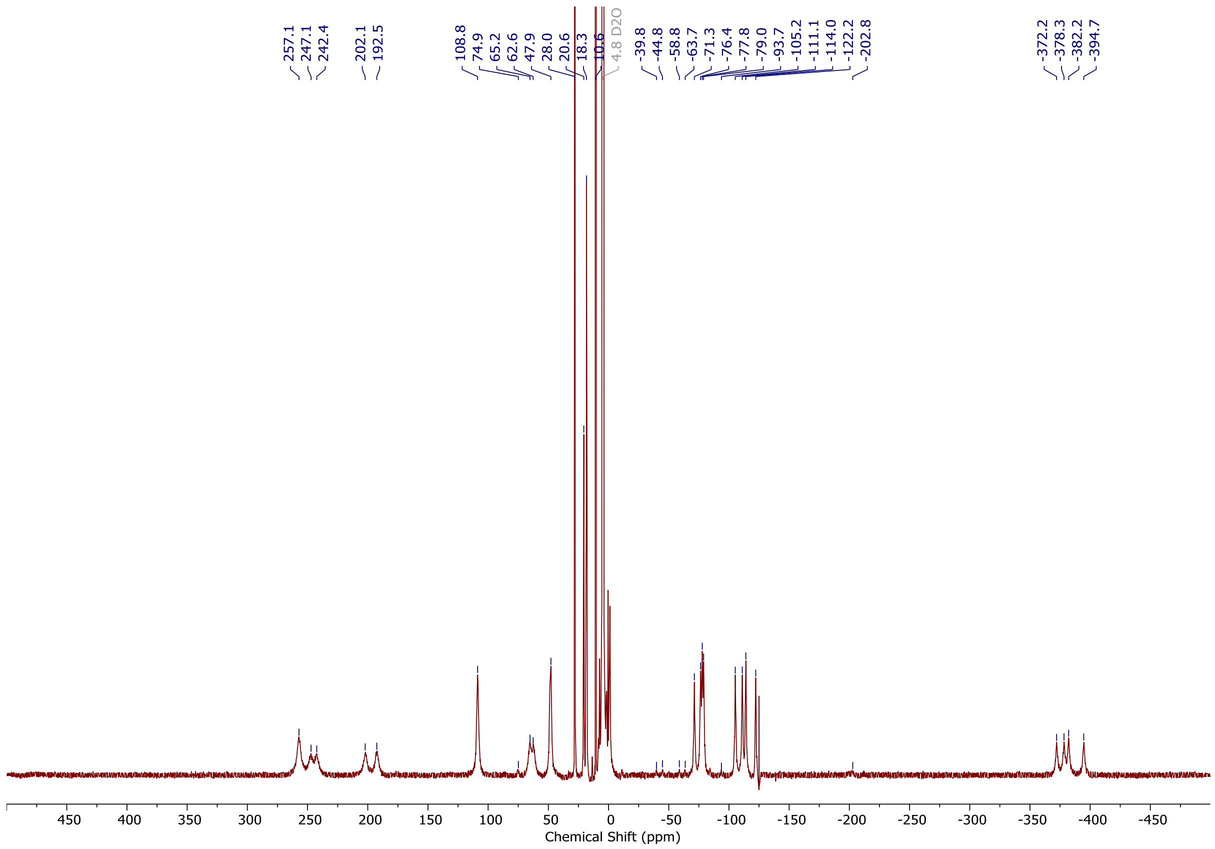

Figure S21. ¹H NMR (400 MHz, D_2O) spectrum of Tb(DO3A-tetrazine), ***Tb.L*****¹**.

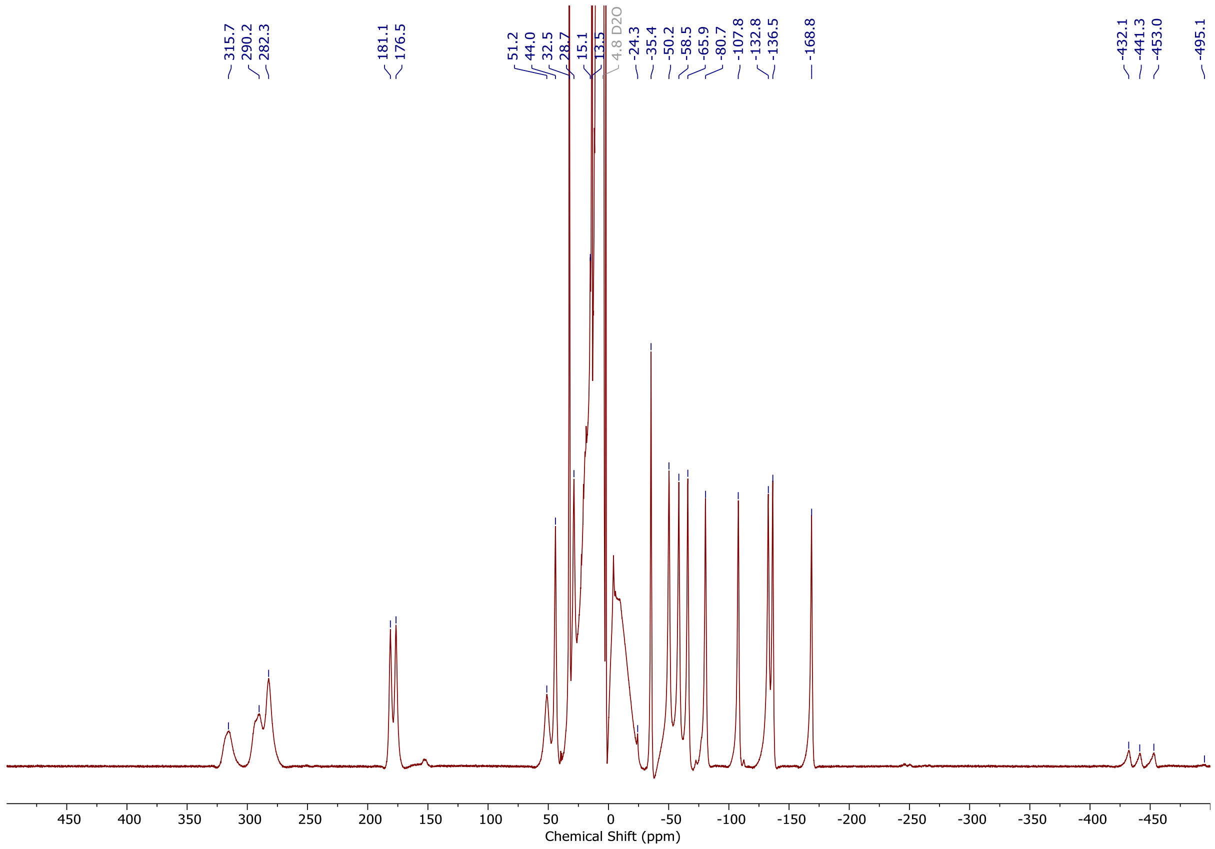

Figure S22. ¹H NMR (500 MHz, D_2O) spectrum of Dy(DO3A-tetrazine), ***Dy.L*****¹**.

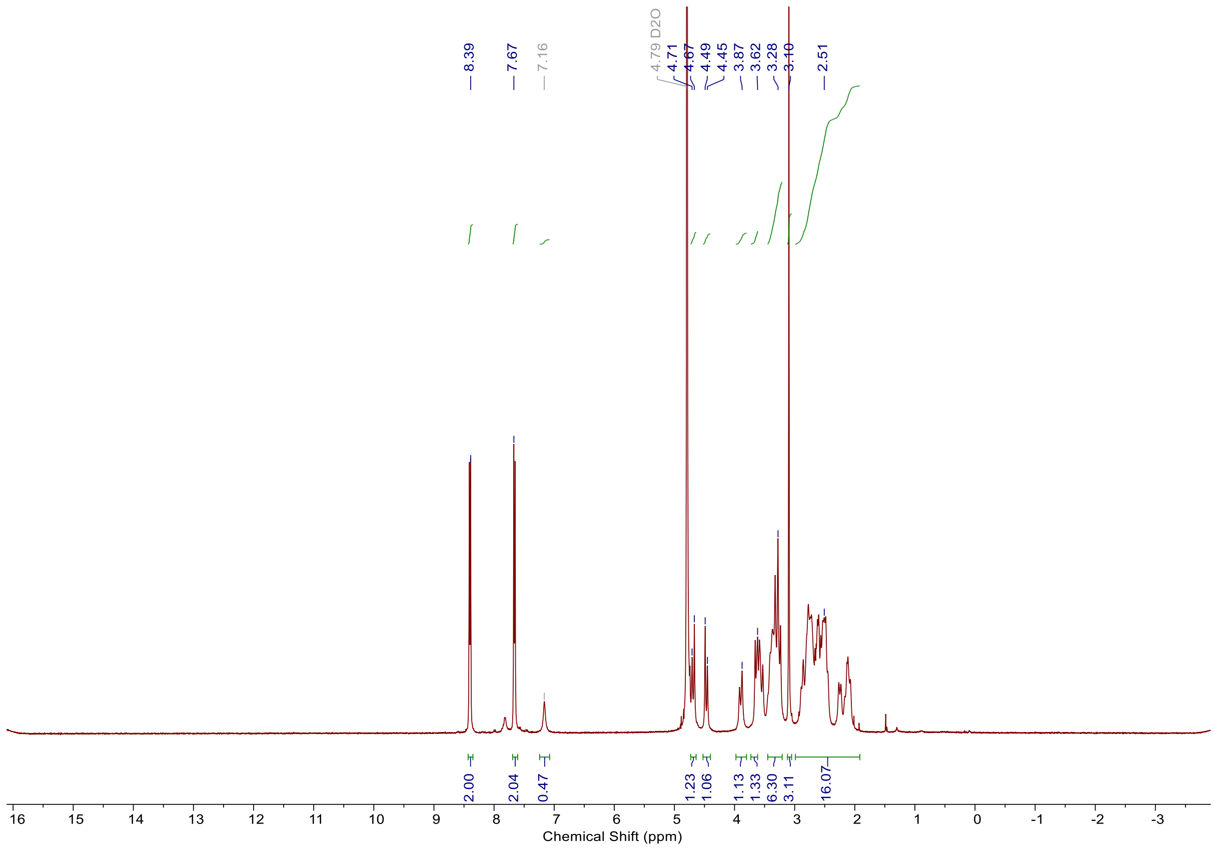

*Figure S23. ^{1}H NMR (400 MHz, D_2O) spectrum of Lu(DO3A-tetrazine), **Lu.L[1]**.*

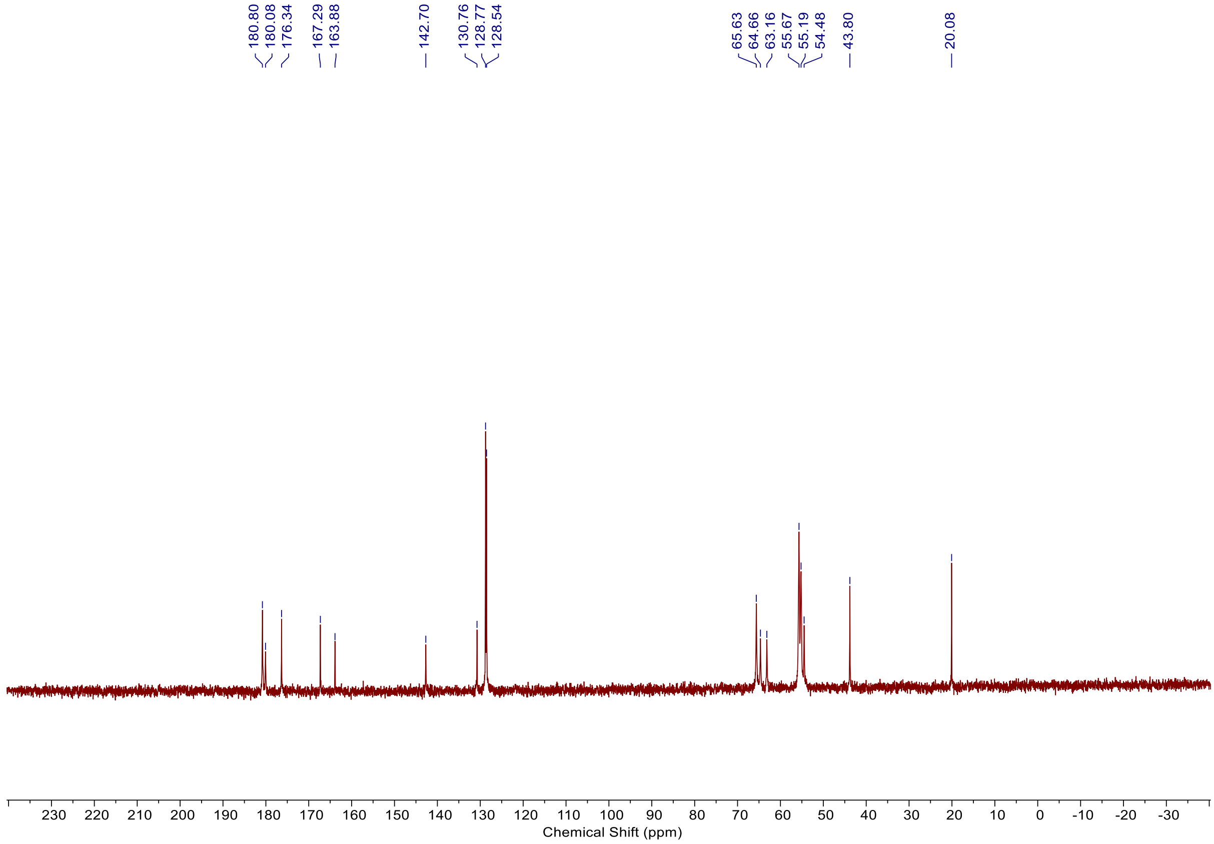

*Figure S24. ^{13}C NMR (101 MHz, D_2O) spectrum of Lu(DO3A-tetrazine), **Lu.L[1]**.*

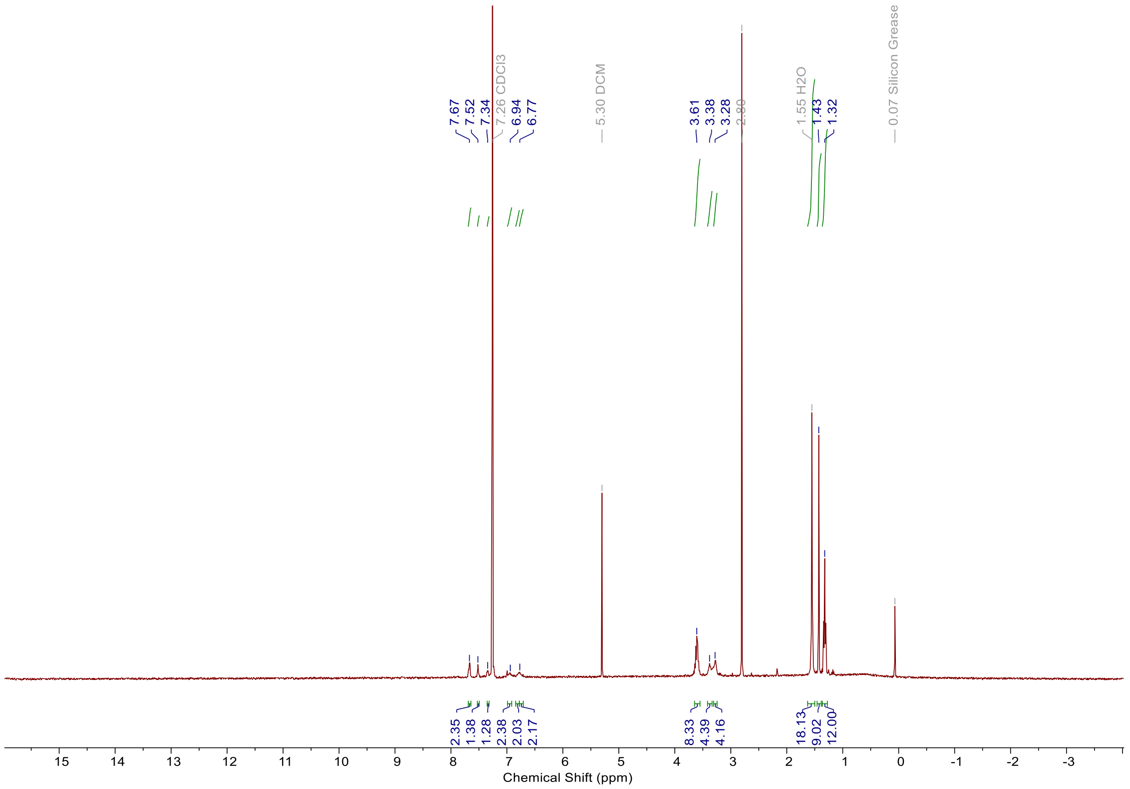

*Figure S25. ¹H NMR (400 MHz, CDCl₃) spectrum of boc-rhodamine-piperazine, **8**.*

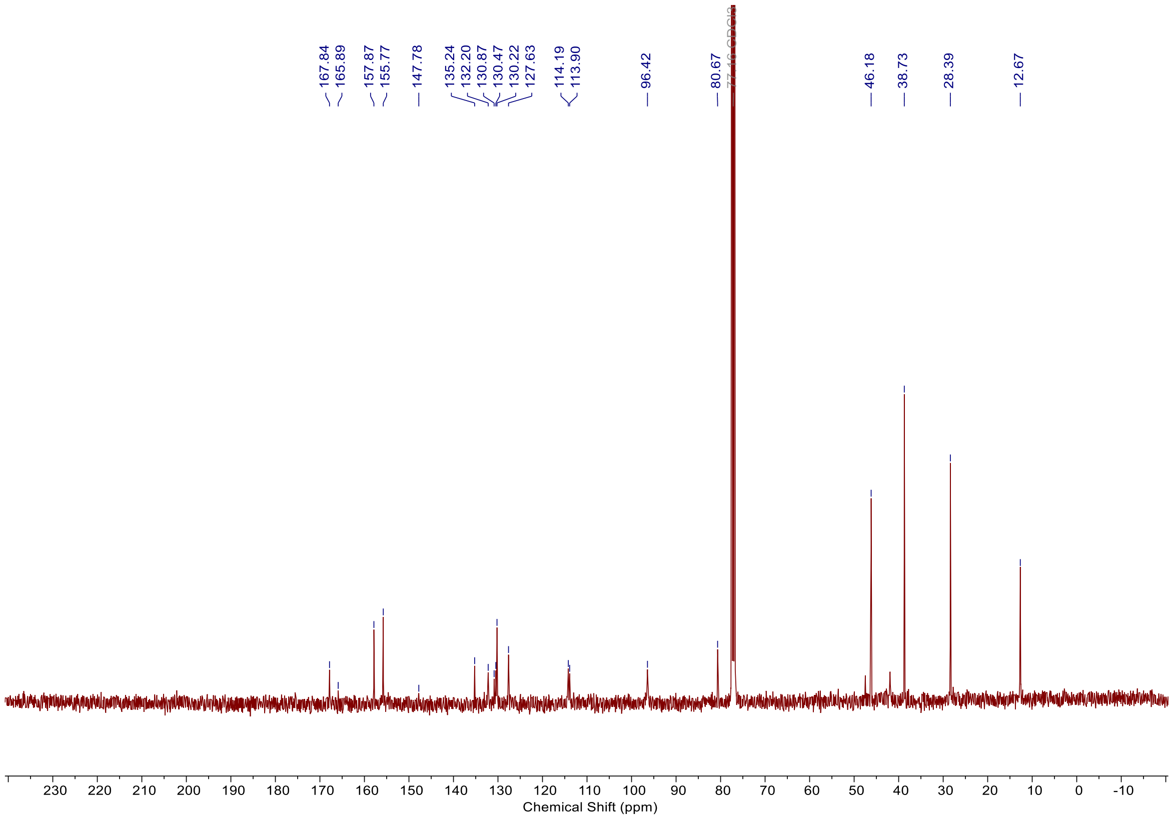

*Figure S26. ¹³C NMR (101 MHz, CDCl₃) spectrum of boc-rhodamine-piperazine, **8**.*

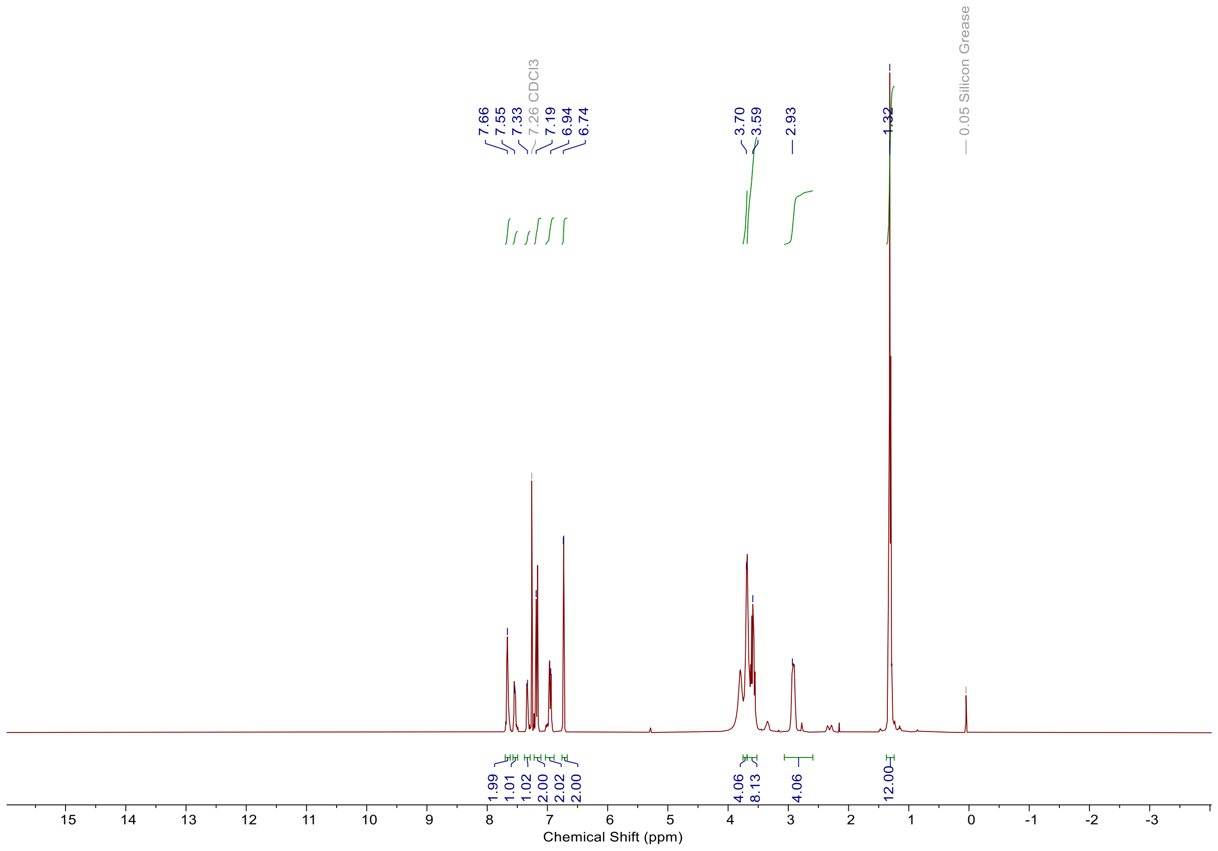

Figure S27. ¹H NMR (400 MHz, $CDCl_3$) spectrum of rhodamine-piperazine, ***9***.

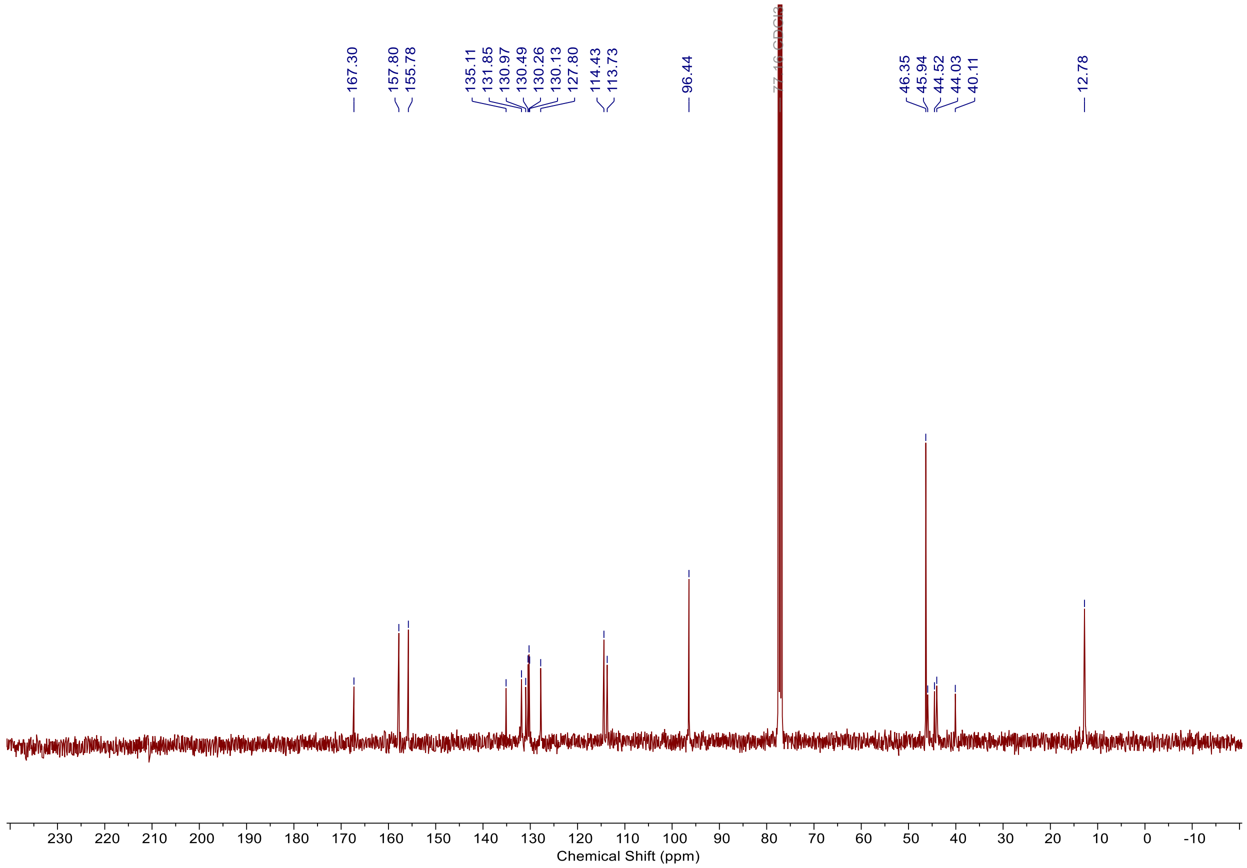

Figure S28. ¹³C NMR (101 MHz, $CDCl_3$) spectrum of rhodamine-piperazine, ***9***.

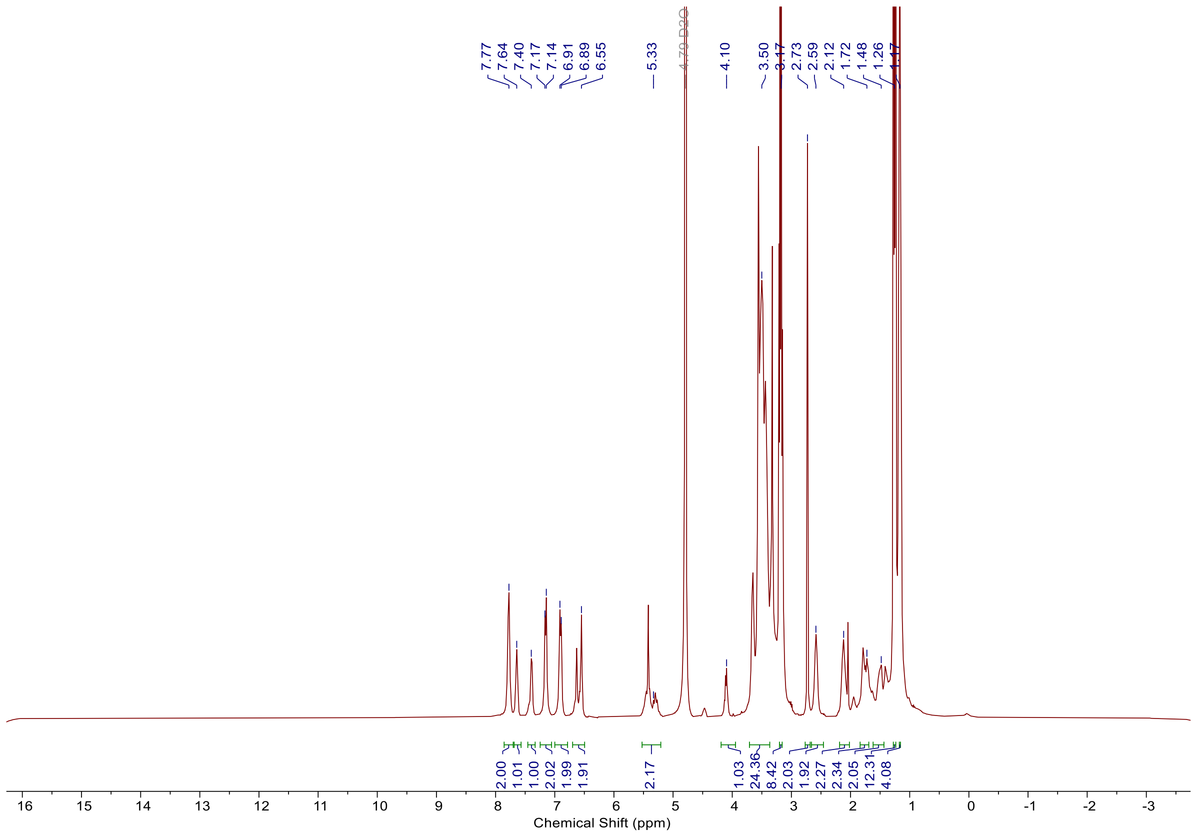

Figure S29. ¹H NMR (400 MHz, D_2O) spectrum of rhodamine-piperazine-PEG_4-TCO, ***10****.*

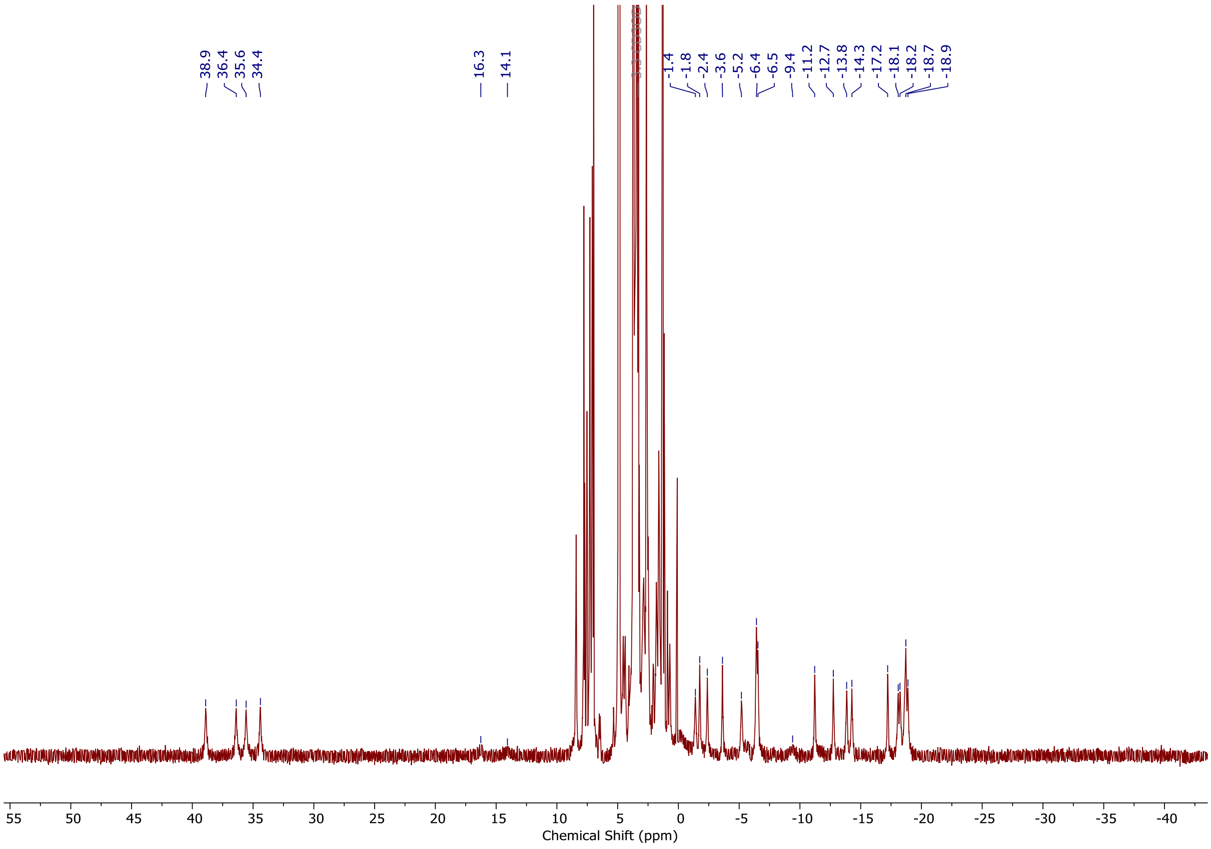

Figure S30. ¹H NMR (400 MHz, CD_3OD) spectrum of Eu(DO3A-tetrazine)-PEG_4-piperazine-rhodamine, ***Eu.L²****.*

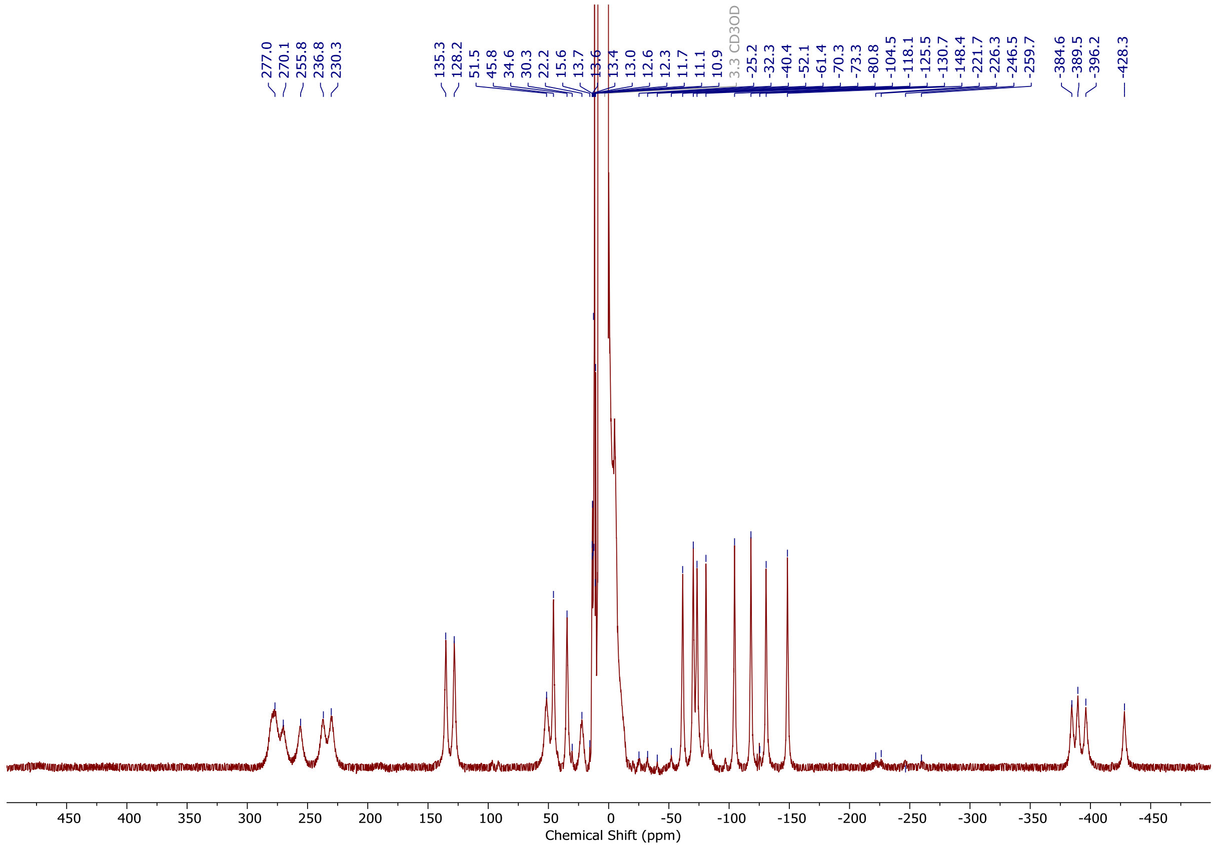

Figure S31. [1]*H NMR (400 MHz, CD_3OD) spectrum of Tb(DO3A-tetrazine)-PEG$_4$-piperazine-rhodamine,* ***Tb.L^2***.

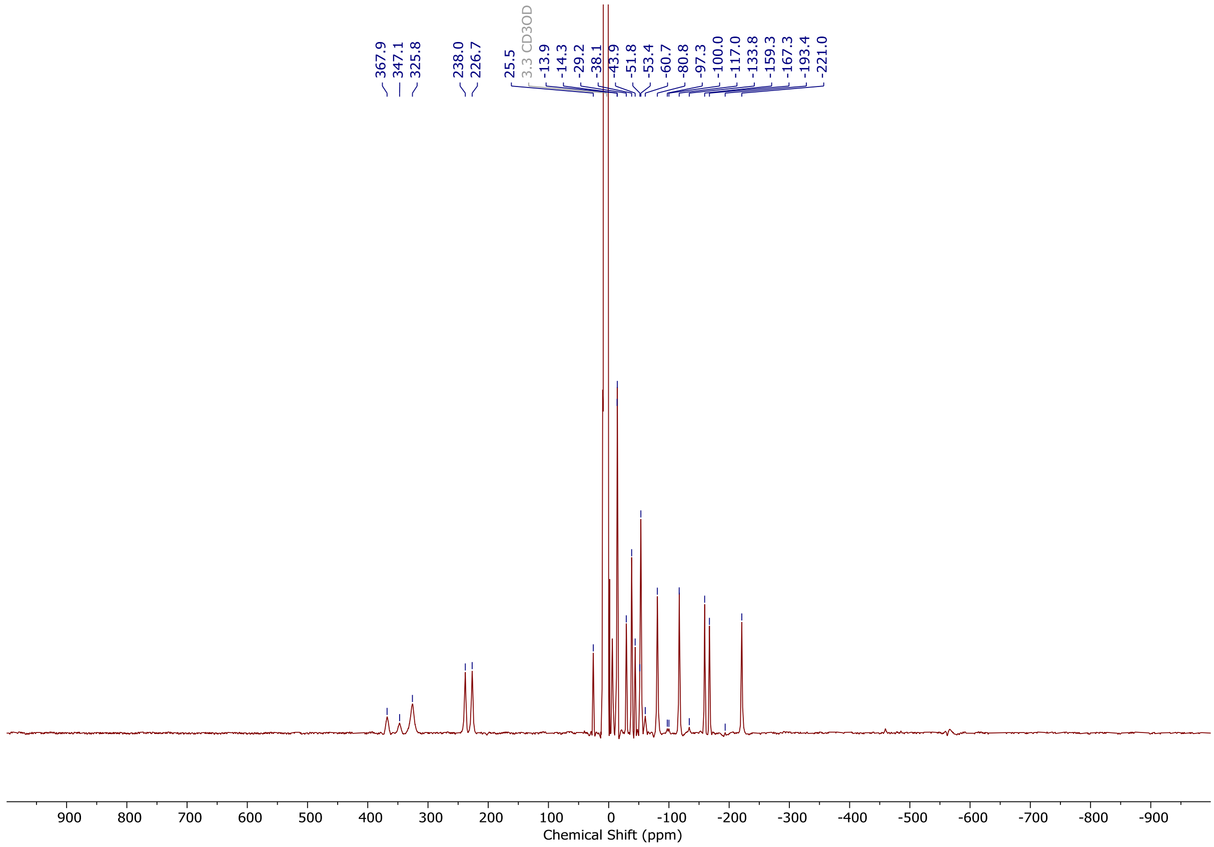

Figure S32. [1]*H NMR (500 MHz, D_2O) spectrum of Dy(DO3A-tetrazine)-PEG$_4$-piperazine-rhodamine,* ***Dy.L^2***.

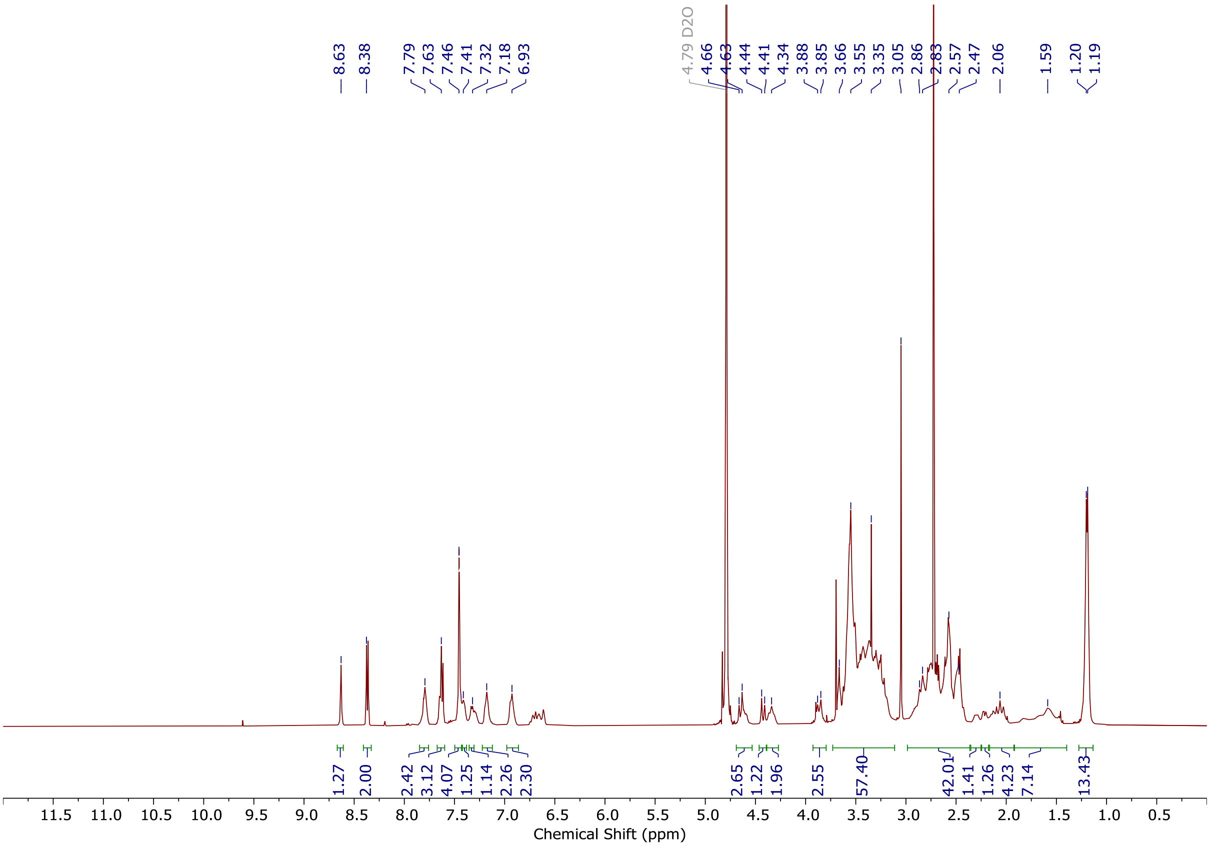

Figure S33. ^{1}H NMR (500 MHz, D_2O) spectrum of Lu(DO3A-tetrazine)-PEG_4-piperazine-rhodamine, ***Lu.L²***.

5. Ultrasound Set-up

The focused ultrasound-assisted deliveries of **Tb.L²** and **Lu.L²** were carried out using a single-element spherical-segment focused ultrasound transducer (center frequency: 1 MHz; active diameter: 90 mm; focal depth: 60.5 mm; part number: H-198; Sonic Concepts, Bothell, WA, USA). The emitted ultrasound beam was defined by the full width at half maximum (FWHM) of the beam and measured 1.40 mm in the elevational dimension, 1.75 mm in the lateral dimension and 10.7 mm in length. A spherically focused passive cavitation detector (center frequency: 7.5 MHz; diameter: 12.7 mm; focal length: 76.2 mm; part number: U8423539; Olympus Industrial, Essex, UK) was fitted into the central cavity of the emitting transducer for the real-time monitoring of microbubble cavitation activity in the cerebral vasculature during the sonication procedure. Received emission signals were filtered by a band-pass filter (3-30 MHz; part number: ZABP-16 + ; Mini-Circuits, Brooklyn, NY, USA), pre-amplifier (28 dB, Stanford Research Systems, Sunnyvale, CA, USA) and recorded by a 14-bit oscilloscope (GaGe model Octave Express CompuScope; sampling rate: 100 MS/s; DynamicSignals, Lockport, IL, USA).

The deliveries of **Gd.L²** were carried out using a single-element spherical-segment focused ultrasound transducer (center frequency: 1.05 MHz; active diameter: 64 mm; focal depth: 60.8 mm; part number: H-117; Sonic Concepts, Bothell, WA, USA). Defined by the FWHM, the emitted ultrasound beam measured 1.89 mm in the elevational dimension, 1.90 mm in the lateral dimension and 13.0 mm in length.

A function generator (33500B Series; Agilent Technologies, Santa Clara, CA, USA) generated the ultrasound sequence (**Tb.L²** and **Lu.L²**: center frequency: 1 MHz; peak-negative pressure: 0.80 MPa *in situ*; pulse length: 10 ms; burst repetition frequency: 0.5 MHz; cycles: 126. **Gd.L²**: center frequency: 1.05 MHz; peak-negative pressure: 0.53 MPa *in situ*; pulse length: 10 ms; burst repetition frequency: 0.5 MHz; cycles: 126). The signal was amplified by a 50-dB power amplifier and passed through an electrical impedance matching network constructed in-house.

6. *In Vivo* Sonication Procedures

All procedures were approved by the UK Home Office and Imperial College London's Animal Welfare and Ethical Review Body and performed in compliance with the UK Animals (Scientific Procedures) Act 1986 and ARRIVE guidelines.[8]

Animals were held in a room at a controlled temperature (20-24 °C) and humidity (45-65%), and a 12:12 light cycle, including a dawn (30 min) and dusk (30 min) period. Food (RM1 expanded pellets) and water were provided ad libitum.

Five mice (C57BL/6J, wild-type, 12-16 weeks, female, 20.9 ± 1.6 g, Charles River, Cambridge, UK) were administered with **Tb.L²** (n=1), **Lu.L²** (n=1) or **Gd.L²** (n=3) and microbubbles, and their brains exposed to focused ultrasound.

Mice were anaesthetised with a mixture of 1.5-2.0% vaporized isoflurane (Zoetis UK, Leatherhead, UK) and oxygen (1 L/min), dispensed by an anaesthetic vaporizer (MSS International, Keighley, UK). Fur was removed from the mouse's head and the mouse was held in position by a stereotaxic frame (45° ear bars; World Precision Instruments, Hitchin, UK).

6.1. Procedures with Tb.L² and Lu.L²

Ultrasound coupling gel was applied to the shaved mouse head and a water bath fitted with an acoustically transparent parafilm bottom was placed above the gel. A metal cross was positioned in the filled water bath such that its intersection aligned with the lambdoidal and sagittal sutures (lambda). A 10 mm x 10 mm raster scan was performed to determine the position of lambda and from this reference point, the beam was positioned over the left hippocampus, which was determined to be 3 mm lateral and 0.5 mm medial from lambda, and 3 mm inferior to the skull.

Mice were administered with **Tb.L²** (17.5 mg/kg in PBS, 100 µL) or **Lu.L²** (15.6 mg/kg in PBS, 100 µL) by tail-vein injection using a 30-gauge catheter over 60 seconds. The ultrasound sequence was then commenced and 14 seconds into the sequence, *SonoVue®* microbubbles (2 x 10^8 MBs/mL, 100 µL) (Bracco, Milan, Italy) were intravenously administered over 60 seconds. Upon completion of the sequence, mice were sacrificed immediately and transcardially perfused with PBS (20 mL) and 10% formalin (neutral buffered; 20 mL) (Sigma Aldrich, St. Louis, MO, USA) solutions.

The brain was extracted from the skull, further fixed in 10% formalin overnight, cryoprotected in 30% sucrose in PBS + 0.05% NaN_3 and sectioned into 30 µm-thick frozen horizontal sections.

6.2. Procedures with Gd.L²

The position of lambda was marked on the mouse's scalp and a camera fitted through the central cavity of the transducer was used to guide the transducer to this position above the skull. The beam was then positioned to be 3 mm lateral and 0.5 mm from lambda, and 3 mm inferior to the skull. Ultrasound coupling gel was applied to the mouse's head and a water bath fitted with an acoustically-transparent parafilm bottom was then positioned on top of the mouse's head such that the gel was compressed.

The ultrasound sequence was commenced and 14 seconds into the sequence, *SonoVue®* microbubbles (2 x 10^8 MBs/mL, 100 µL) (Bracco, Milan, Italy) were administered by tail-vein injection using a 30-gague catheter over 60 seconds. At 2 minutes after the start of the sequence, mice were administered with **Gd.L²** (31.1 mg/kg in PBS, 100 µL) by tail-vein injection over 60 seconds. Upon completion of the sequence, sacrificed immediately and transcardially perfused with PBS (20 mL) and 10% formalin (neutral buffered; 20 mL, Sigma Aldrich, St. Louis, MO, USA) solutions. The mouse head was excised and tissues removed from the outside of the skull. The mouse skull was stored in PBS + 0.05% NaN_3 prior to MR imaging. Following MR imaging, brains were excised from the skull, cryoprotected in 30% sucrose in PBS + 0.05% NaN_3 and sectioned into 30 µm-thick frozen horizontal sections. Kidneys and livers were excised, further fixed in 10% formalin overnight, cryoprotected in 30% sucrose + 0.05% NaN_3 and sectioned into 50 µm-thick frozen horizontal sections.

7. *Ex Vivo* MR Imaging

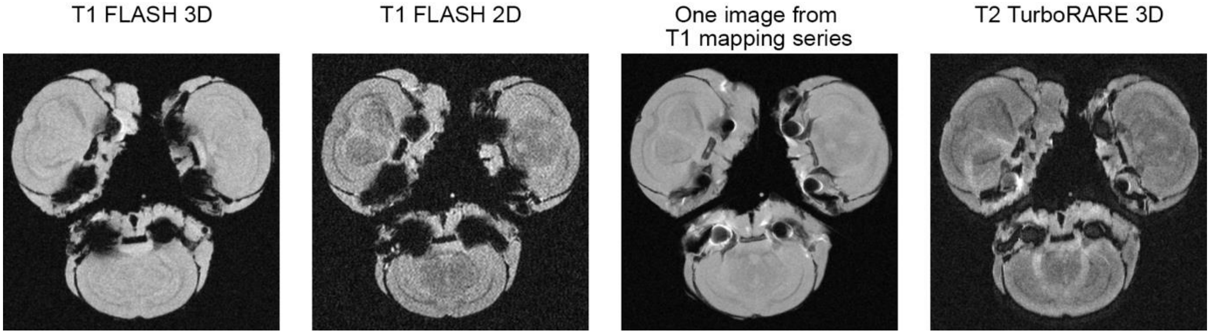

Figure S34. Ex vivo MR scans of mouse brains in the coronal plane using multiple sequences at 9.4 T.

Three in-skull mouse brain samples were placed in a custom-designed, 3D-printed plastic mouse brain holder inside a 50 mL syringe.[9] The syringe was filled with a proton-free liquid (Fluorinert™ Electronic Liquid FC-40, 3M, MN, USA) to prevent artefacts at tissue-air interface. Imaging was performed on a preclinical 9.4 T Biospec 94/20 MRI scanner (Bruker, Billerica, MA, USA) using multiple sequences.

- 3D FLASH: TR = 50 ms, TE = 7.2 ms, flip angle = 32, number of averages = 10, FOV = 25 mm × 25 mm × 32 mm, resolution = 0.1 mm × 0.1 mm × 0.1 mm.
- 2D FLASH: TR = 1800 ms, TE = 6.5 ms, flip angle = 56, number of averages = 10, FOV = 25 mm × 25 mm × 32 mm, resolution = 0.1 mm × 0.1 mm × 0.32 mm.
- 2D RAREVTR: TR = 5500, 3000, 1500, 1000, 800, 600, 400 ms, TE = 7.5 ms, RARE factor = 1, flip angle = 180, number of averages = 10, FOV = 25 mm × 25 mm × 32 mm, resolution = 0.1 mm × 0.1 mm × 1 mm.
- 3D TurboRARE: TR = 2500 ms, TE = 7.5 ms, RARE factor = 32, flip angle = 51.1, number of averages = 6, FOV = 25 mm × 25 mm × 32 mm, resolution = 0.1 mm × 0.1 mm × 0.1 mm.

8. Fluorescence Microscopy

Brain, liver and kidney sections were imaged by fluorescence widefield microscopy (objective: 10×/0.3 Ph1 EC Plan-Neofluar; working distance = 5.2 mm; Axio Observer, ZEISS, Oberkochen, Germany) with excitation at 550/25 nm (Spectra X Light Engine, Lumencor, Beaverton, OR, USA) and emissions detected at 605/70 nm.

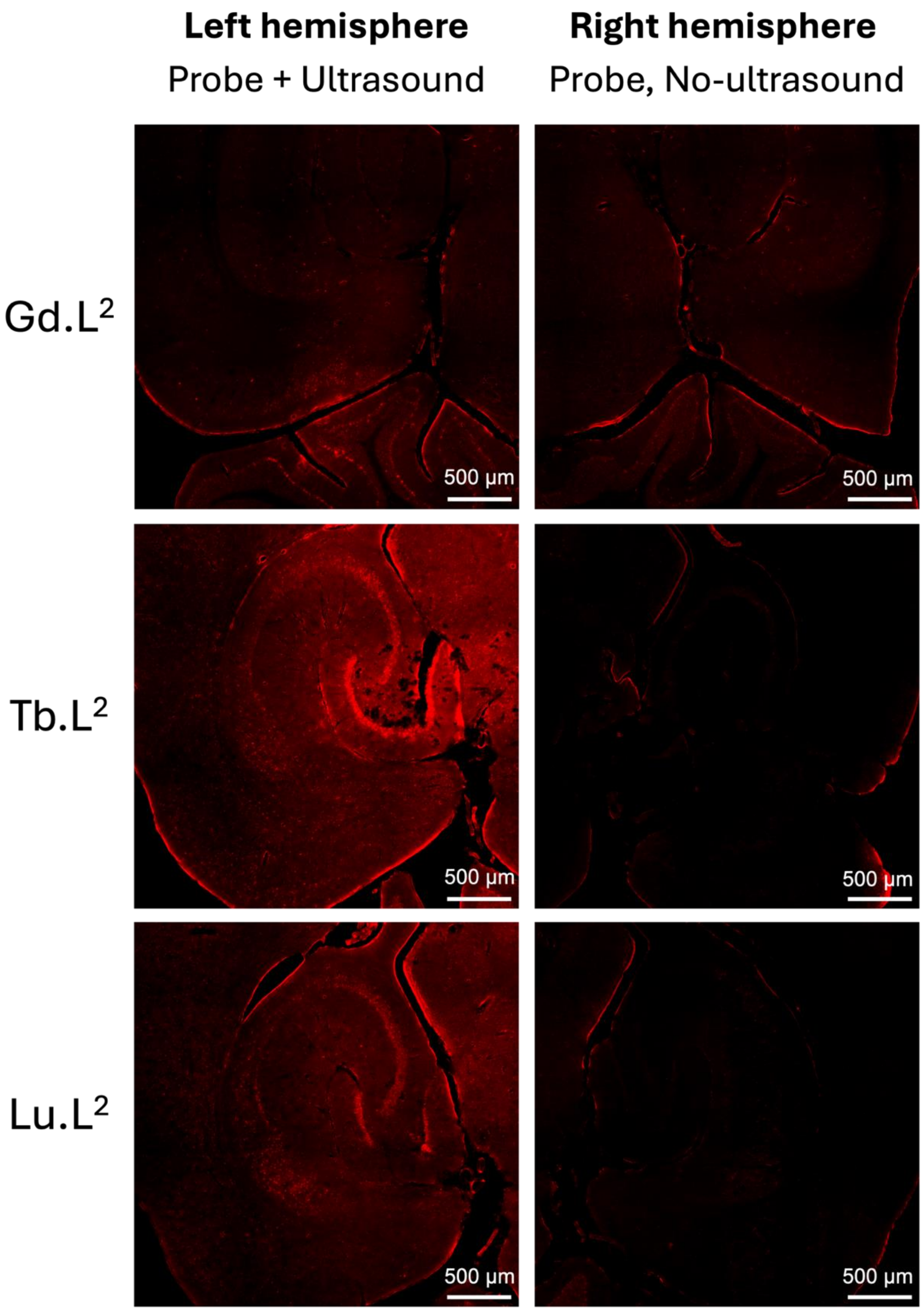

Figure S35. Fluorescence microscopy images of representative brain sections from mice injected with Ln.L^{2} *complexes. The left hippocampus was treated with ultrasound and fluorescence was observed in the tissues. No fluorescence was observed in the contralateral right hippocampus (control region – no ultrasound exposure).*

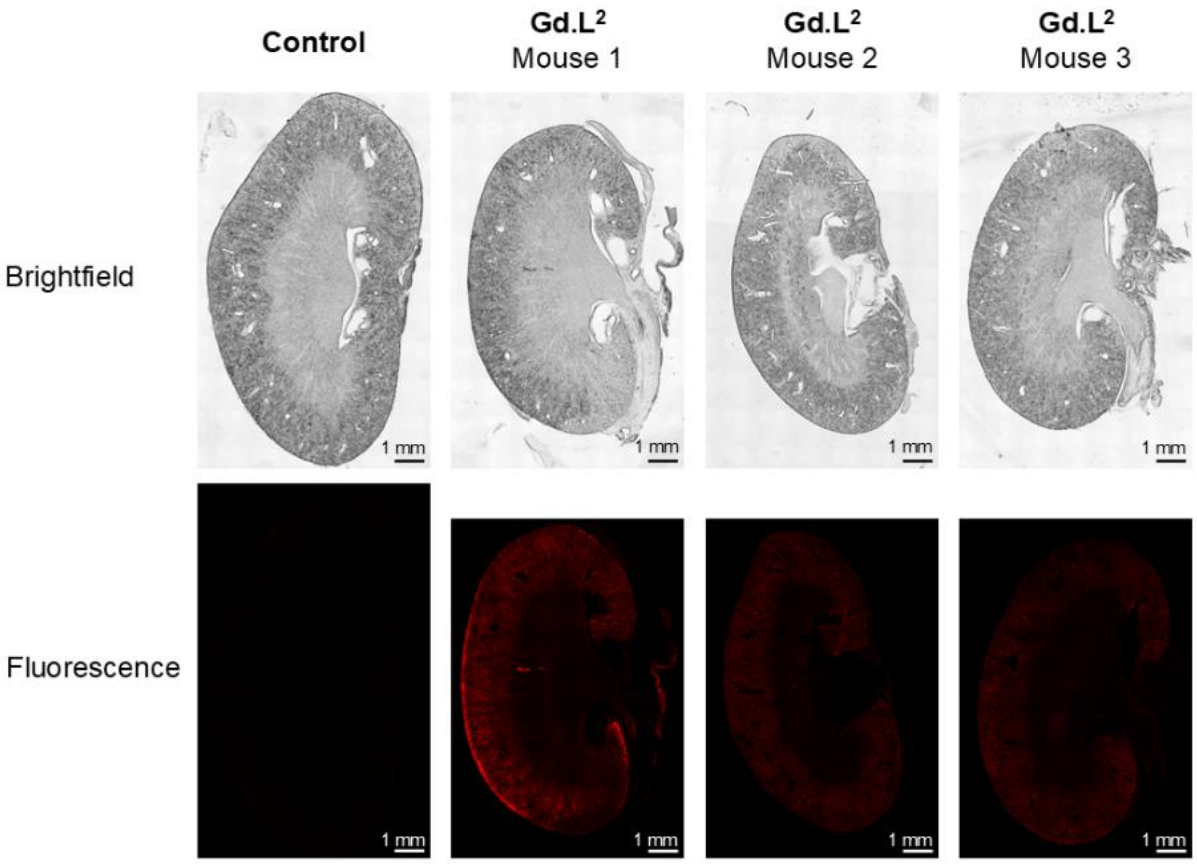

Figure S36. Brightfield and fluorescence channel images of representative sections from the kidneys of 3 mice injected with Gd.L^2 *and treated with focused ultrasound and microbubbles compared to a control mouse that was not injected with the probe.*

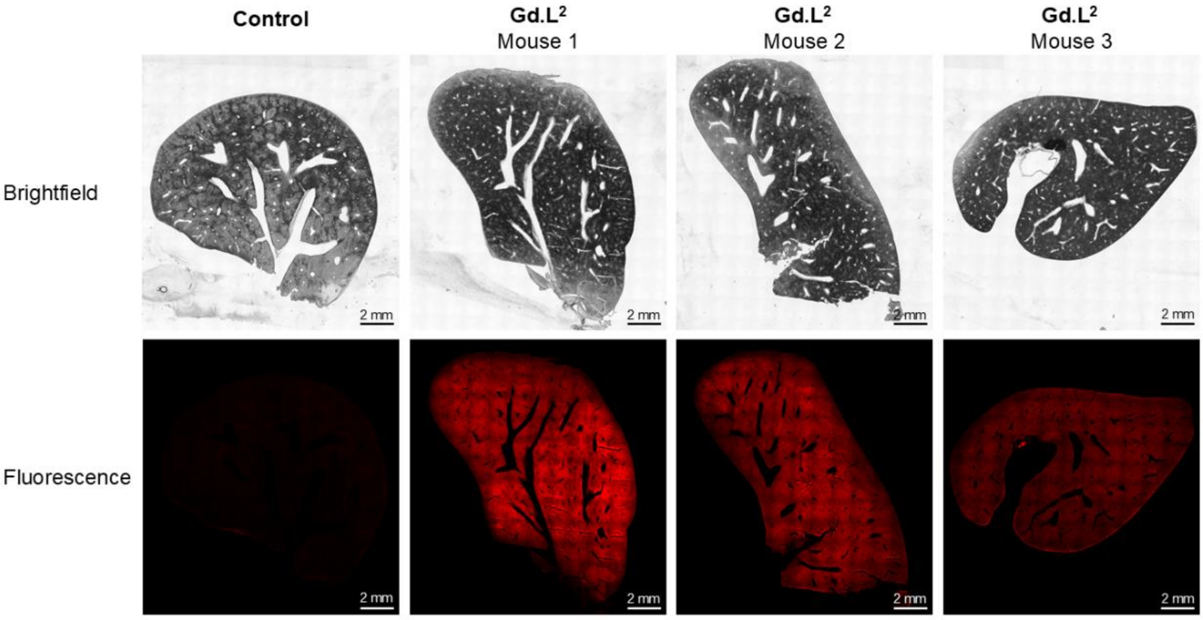

Figure S37. Brightfield and fluorescence images of a slice from the livers of 3 mice treated with Gd.L^2 *and treated with focused ultrasound and microbubbles compared to a control mouse that was not injected with the probe.*

9. Histology

Fixed brain sections mounted on glass slides were subjected to heat-mediated antigen retrieval by incubation in 10% citrate buffer (prepared in deionised H_2O) at 80 °C for 1 minute. Slides were then allowed to cool to 25 °C over 20 minutes, followed by three washes in phosphate-buffered saline (PBS), each for 5 minutes. Permeabilisation was performed using 0.1% Triton X-100 in PBS for 30 minutes. Slides were subsequently washed again in PBS (3 × 5 min) and blocked for non-specific binding using a blocking solution composed of 5% goat serum, 1% bovine serum albumin (BSA), and 0.1% Triton X-100 in PBS, applied for 1 hour at 25 °C.

Primary antibody incubation was performed overnight at 4 °C using recombinant rabbit anti-NeuN antibody [EPR12763] (1:500, ab177487, Abcam Cambridge, UK), diluted in PBS containing 0.01% Triton X-100 and 0.1% BSA. The following day, slides were washed in PBS (3 × 5 min) and incubated with the secondary antibody goat anti-rabbit IgG H&L (1:500, Alexa Fluor® 488, Abcam, ab150077), diluted in 0.01% Triton X-100 and 0.1% BSA in PBS for 2 hours at 25 °C. After final PBS washes (3 × 5 min), slides were mounted using a DAPI-containing mounting medium (Fluoroshield™ Mounting Medium With DAPI, ab104139, Abcam, Cambridge, UK) and coverslipped. Coverslips were sealed with clear nail polish, and slides were stored at 4 °C until imaging.

Stained brain sections were imaged by fluorescence widefield microscopy (20x/0.8 DIC Plan Apochromat; working distance = 0.55 mm; Axio Observer; ZEISS; Oberkochen, Germany) with excitation at 470/40 nm (Spectra X Light Engine, Lumencor, Beaverton, OR, USA) and emissions detected at 525/50 nm.

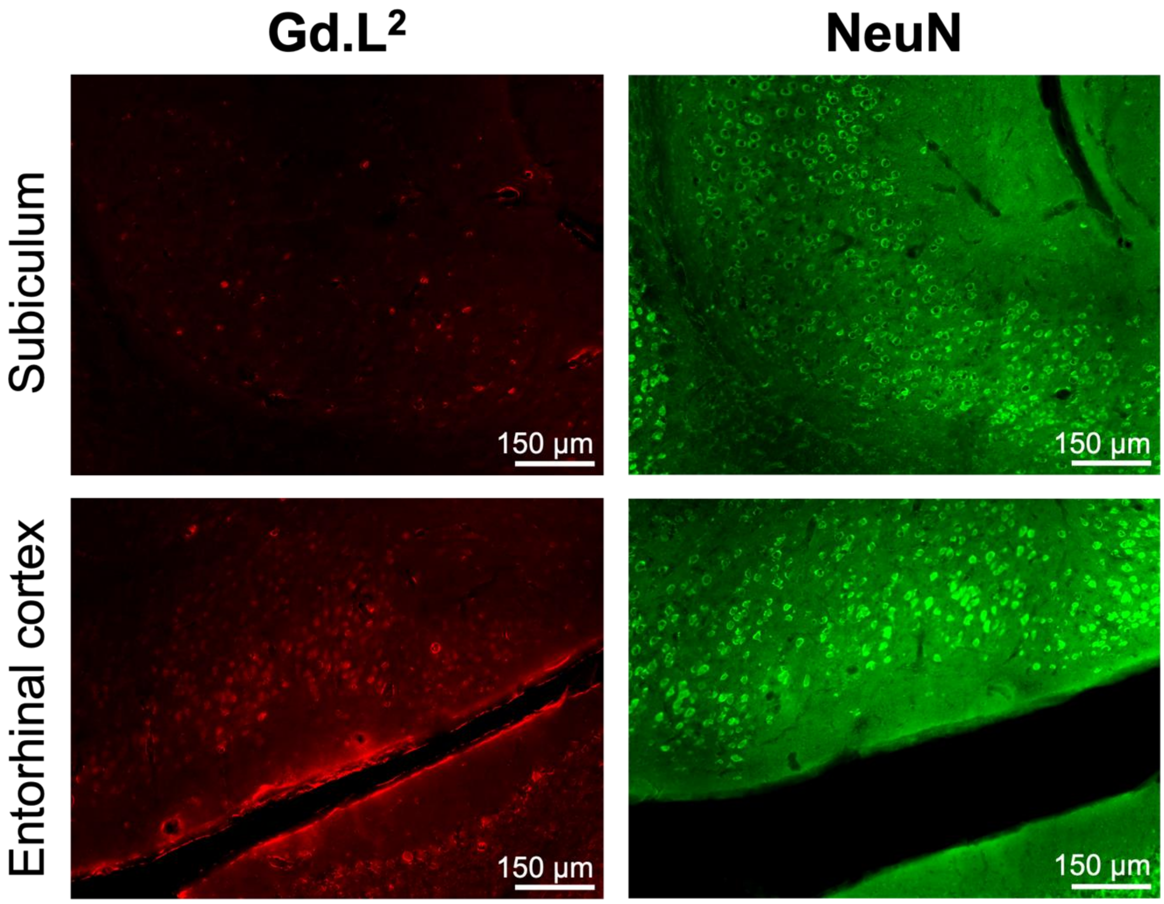

Figure S38. (Left) Fluorescence images, prior to immunofluorescence staining, of horizontal 30 µm-thick frozen brain sections from mice injected with Gd.L^2 and treated with focused ultrasound and microbubbles and (Right) NeuN-positive staining of horizontal 30 µm-thick frozen sections from the same brain.

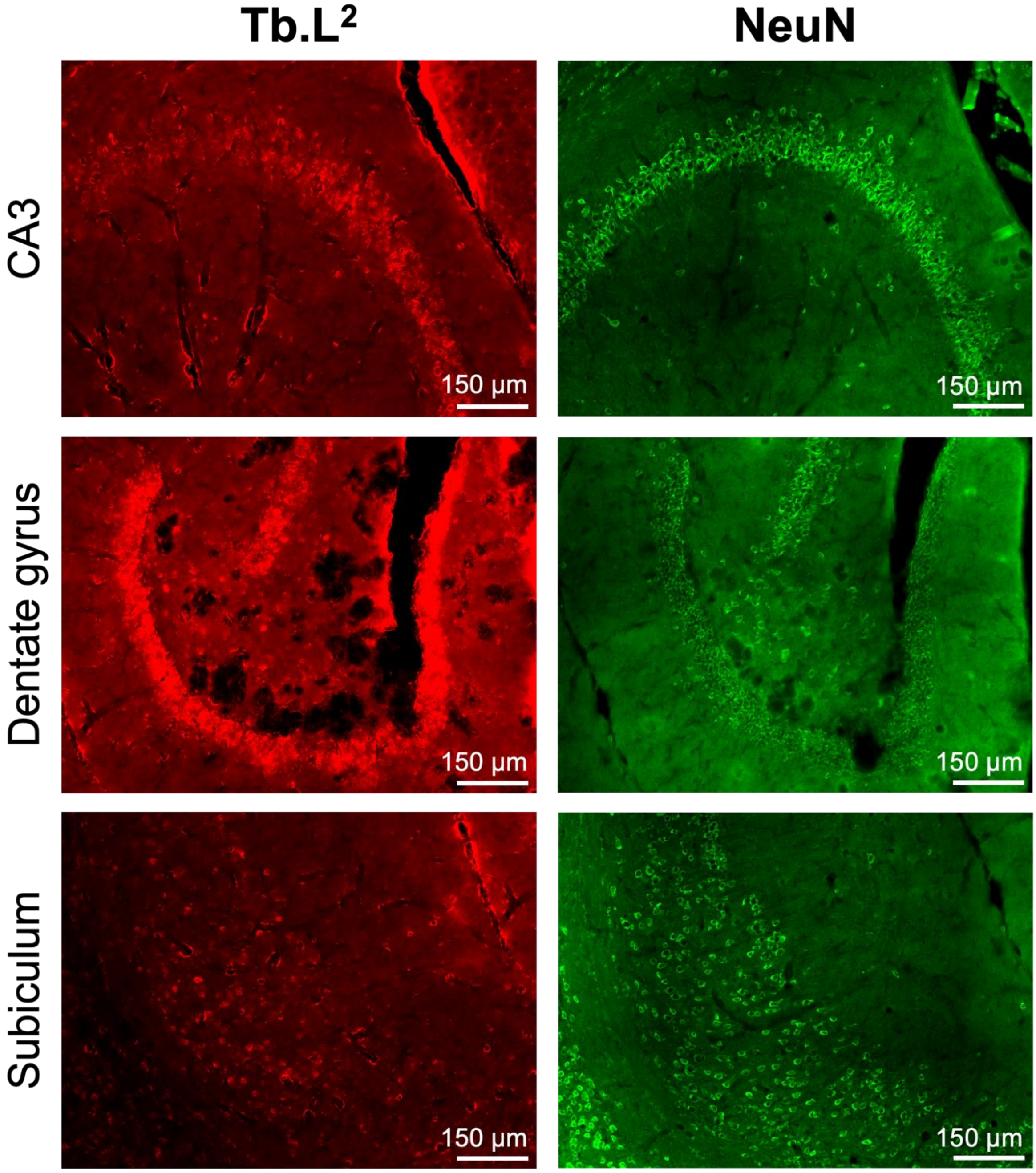

Figure S39. (Left) Fluorescence images, prior to immunofluorescence staining, of horizontal 30 µm-thick frozen brain sections from mice injected with Tb.L^2 and treated with focused ultrasound and microbubbles and (Right) NeuN-positive staining of horizontal 30 µm-thick frozen sections from the same brain.

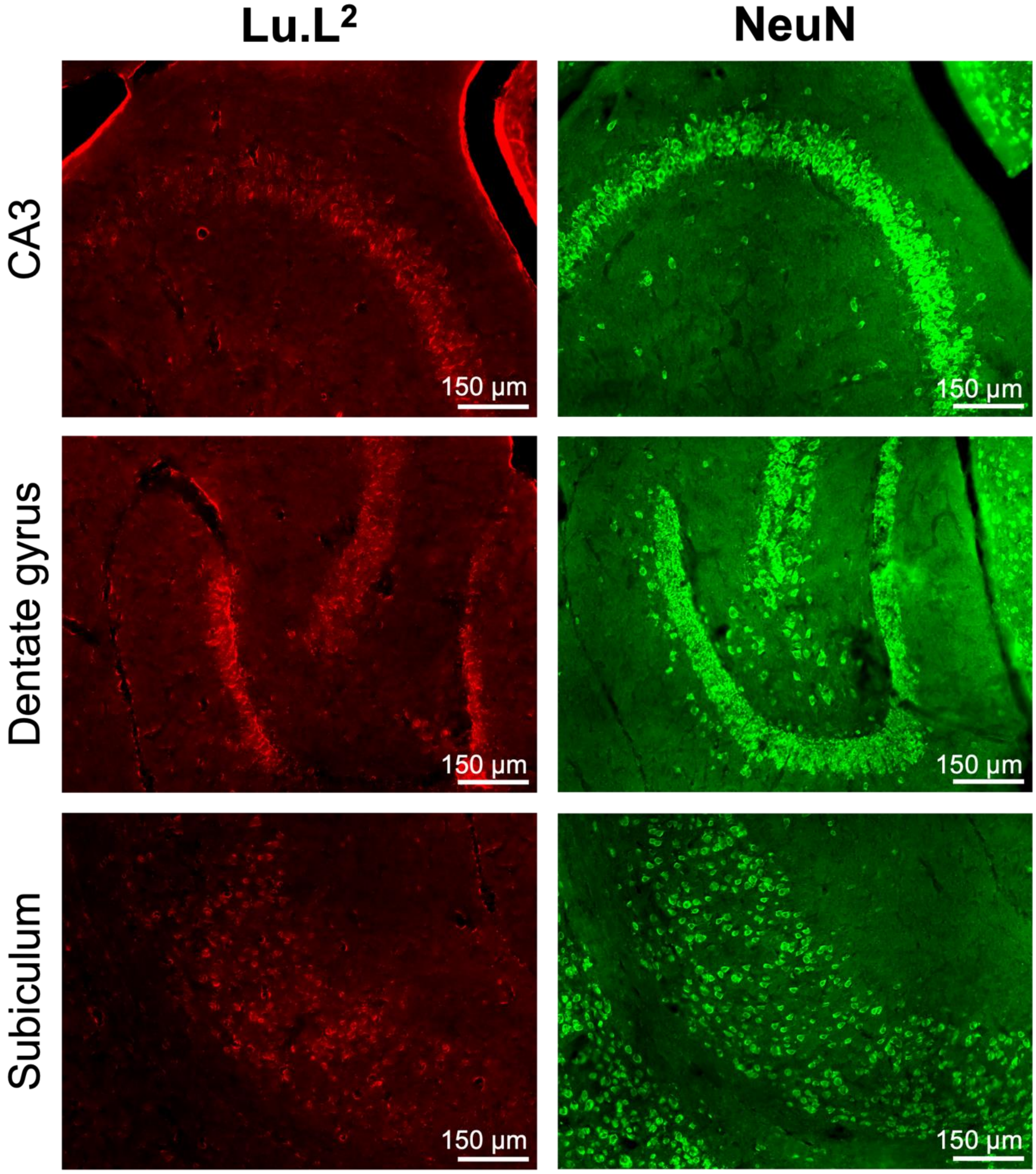

Figure S40. (Left) Fluorescence images, prior to immunofluorescence staining, of horizontal, 30 µm-thick frozen brain sections from mice injected with Lu.L^2 and treated with focused ultrasound and microbubbles and (Right) NeuN-positive staining of horizontal 30 µm-thick frozen sections from the same brain.

10. References

1. D. F. Evans, The determination of the paramagnetic susceptibility of substances in solution by nuclear magnetic resonance, *J. Chem. Soc.*, 1959, 2003-2005.

2. D. M. Corsi, C. Platas-Iglesias, H. van Bekkum and J. A. Peters, Determination of paramagnetic lanthanide(III) concentrations from bulk magnetic susceptibility shifts in NMR spectra, *Magn. Reson. Chem.*, 2001, **39**, 723-726.

3. J. Hernández-Gil, M. Braga, B. I. Harriss, L. S. Carroll, C. H. Leow, M.-X. Tang, E. O. Aboagye and N. J. Long, Development of ^{68}Ga-labelled ultrasound microbubbles for whole-body PET imaging, *Chem. Sci.*, 2019, **10**, 5603-5615.

4. J. Yang, M. R. Karver, W. Li, S. Sahu and N. K. Devaraj, Metal-catalyzed one-pot synthesis of tetrazines directly from aliphatic nitriles and hydrazine, *Angew. Chem., Int. Ed.*, 2012, **51**, 5222-5225.

5. B. Woolley, Y. Wu, L. Xiong, H.-F. Chau, J. Zhang, G.-L. Law, K.-L. Wong and N.J. Long, Lanthanide-tetrazine probes for bio-imaging and click chemistry, *Chem. Sci.*, 2025, **16**, 3588-3597.

6. B. Jagadish, G. L. Brickert-Albrecht, G. S. Nichol, E. A. Mash and N. Raghunand, On the synthesis of 1,4,7-tris(tert-butoxycarbonylmethyl)-1,4,7,10-tetraazacyclododecane, *Tetrahedron Lett.*, 2011, **52**, 2058-2061.

7. L. Bi, WIPO, Michigan Technological University, WO2014/063033 A2, 2014.

8. C. Kilkenny, W. Browne, I. C. Cuthill, M. Emerson and D. G. Altman, Animal research: reporting in vivo experiments: the ARRIVE guidelines, *Br. J. Pharmacol.,* 2010, **160**, 1577-1579.

9. N. M. Powell, M. Modat, M. J. Cardoso, D. Ma, H. E. Holmes, Y. Yu, J. O'Callaghan, J. O. Cleary, B. Sinclair, F. K. Wiseman, V. L. J. Tybulewicz, E. M. C. Fisher, M. F. Lythgoe and S. Ourselin, Fully-automated μMRI morphometric phenotyping of the Tc1 mouse model of Down syndrome, *PLOS One*, 2016, **11**, e0162974.
